# Antennal transcriptome analysis of olfactory genes and characterizations of odorant binding proteins in two woodwasps, *Sirex noctilio* and *Sirex nitobei* (Hymenoptera: Siricidae)

**DOI:** 10.1186/s12864-021-07452-1

**Published:** 2021-03-10

**Authors:** Bing Guo, Enhua Hao, Haili Qiao, Jingzhen Wang, Weiwei Wu, Jingjiang Zhou, Pengfei Lu

**Affiliations:** 1 The Key Laboratory for Silviculture and Conservation of the Ministry of Education, School of Forestry, Beijing Forestry University, 35 Qinghua Dong Road, Haidian District, Beijing, 100083 People’s Republic of China; 2 Institute of Medicinal Plant Development, Chinese Academy of Medical Sciences and Peking Union Medical College, Beijing, 100193 People’s Republic of China

**Keywords:** Woodwasps, Transcriptome, Olfactory genes, Expression profiles

## Abstract

**Background:**

The woodwasp *Sirex noctilio* Fabricius is a major quarantine pest worldwide that was first discovered in China in 2013 and mainly harms *Pinus sylvestris* var. *mongolica* Litv.. *S. nitobei* Matsumura is a native species in China and is closely related to *S. noctilio*. Recently, the two woodwasps species were found attacking the *P. sylvestris* var. *mongolica* Litv in succession. The olfactory system is the foundation of insect behavior. Olfactory genes were identified through antennal transcriptome analysis. The expression profiles odorant binding proteins (OBPs) were analyzed with RT-qPCR.

**Results:**

From our transcriptome analysis, 16 OBPs, 7 chemosensory proteins (CSPs), 41 odorant receptors (ORs), 8 gustatory receptors (GRs), 13 ionotropic receptors (IRs), and one sensory neuron membrane protein (SNMP) were identified in *S. noctilio*, while 15 OBPs, 6 CSPs, 43 ORs, 10 GRs, 16 IRs, and 1 SNMP were identified in *S. nitobei*. Most of the olfactory genes identified in two species were homologous. However, some species-specific olfactory genes were identified from the antennal transcriptomes, including *SnocOBP13*, *SnocCSP6*, *SnocOR26, SnocGR2*, *SnocIR7* in *S. noctilio* and *SnitGR9, SnitGR11, SnitIR17* in *S. nitobei.* In total, 14 *OBPs* were expressed primarily in the antennae. *SnocOBP9* and *SnitOBP9,* identified as *PBP* homologues, were sex-biased expression in two siricid, but with different pattern. *SnocOBP11* and *SnitOBP11* were highly expressed in antennae and clearly expressed in external genitalia. *SnocOBP7* and *SnitOBP7* were highly expressed in male genitalia. *SnocOBP3* and *SnocOBP10* were highly expressed in female genitalia and male heads, while *SnitOBP3* and *SnitOBP10* did not show obvious tissue bias.

**Conclusion:**

We analyzed 86 and 91 olfactory genes from *S. noctilio* and *S. nitobei*, respectively. Most of the olfactory genes identified were homologous, but also some species-specific olfactory genes were identified, which indicated the similarities and differences of the molecular mechanisms between the two closely-related species. Different expression in the antennae, external genitals or heads, exhibiting an obvious sex bias, suggested their different role in recognizing sex pheromones or plant volatiles. Species-specific expression for several OBPs genes may suggest that they strengthened or lost their original function during species differentiation, resulting in olfactory differences between the two species.

**Supplementary Information:**

The online version contains supplementary material available at 10.1186/s12864-021-07452-1.

## Background

Siricid woodwasps (Hymenoptera: Siricidae) are insects which mainly harm *Pinus* trees [1, 2]. As wood-boring insects, the woodwasps feed on wood during the larval period of their development. Adult woodwasps do not feed and only live for approximately a week [3, 4]. Female adults tend to attack stressed or weakened pines, where they lay eggs and inject toxic mucus and symbiotic fungus into the host [5, 6]. The affected pine trees fall into decline and display symptoms such as resinosis, interior blue staining, premature senescence, reduced growth rates, and death [7].

*Sirex noctilio* Fabricius belongs to such woodwasps. It is native to Europe, Asia, and North Africa, and is attracted to dead or dying pines [5, 8, 9]. Due to increased human movement and trade, the woodwasp has spread to Oceania, Africa, North America, and South America and become a globally invasive insect species [10]. Because of the lack of competing species and natural predators, *S. noctilio* has had a major economic impact on various pines in invaded areas [10]. In August 2013, *S. noctilio* was first found in Heilongjiang and then in Liaoning, Jilin, and Inner Mongolia, China [11]. In contrast, *S. nitobei* Matsumura*,* a species closely related to *S. noctilio*, is native to China, Japan, and North Korea. It has posed a hazard on ancient and debilitated pines such as the *Pinus tabuliformis* in Xiangshan Park, Beijing, China [12] and is a significant threat to other pine species such as *P. armandi* and *Larix* spp. [13]. The morphology of the two species, *S. noctilio* and *S. nitobei*, are very similar. The difference between two woodwasps is the different colors of their abdomen and hindfoot. Recently, the two woodwasps species were reported attacking the *P. sylvestris* var. *mongolica* Litv. from June to September from 2016 to 2019 successively, in Jinbaotun town, Inner Mongolia Autonomous Region, China, within a year [14]. *S. noctilio* adults started to emerge in the field from late June to early September, subsequently, emerge peak of *S. nitobei* was found from same trees during late August and late September. Both of them are associated with the same fungal symbiont, *Amylostereum areolatum* in China [11].

In order to reduce the spread of and damage inflicted by woodwasps, it is important to develop effective detection tools to monitor their populations. Trap trees treated with herbicide or girdling have been used to monitor and survey *S. noctilio* populations [9, 15]. The trap-tree method has been found to be effective but is expensive and difficult to implement. Kairomone (plant volatiles) lure traps are the most effective in areas where *S. noctilio* populations are large [16]. Pheromones are also commonly used to develop attractants, and several pheromone compounds for *S. noctilio* have been discovered [17, 18]. In China, as a native species, *S. nitobei* remain relatively understudied and poorly understood, owing to their low abundance and habit of attacking only dead or highly stressed trees with little economic value. But, recently, *S. nitobei* has attracted attention due to its sympatric coexistence with the quarantine pest, *S. noctilio.* Field monitoring using attractants was carried out. Some traps with above mentioned lures for *S. noctilio*, either plant volatiles or pheromone, were also attractive to *S. nitobei*, but with different efficiency [19]. In a word, it is possible that chemical cues used by the two woodwasps are different to some extend.

Insects use their olfactory systems to sense odors and changes in the environment and thus, to adjust behaviors such as locating hosts for food, mating, and oviposition [20]. The antenna is the most important olfactory organ for recognition and sensing of pheromones or plant volatiles. There are multiple olfactory sensilla distributed on insect antennae, which house olfactory sensory neurons (OSNs). Odor molecules pass through pores on the sensilla and enter the sensillum lymph [21, 22]. It has been thought that odorant binding proteins (OBPs) and chemosensory proteins (CSPs) in the lymph can recognize, bind, and transport odor molecules. The OBP/CSP-odor molecule complexes then interact with chemosensory receptors, which are located in the dendritic membrane of OSNs [23, 24]. Chemosensory receptors are transmembrane proteins and include odorant receptors (ORs), ionotropic receptors (IRs), gustatory receptors (GRs), and sensory neuron membrane proteins (SNMPs). These receptors and OSNs convert the chemical signals into electrophysiological signals and transmit these signals to the central nervous system of insects through axons [25, 26]. These signals are integrated in the insect brain to produce behavioral instructions for insects to respond accordingly [27]. At the same time, odor molecules are degraded by odorant-degrading enzymes (ODEs) [28, 29].

The objective of our study is to identify the chemosensory genes of the two species in order to characterize chemosensation in siricid species. We explored the woodwasp olfaction system, particularly, olfactory proteins and their expression profiles in antennae. We identified genes encoding olfactory proteins via analysis of the antennal transcriptomes of *S. noctilio* and *S. nitobei* and measured the transcript expression of important OBP genes in different tissues of both male and female adults of the two woodwasps using a quantitative real-time PCR method. Our study is the first description of differential expression profiles of OBPs between different tissues and between sexes for these two wasps. Taken together, our findings identified and compared olfactory genes in the two woodwasps based on antennal transcriptome analysis, and established a start point for further research on molecular mechanisms of olfactory system in symphyta woodwasps.

## Results

### Transcriptome sequencing and sequence assembly

Using transcriptome sequencing, a total of 174,174,820 and 168,012,792 raw reads were obtained from male and female antennae, respectively, of *S. noctilio*, and a total of 165,394,906 and 164,334,008 raw reads were obtained from male and female antennae, respectively, of *S. nitobei* (Additional file 1, Table S1). By removing low-quality and trimmed reads less than 20 nt in length, 168,575,526 and 164,447,898 clean reads were obtained for male and female *S. noctilio*, respectively, and 161,515,996 and 160,823,260 clean reads were obtained for male and female *S. nitobei*, respectively, to be used for de novo assembly (Additional file 1, Table S2). The clean reads from *S. noctilio* were assembled into 47,253 unigenes with a total length of 61,586,545 base pairs (bp), an average length of 1303 bp, and a maximum length of 56,024 bp. The sequence length distribution analysis indicated that 16,625 unigenes (35.18%) were longer than 1000 bp (Additional file 1, Table S3). The clean reads from *S. nitobei* were assembled into 46,866 unigenes with a total length of 55,062,400 bp (Additional file 1, Table S4) and an average length of 1175 bp. The unigenes ranged from 201 bp - 39,567 bp in length, and 13,634 of the unigenes are > 1000 bp. The raw reads for *S. noctilio* and *S. nitobei* were deposited in the NCBI SRA database (the accession number of *S. noctilio* are from SAMN11338151 to SAMN11338156 and the accession number of *S. nitobei* are from SAMN11338569 to SAMN11338574).

### Homology analysis and gene ontology annotation

In total, 20,053 unigenes from *S. noctilio* (42.44% of 47,253 unigenes) were annotated in at least one of the databases searched (Nr, Pfam, KOG, COG, Swiss-Prot, KEGG, eggNOG, and GO databases). Homology searches against the Nr database showed that the *S. noctilio* antennal transcriptome shared the greatest homology with sequences from *Apis mellifera* (13%), followed by *Nasonia vitripennis* (11%) and *Harpegnathos saltator* (10%). For the *S. nitobei* transcriptome, 25,278 unigenes (53.94% of 46,866 unigenes) were annotated in at least one of the databases. Nr database homology searches showed that the *S. nitobei* antennal transcriptome shared the greatest homology with sequences from *A. mellifera* (9.17%), followed by *N. vitripennis* (8.20%) and *Megachile rotundata* (7.27%).

**Fig. 1:**
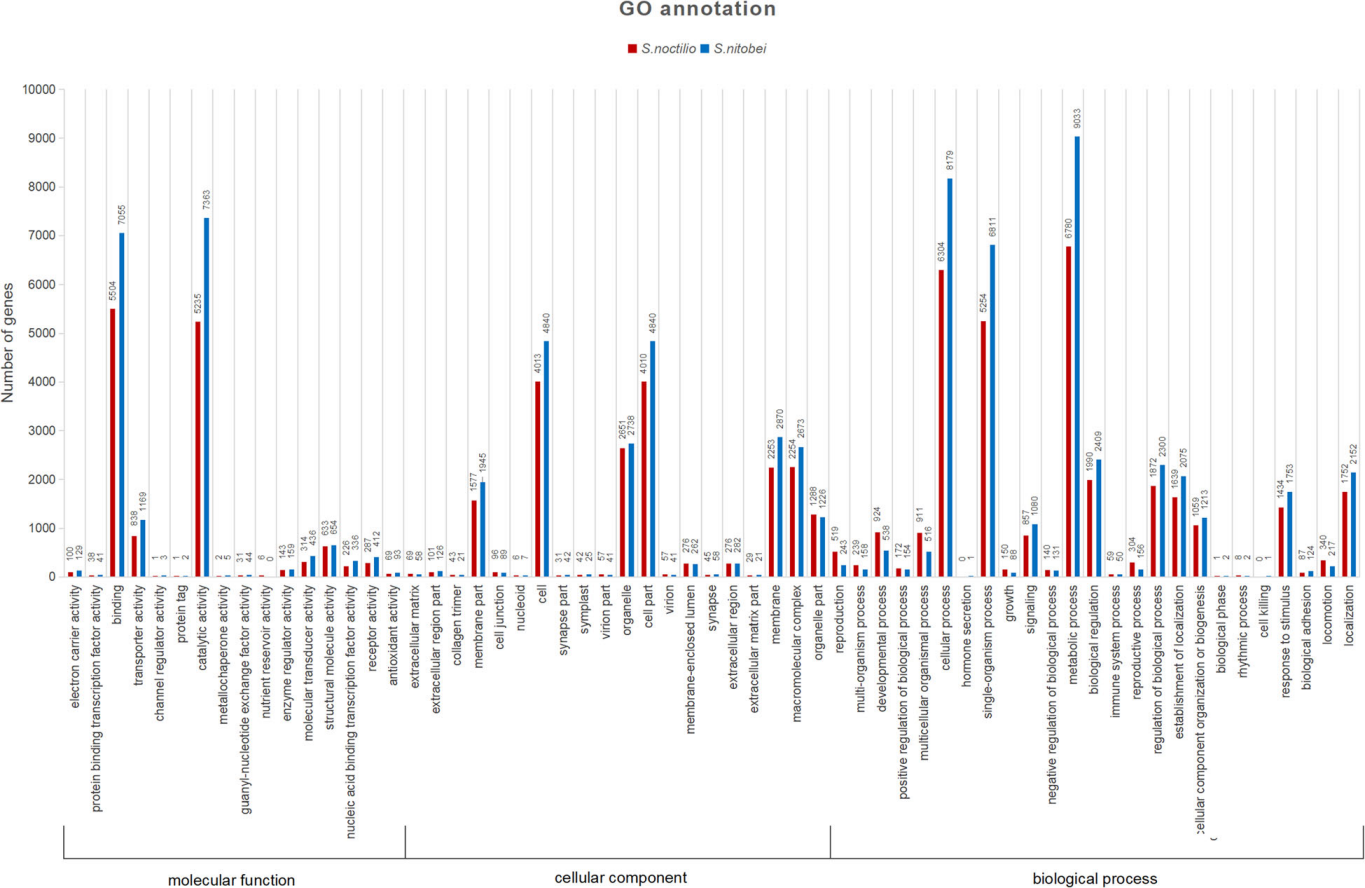
Gene ontology (GO) classification showed by quantity of *S. noctilio* and *S. nitobei* unigenes obtained using the Blast2GO program

**Fig. 2:**
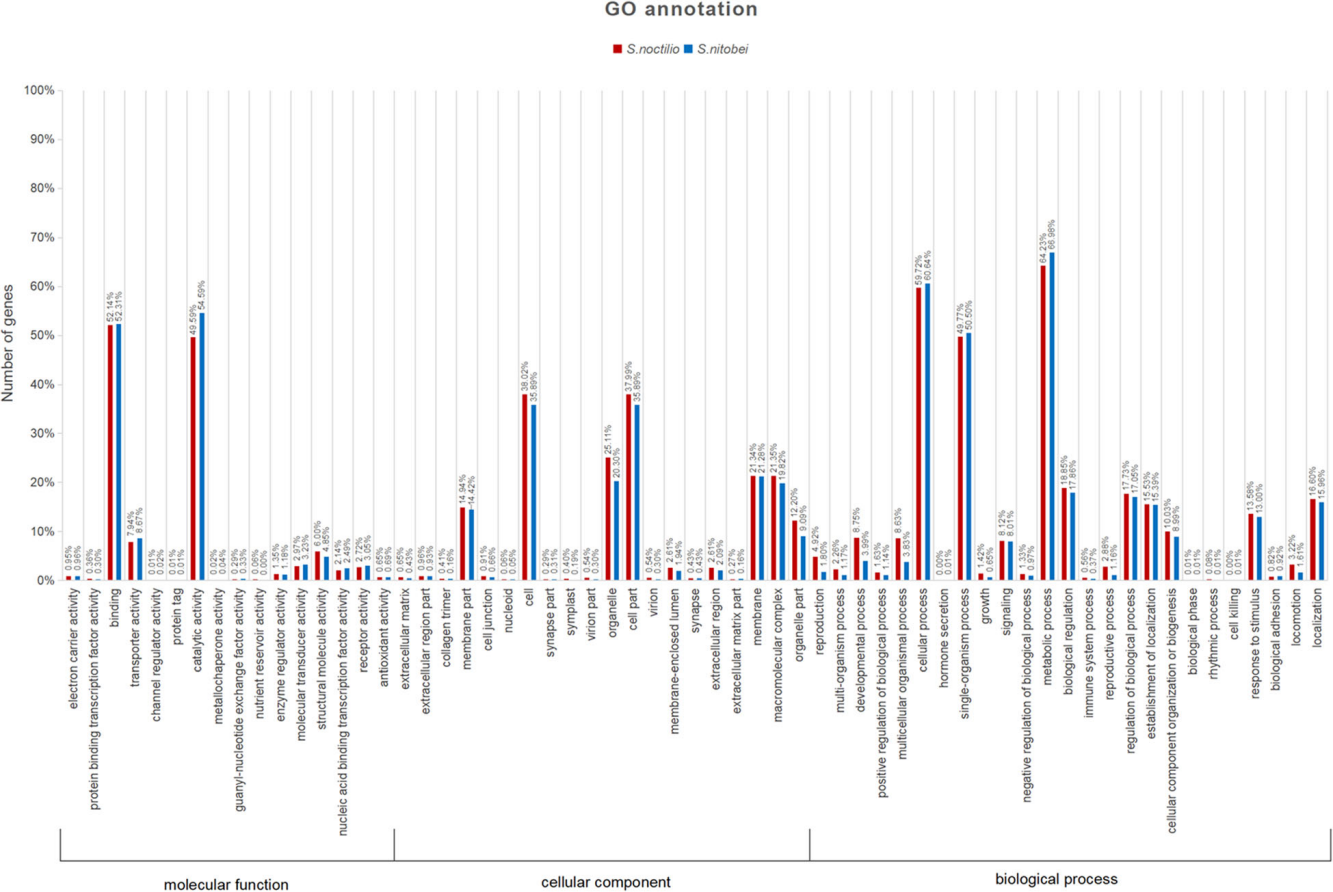
Gene ontology (GO) classification showed by percentage of *S. noctilio* and *S. nitobei* unigenes obtained using the Blast2GO program

Among the 47,253 *S. noctilio* and 46,866 *S. nitobei* unigenes, 10,556 (22.3%) and 13,487 (28.8%), respectively, correspond to at least one GO term. GO annotation indicated that the distributions of GO terms in the unigenes were highly similar between the two species (Figs. 1 and 2). The similar result was found in the GO annotation between *Helicoverpa armigera* and *H. assulta* [30]. Within the biological process category, the most abundant terms were ‘cellular process’ ‘single-organism process,’ and ‘metabolic process’. The ‘cell’ and ‘cell part’ were the most commonly represented of the cellular component terms. In the molecular function category, ‘binding’ and ‘catalytic activity’ were the most abundant terms.

### Identification and analysis of chemosensory-related genes

#### Odorant binding proteins (OBPs)

We identified 16 and 15 OBPs in the *S. noctilio* and *S. nitobei* antennal.

transcriptomes, respectively (Additional file 2, Table S1). Both *S. noctilio* and *S. nitobei* OBPs contained 15 full-length OBPs with complete open reading frames (ORFs) of at least 300 bp and a signal peptide (except *SnocOBP13*). According to the OBP classification system, in both species, two OBPs (*SnocOBP11* and *SnitOBP11*) were found to be members of the Minus-C OBP subclass characterized by their lack of two cysteine residues (C2 and C5). No Plus-C OBPs were found in either the *S. noctilio* or the *S. nitobei* transcriptome. Two woodwasp OBPs (*SnocOBP9* and *SnitOBP9*) were homologous to PBPs of *A. mellifera*. These two OBPs are important because they may play roles in wasps’ sexual behaviors. Five *S. noctilio* OBPs (*SnocOBP3*, *SnocOBP4*, *SnocOBP10*, *SnocOBP14* and *SnocOBP15*) and five *S. nitobei* OBPs (*SnitOBP3*, *SnitOBP4*, *SnitOBP10*, *SnitOBP14* and *SnitOBP15*) exhibited similarity with GOBPs of other insects by NCBI BLASTX. The cysteine sequence pattern of the full-length classic OBPs was found to be C1-X_26–32_-C2-X_3_-C3-X_36–42_-C4-X_8–12_-C5-X_8_-C6 (Additional file 3). We found 13 *SnocOBP*s and 14 *SnitOBP*s with expression values greater than 1 FPKM, while 6 *SnocOBP*s and 7 *SnitOBP*s exhibited expression values greater than 100 FPKM, indicating high expression of these OBPs in the antennae.

**Fig. 3:**
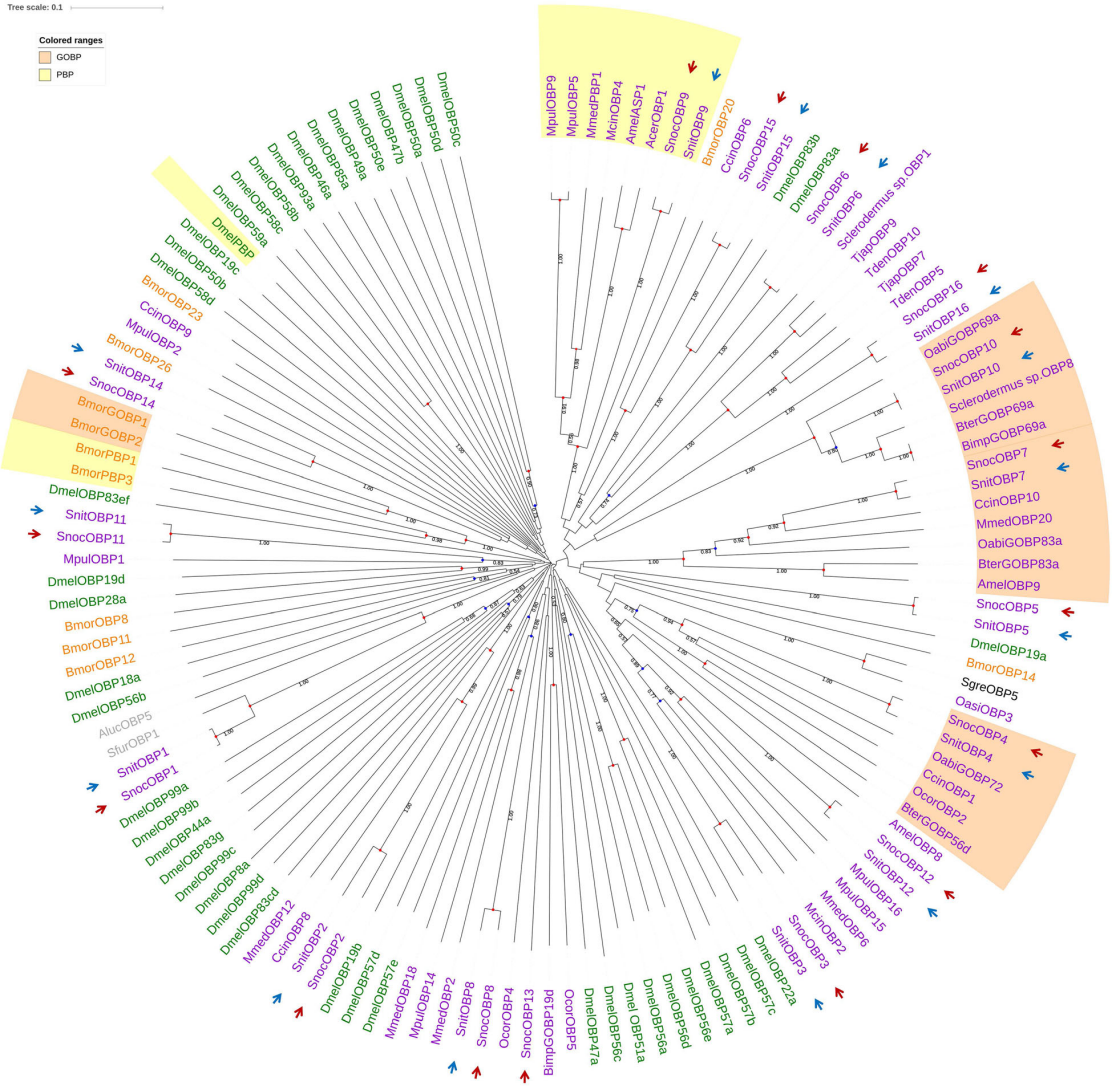
Candidate odorant binding proteins (OBPs) of Hymenoptera (purple), Diptera (green), Hemiptera (gray), Orthoptera (black) and Lepidoptera (orange) were included in a neighbor-joining phylogenetic tree. The PBP and GOBP lineages are labelled in yellow and orange, respectively. SnocOBPs and SnitOBPs are indicated with red and blue arrows, respectively

Construction of a phylogenetic tree was used to compare insect OBP protein sequences from members of the Hymenoptera, Diptera, and Lepidoptera (Fig. 3). According to the OBP phylogenetic tree, most SnocOBPs and SnitOBPs sequences clustered together. SnocOBP13 was unique to *S. noctilio* and did not cluster with other OBPs. No homologous gene had been found in *S. nitobei*. However, the FPKM value of SnocOBP13 was less than 0.001 in the transcriptome dataset, so it was hardly expressed in antennae. With a 1.00 bootstrap support value, the PBP lineages contained SnocOBP9, SnitOBP9, and other Hymenopteran PBPs, which further confirmed that the two OBPs could be PBPs. OBP4, OBP7, and OBP10 of both woodwasps were clustered in the GOBP lineages with 0.75, 1.00, and 1.00 bootstrap support values, respectively.

#### Chemosensory proteins (CSPs)

We identified 7 *SnocCSP*s and 6 *SnitCSP*s in the antennal transcriptomes of the two woodwasp species (Additional file 2, Table S2). Among all CSPs, 5 CSPs in *S. noctilio* and 4 in *S. nitobei* were full-length *CSPs* with complete ORFs, signal peptides, and a cysteine sequence pattern of C1-X_5–8_-C2-X_18_-C3-X_2_-C4 (Additional file 4). The expression values (FPKM) of 4 *SnocCSP*s and 5 *SnitCSP*s were greater than 1, while 1 *SnocCSP* and 3 *SnitCSP*s displayed expression values greater than 100, indicating that these genes were highly expressed in the woodwasp antennae.

**Fig. 4:**
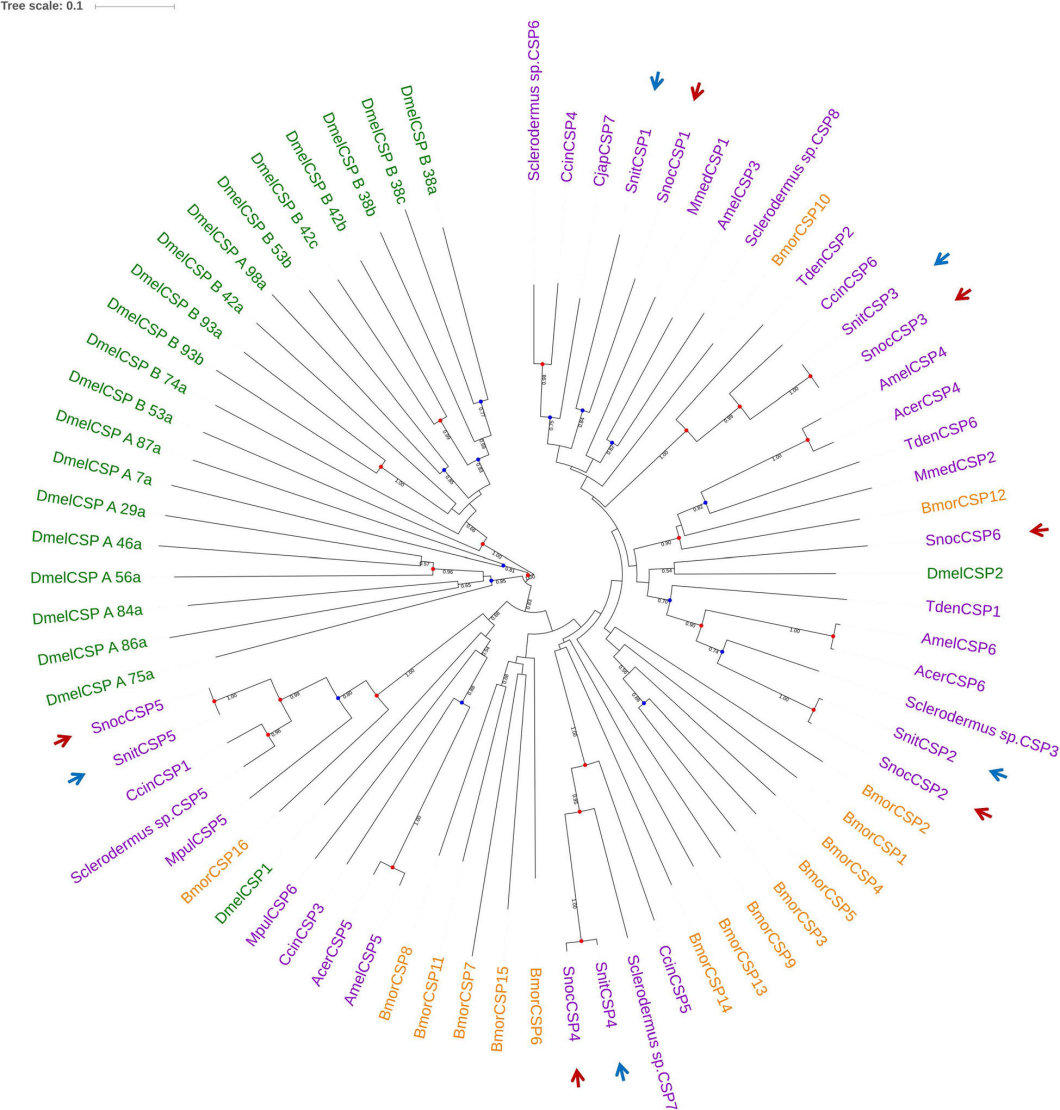
Candidate chemosensory proteins (CSPs) of Hymenoptera (purple), Diptera (green), and Lepidoptera (orange) are displayed in a neighbor-joining phylogenetic tree. SnocCSPs and SnitCSPs are marked with red and blue arrows, respectively

In the phylogenetic tree (Fig. 4), most SnocCSPs and SnitCSPs were clustered with other Hymenopteran CSPs. The homologous SnocCSP6 was not found in *S. nitobei*. The *S. noctilio* specific SnocCSP6 was clustered with DmelCSP2 in *Drosophila melanogaster* with 0.54 bootstrap support value. The FPKM value of SnocCSP6 was 0.325, so its expression was very low in *S. noctilio* antennae.

#### Odorant receptors (ORs)

We identified 41 and 43 ORs in the *S. noctilio* and *S. nitobei* antennal transcriptomes, respectively (Additional file 2, Table S3). Two woodwasp *OR* transcripts were identified as odorant co-receptors, and designated as *SnocORco* and *SnitORco*, respectively. Compared to traditional odor receptors, ORco is highly conserved, and its homology among insects can reach 50–99%. Amino acid sequence analysis revealed a highly conserved region at the end of the ORco sequence [31].

In total, 28 *SnocOR*s and 30 *SnitOR*s contained complete ORFs with more 350 amino acids, indicating that they were nearly full-length. Using the TMHMM Server, we found that 4 full-length SnocORs (SnocORco, SnocOR5, SnocOR8, and SnocOR30) and 3 full-length SnitORs (SnitORco, SnitOR8, and SnitOR30) possessed 7 transmembrane helices. No transmembrane helices were predicted in SnocOR32, SnocOR33, SnitOR31, and SnitOR32, which may be due to short fragments and incomplete reading frames. Four ORs (*SnocOR18*, *SnocOR30, SnitOR18* and *SnitOR30*) displayed a > 10-fold difference in expression between males and females, suggesting that they may play a role in identifying gender-related odors.

**Fig. 5:**
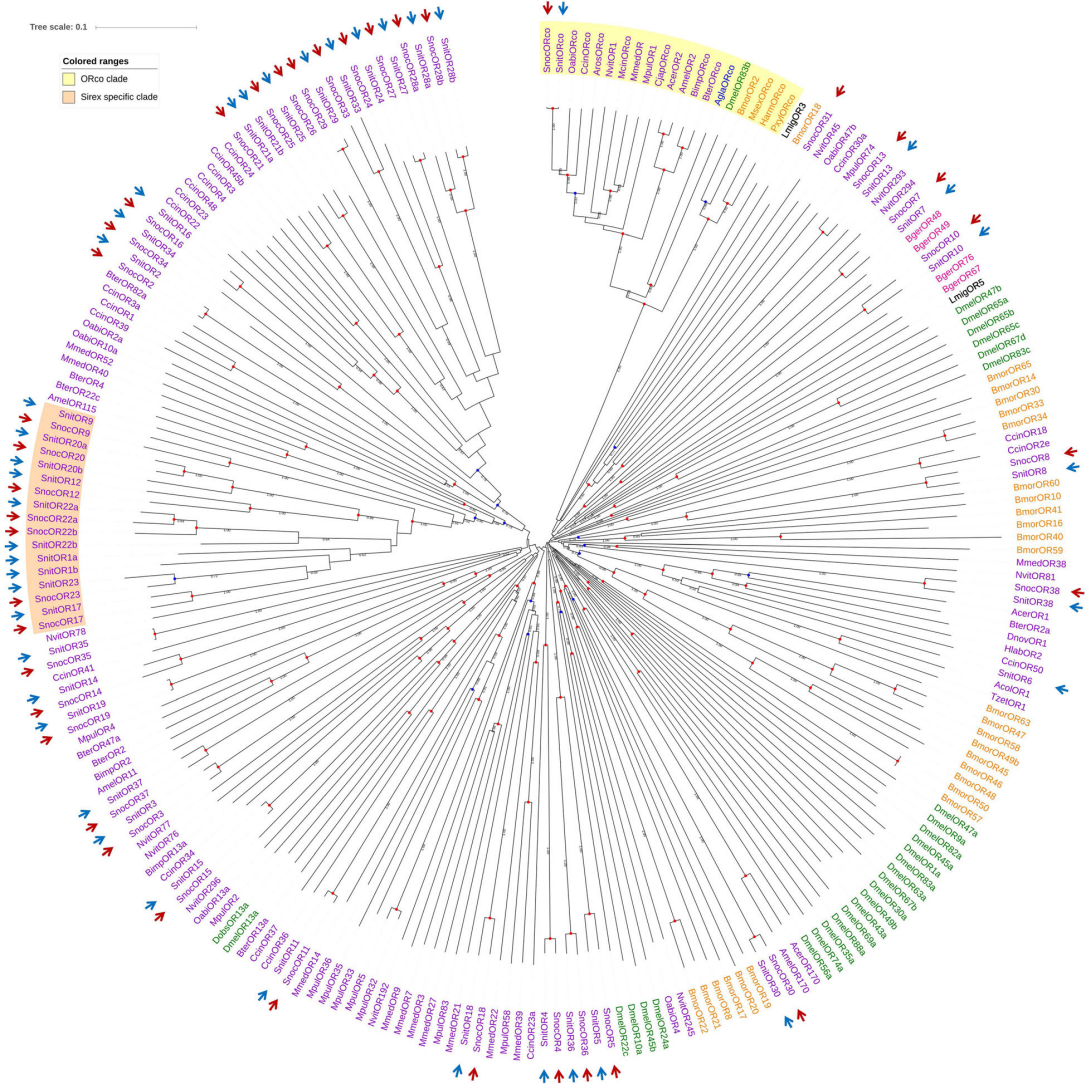
Candidate odorant receptor (ORs) of Hymenoptera (purple), Diptera (green), Lepidoptera (orange), Coleoptera (blue), Orthoptera (black), and Blattaria (pink) are displayed in a neighbor-joining phylogenetic tree. The ORco lineage and *Sirex*-specific lineages are labelled in yellow and orange. SnocORs and SnitORs are marked with red and blue arrows, respectively

Most SnocORs and SnitORs were clustered together in the phylogenetic tree (Fig. 5). Two special lineages were identified in the tree. The ORco lineage contained SnocORco and SnitORco (1.00 bootstrap support value), which further confirmed that these two ORs were ORcos. And the two ORcos were clustered with the honey bee ORco AmelOR2 [32] and other Hymenoptera ORco, suggesting they could function as a complex with other ORs in the woodwasps as the ORcos in other insects. The sirex-specific lineages contained SnocOR9, SnocOR12, SnocOR17, SnocOR20, SnocOR22a, SnocOR22b, SnocOR23 in *S. noctilio* and SnitOR1a, SnitOR1b, SnitOR9, SnitOR12, SnitOR17, SnitOR20a, SnitOR20b, SnitOR22a, SnitOR22b, SnitOR23 in *S. nitobei*. As to not-sirex-specific lineages, most of the OR genes identified in two species were homologous, but *SnocOR26* is a species-specific OR genes in *S. noctilio.*

#### Sensory neuron membrane proteins (SNMPs)

We identified one SNMP in *S. noctilio* (*SnocSNMP1*) and one in *S. nitobei* (*SnitSNMP1*) (Additional file 2, Table S4). SNMP2 could not be identified in both woodwasps species. SnocSNMP1 and SnitSNMP1 were predicted to possess 2 transmembrane regions, which may indicate that *SnocSNMP1* and *SnitSNMP1* were full-length genes. The expression values (FPKM) for *SnocSNMP1* and *SnitSNMP1* were both found to be greater than 100, indicating that *Sirex* SNMPs are highly expressed in the antennae.

**Fig. 6:**
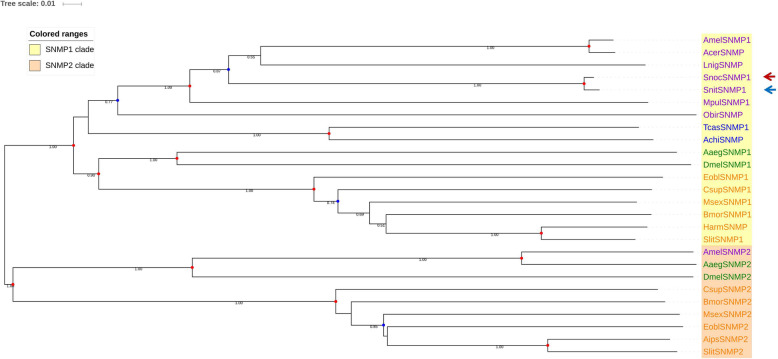
Candidate sensory neuron membrane proteins (SNMPs) of Hymenoptera (purple), Diptera (green), Lepidoptera (orange), and Coleoptera (blue) are displayed in a neighbor-joining phylogenetic tree. SnocSNMP1 and SnitSNMP1 are marked with red and blue arrows, respectively

SNMPs are considered to be highly conserved in holometabolous insects, but SNMP1 and SNMP2, which are members of different subfamilies, clustered separately in disparate lineages. In our phylogenetic tree, SnocSNMP1 and SnitSNMP1 clustered in the SNMP1 lineage with a bootstrap support value of 1.00 (Fig. 6).

#### Gustatory receptors (GRs)

We identified 8 and 10 GRs in the *S. noctilio* and *S. nitobei* antennal transcriptomes, respectively (Additional file 2, Table S5). Using BLASTX sequence alignment, we found that 2 *SnocGR*s and 7 *SnitGR*s were clustered with the GRs for sugar taste, and most were found to be trehalose receptors. No *Sirex* GRs clustered in the bitter taste lineages.

**Fig. 7:**
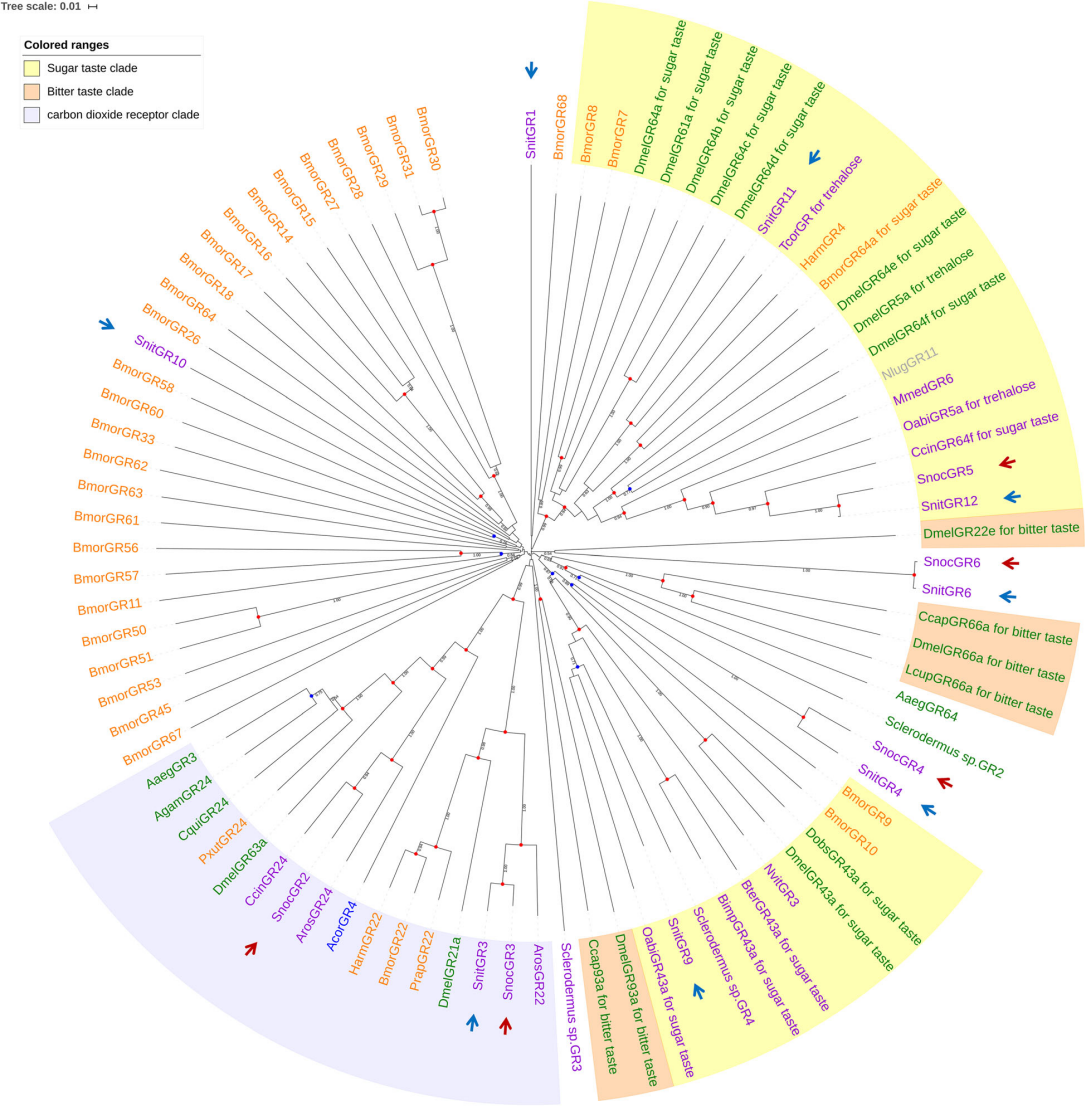
Candidate gustatory receptors (GRs) of Hymenoptera (purple), Diptera (green), Lepidoptera (orange), Coleoptera (blue), and Hemiptera (gray) are displayed in a neighbor-joining phylogenetic tree. The sugar taste lineages and bitter taste lineages have been labelled. SnocGRs and SnitGRs are marked with red and blue arrows, respectively

In the phylogenetic tree (Fig. 7) of GR sequences, there were two sugar taste lineages, three bitter taste lineages and one carbon dioxide receptor lineages. Most *Sirex* GRs exhibited homology to sugar taste receptors, and one SnocGR and 3 SnitGRs clustered in the sugar taste lineages. SnocGR5 and SnitGR5a were homologous genes and they clustered with CcinGR64f for sugar taste in *Cephus cinctus* with 0.59 bootstrap support value. SnitGR11 clustered with TcorGR for trehalose in *Trachymyrmex cornetzi* with 1.00 bootstrap support value and SnitGR9 clustered with OabiGR43a for sugar taste in *Orussus abietinus* with 0.60 bootstrap support value. These two GR had not found homologous genes in *S.noctilio*. SnocGR3 and SnitGR3 were homologous genes and they clustered with ArosGr22 for carbon dioxide receptor in *Cephus cinctus* with 0.59 bootstrap support value. SnocGR2 clustered with CcinGR24 for trehalose in *Trachymyrmex cornetzi* with 1.00 bootstrap support value. As to not-sugar taste, not-bitter taste or not-carbon dioxide recepto lineages, most of the GR genes identified in two species were homologous, but *SnitGR8* and *SnitGR10* was specific to *S. nitobei*.

#### Ionotropic receptors (IRs)

We identified 13 and 16 IRs in the *S. noctilio* and *S. nitobei* antennal transcriptomes, respectively (Additional file 2, Table S6). The expression values (FPKM) of 4 *SnocIR*s and 11 *SnitIR*s were greater than 1. Of these, *SnocIR6* and *SnitIR6* had the greatest expression in the antennal transcriptome with FPKM values of 16.359 and 21.583 respectively, which suggested that IR6 may play a major role in two woodwasps.

**Fig. 8:**
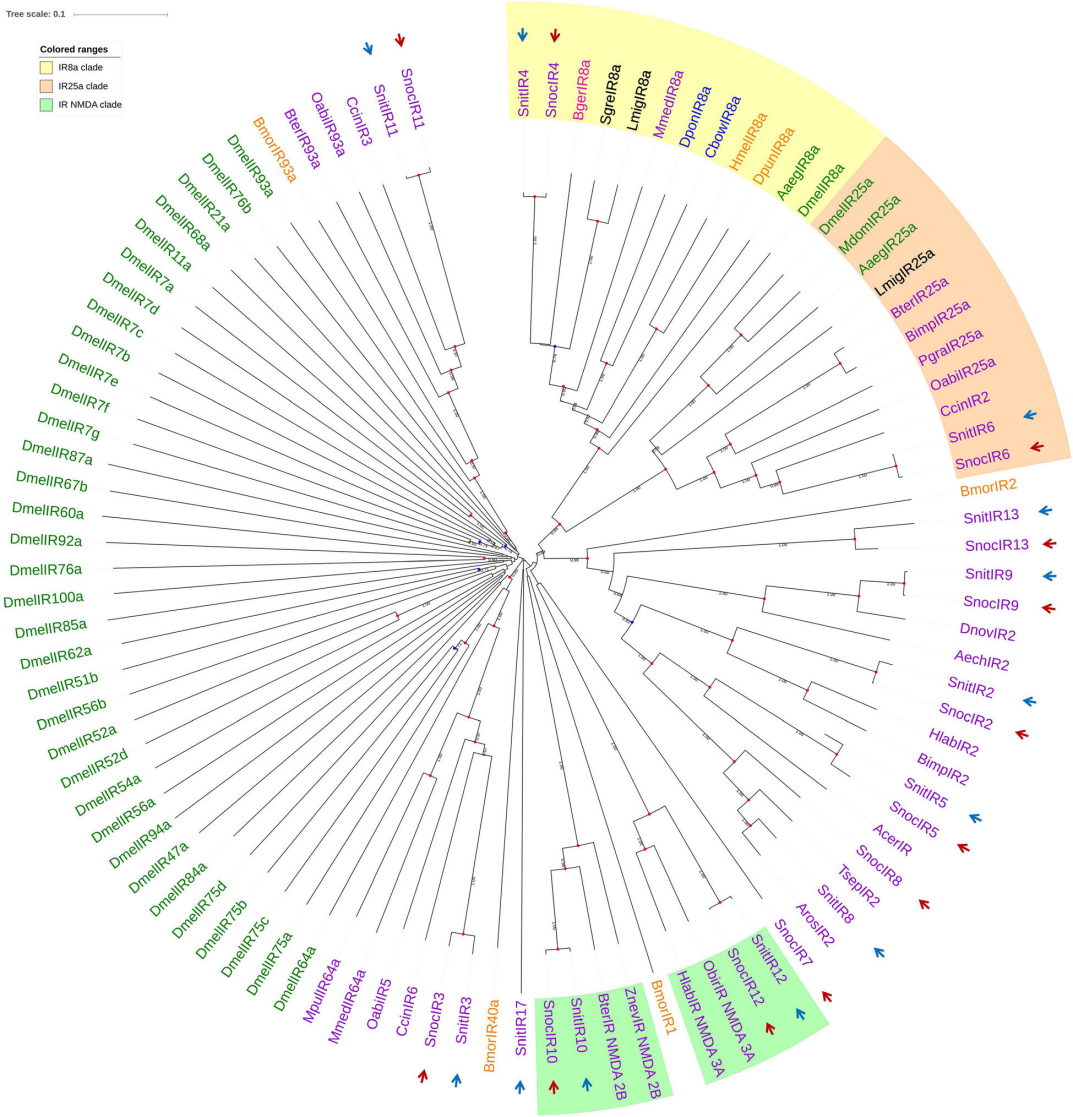
Candidate ionotropic receptors (IRs) of Hymenoptera (purple), Diptera (green), Lepidoptera (orange), Coleoptera (blue), Blattaria (pink) and Orthoptera (black) are displayed in a neighbor-joining phylogenetic tree. The IR8a lineage, IR25a lineage, and NMDA lineages have been labelled. SnocIRs and SnitIRs are marked with red and blue arrows, respectively

Previous studies have indicated that IR8a and IR25a are the co-receptors of IRs [33–35]. In our phylogenetic tree (Fig. 8), SnocIR6 and SnitIR6 clustered in the co-receptor IR8a lineages with a bootstrap support value of 1.00, while SnocIR4 and SnitIR4 clustered in the co-receptor IR25a lineages with a bootstrap support value of 1.00. IR6 and IR4 exhibited a high expression in *Sirex* transcriptomes, which may indicate that the IRs are co-receptors for IRs. Similarly, two IRco genes were found in *M. mediator* [36], while only one IR25a homolog was found in the wasp species *C. cunea* and *M. pulchricornis*. Additionally, two pairs of NMDA receptors (N-methyl-D-aspartic acid receptor) were found in the phylogenetic tree (SnocIR10, SnocIR12, SnitIR10 and SnitIR12). Among not-IRco and not-NMDA lineages, most of the IR genes identified in two species were homologous, but *SnocIR7* and *SnitIR17* were specific to *S. noctilio* and *S. nitobei,* respectively.

### Clusters of olfactory system genes in *S. noctilio* and *S. nitobei*

**Fig. 9:**
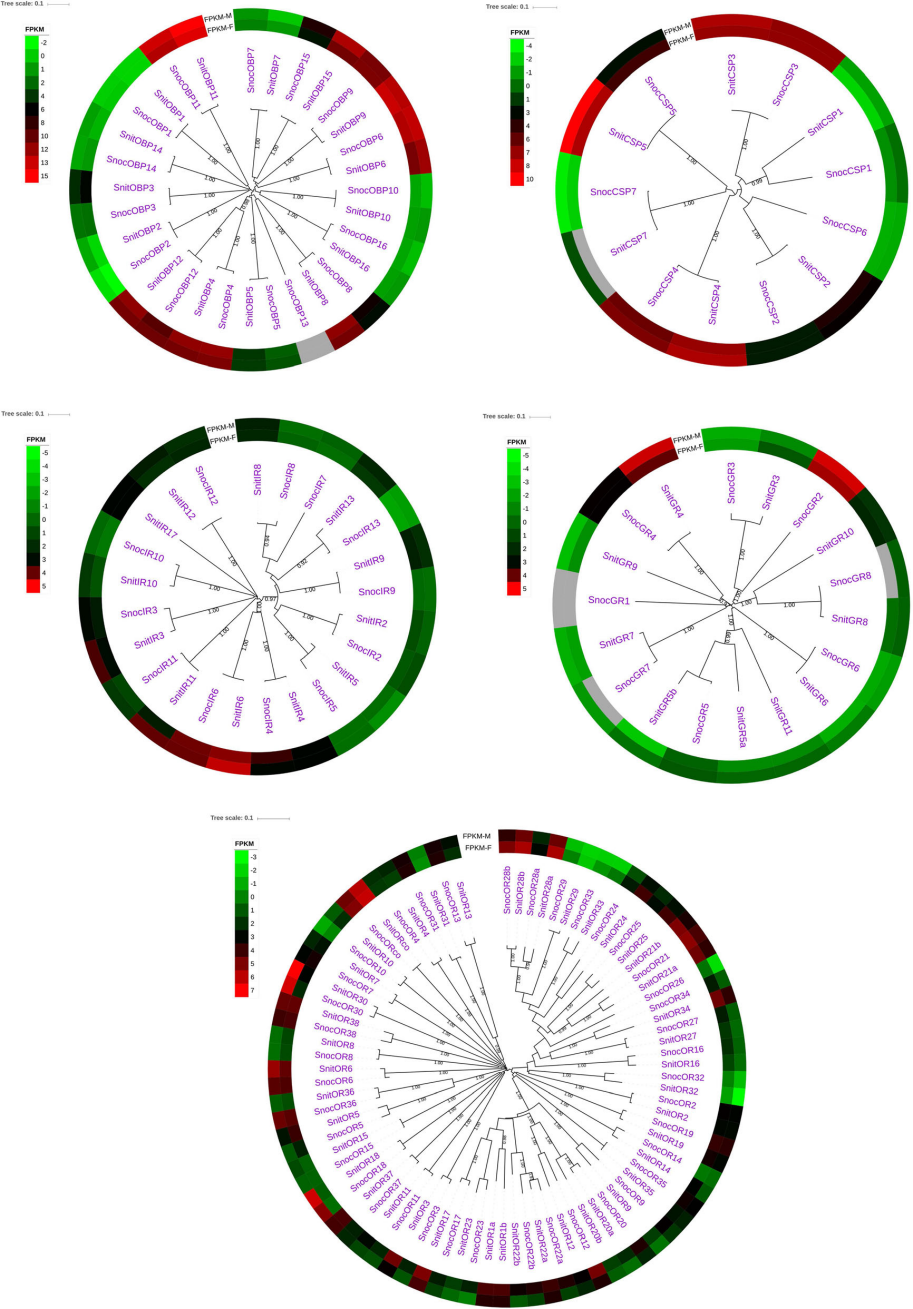
Phylogenetic trees of olfactory genes (OBPs, CSPs, ORs, GRs, and IRs) from *S. noctilio* and *S. notobei*. Homologous genes of the two species cluster together. Expression levels (FPKM) of olfactory genes in male and female woodwasp antennae are displayed behind the gene names. Expression values (FPKM) are shown on a log_2_ scale

We used the olfactory genes of the two woodwasp species to build multiple phylogenetic trees (Fig. 9). In our phylogenetic tree, most of the *S. noctilio* and *S. nitobei* olfactory genes clustered together. Additionally, we found that most of the *S. noctilio* and *S. nitobei* olfactory genes were homologous, supporting the close relationship between the two species. We also found that there were some species-specific genes such as *SnocOBP13*, *SnocCSP6*, *SnocOR26, SnocGR2*, *SnocIR7* in *S. noctilio* and *SnitGR9, SnitGR11, SnitIR17* in *S. nitobei.*

According to the analysis of heat map (Fig. 9) and significant expression of FPKM (Additional file 5), some homologous genes, including *OBP3, OBP8, OBP9, OBP15, CSP2, ORco, OR1, OR13, OR17, OR31, OR34, OR36, OR37, GR4, IR9*, *IR10* and *IR13*, have different expression profiles between two siricids. Among them, *OR13*, *OR31* and *OR36* were highly expressed in *S. noctilio* and other olfactory genes were highly expressed in *S. nitobei* (Additional file 5). In the same way, some of the homologous genes were expressed differently between males and females, such as *OR18* and *OR30*, were both significantly expressed in male antennae of two siricids (Additional file 5).

### Fluorescent quantitative real-time PCR

**Fig. 10:**
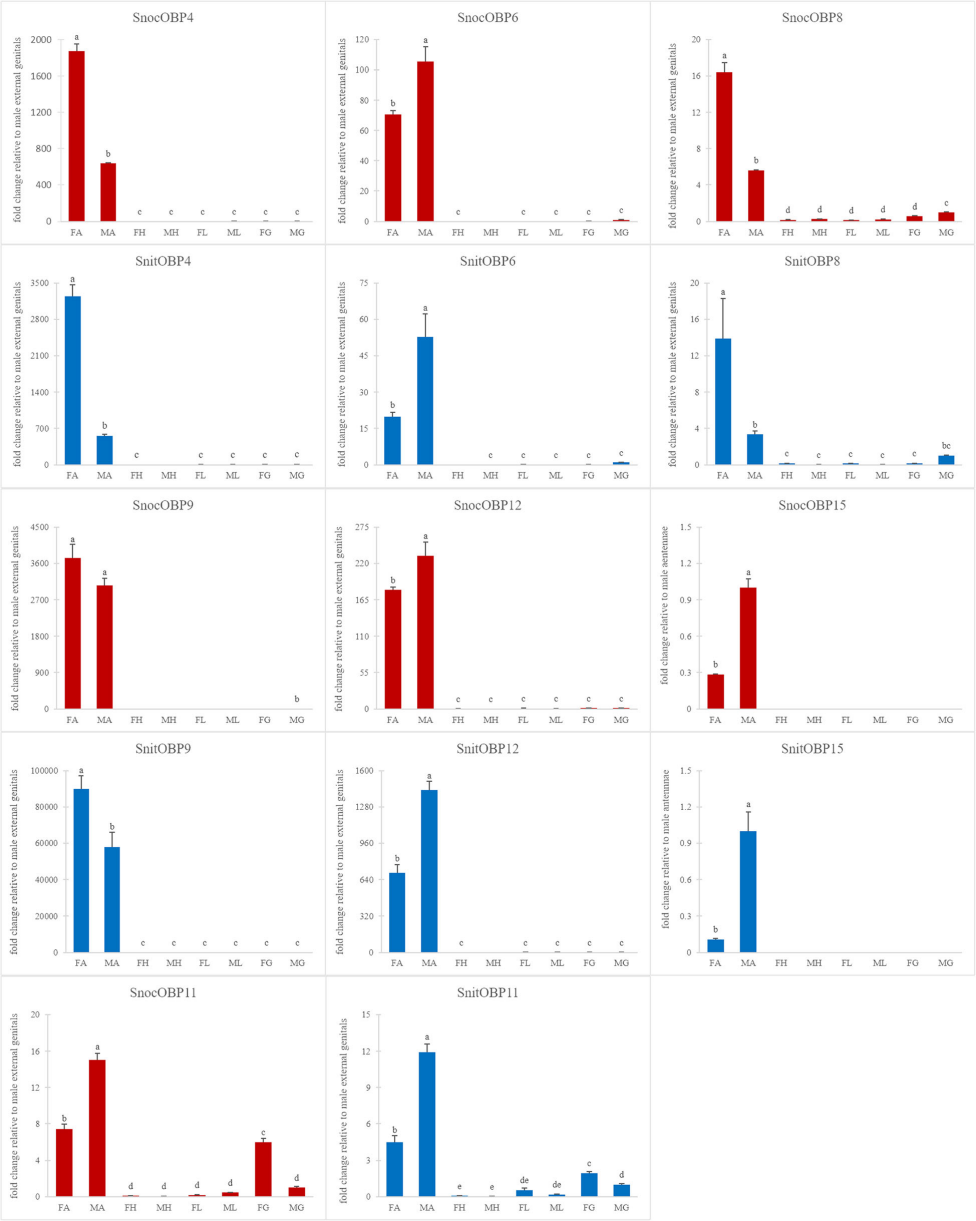
Odorant binding protein (OBP) transcript levels with antennae-biased expression in 4 tissues of male and female *Sirex*. *S. noctilio* data is colored red and *S. nitobei* data is colored blue. The expression level of the male genitalia was set to 1 in order to obtain the relative expression in each tissue. The expression of *SnitOBP15* in male antennae was set to 1 because the expression of male genitalia was too low to display

**Fig. 11:**
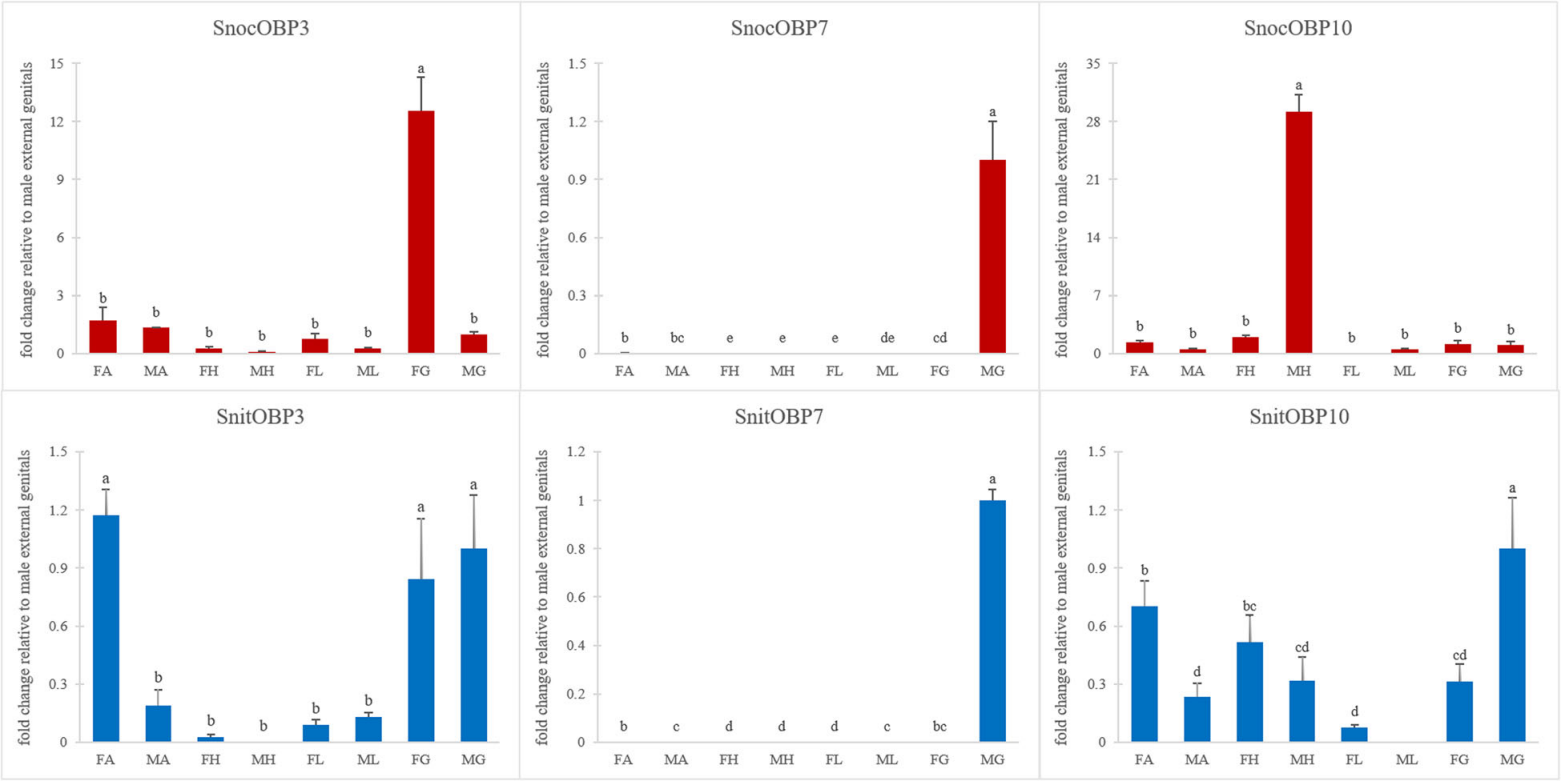
Odorant binding protein (OBP) transcript levels with head-biased, genital-biased or unbiased expression in 4 tissues of male and female siricids. *S. noctilio* data is colored red and *S. nitobei* data is colored blue. The expression level in male genitalia was set to 1 in order to display the relative expression in each tissue

To verify OBPs expression in the antennae and characterize the expression profiles of OBPs in 4 chemosensory tissues (antennae, legs, heads, and externalia), 10 *SnocOBP*s and 10 *SnitOBP*s with high FPKM values were selected for fluorescent quantitative real-time PCR (Figs. 10 and 11). Primers for OBPs and for an internal reference gene (*β-tubulin*) were listed in the Additional file 6.

We compared the results of FPKM value and RT-qPCR, and found that most OBPs expression trends were the same in male and female antennae, further proving the accuracy of transcriptome data. Most OBPs were expressed mainly in the antennae of the two woodwasps. The observed high expression in the antennae suggested that the OBPs may play a role in binding and transporting odor signals in antennae. In addition, species-specific, tissue or sex-biased expression were also observed as follow.

Firstly, significant species-specific expression was observed for many OBP genes, especially those not greatly expressed in the antennae, including *OBP3* and *OBP10. SnocOBP3* was primarily expressed in the genitalia of female *S. noctilio*. *SnocOBP10* was mainly expressed in male heads, while *SnitOBP3* and *SnitOBP10* did not show obvious tissue bias due to low expression levels (Fig. 11). In addition, we also found that some homologous genes mainly expressed in the antennae differ in their expression profiles between the two species. For example, *SnocOBP9* showed a weak expression bias between two sexes but the difference was not significant (*P* > 0.05), however, *SnitOBP9* had a significantly differential expression between two sexes (*P* < 0.05), which had a higher expression in female antennae. The differential expression of these homologous genes may suggest that they strengthened or lost their original function during species differentiation, resulting in olfactory differences between the two species.

Secondly, significant tissue-biased expression was observed for many OBP genes. Most OBPs*,* including *OBP4, OBP6, OBP8, OBP9, OBP12 and OBP15,* were primarily expressed in antennae of two siricids (Fig. 10). For both *SnocOBP9* and *SnitOBP9* that were identified as *PBP* homologues, we observed high expression in antennae and no detectible expression in the other organs. Additionally, *SnocOBP11* and *SnitOBP11* were more highly expressed in the female external genitalia and male antennae when compared to the male external genitalia and female antennae (Fig. 10). High expression of *SnocOBP7* and *SnitOBP7* was detected in male externalia (Fig. 11).

Finally, significant sex-biased expression was observed for many OBP genes, including almost all 10 *SnocOBP*s and 10 *SnitOBP*s with high FPKM values, which were sex-biased expression either in antennae or external genitalia (Figs. 10 and 11)*.* This sex bias may denote different functions of these OBPs between males and females, such as the perception of the opposite sex or oviposition behavior.

## Discussion

### Comparison of olfactory genes in two siricids

Temporal and spatial niches divergence led to no apparent competitive pressures between the two species on *P. sylvestris* var. *mongolica* Litv. in China [14]. In recent study in 2019, we found *S. noctilio* and *S. nitobei* can share similar attractants (α-pinene, β-pinene, 3-carene, camphene) in the field, consistent with that they recognized common host plants [19]. From our transcriptome analysis, most of the olfactory genes identified in two species were homologous between the two species, supporting their close relationship.

On the other hand, trapping experiment also showed although these attractants were attractive to both species, but with different efficiency with each other*.* It is possible that chemical cues used by the two woodwasps are different to some extend. From our transcriptome analysis, some species-specific olfactory genes were identified from the antennal transcriptomes, including *SnocOBP13*, *SnocCSP6*, *SnocOR26, SnocGR2*, *SnocIR7* in *S. noctilio* and *SnitGR9, SnitGR11, SnitIR17* in *S. nitobei.* In addition, some homologous genes, including *OBP3, OBP8, OBP9, OBP15, CSP2, ORco, OR1, OR13, OR17, OR31, OR34, OR36, OR37, GR4, IR9*, *IR10* and *IR13*, were significantly different between the two siricids. Species-bias expression in olfactory genes between the two species will be a study emphasis in further research.

By studying these olfactory genes, the molecular mechanism of the olfactory difference between the two species can be better elucidated.

### Number of OBPs varies greatly among different species

Compared with OBPs of model species, the numbers of OBPs encoded in the genomes of siricids (16 in *S. noctilio* and 15 in *S. nitobei*) were less than those in *D. melanogaster* (51 OBPs), *Anopheles gambiae* (57 OBPs), *B. mori* (44 OBPs), and *N. vitripennis* (82 OBPs) [37–42]. In Hymenoptera, there are species with more OBPs than the siricids, such as those of *Aenasius bambawalei* (54 OBPs), while there are also species with fewer OBPs than the siricids, such as *Osmia cornuta* (6 OBPs) and *Microplitis mediator* (7 OBPs) [43–46]. *A. mellifera* possesses a similar set of these genes, consisting of only 21 OBPs. This suggests differences in the modalities of olfactory discrimination for different insect species.

One possibility is that insects with less OBPs has lower discrimination capabilities than other insects with more OBPs. Some researchers suggest that more singular and closed environments are associated with less lineage-specific OBP expansion such as 7 OBPs of *Ceratosolen solmsi* and 5 OBPs of *Pediculus humanus humanus* [45, 47]. This may also be a reason why the number of OBPs in the sirisids is small. *S. noctilio* and *S. nitobei* live in pure forests or mixed coniferous forests and thus are more likely to receive plant volatiles or interspecies pheromone substances from a closed environment.

In *S. noctilio* and *S. nitobei*, most OBPs are orthologous, supporting the previous reports that the evolution of OBPs is mainly driven by lineage-specific amplification, with few distinct homologues in non-relative species [37, 38, 48]. The siricid OBPs also display strong homology with other Hymenopteran species and have low similarity with OBPs in other insects such as *D. melanogaster* and *B. mori*, further illustrating the evolutionary relationship of OBPs in insect specialization.

### Special subfamilies of OBPs

We did not uncover any Plus-C OBPs in the transcriptomes of *S. noctilio* and *S. nitobei*. Plus-C OBPs were found in Lepidoptera (*B. mori*), Diptera (*D. melanogaster*), Coleoptera (*Anoplophora chinensis*), but were not found in existing Hymenoptera genomes or transcriptomes [49]. For example, Plus-C OBPs were not found in the genomes of Hymenoptera such as *A. mellifera* and *N. vitripennis* [42]. This finding suggests that the Plus-C subtype is rare or even absent in Hymenoptera and has had no influence on the olfactory recognition in Hymenoptera. However, the Minus-C subtype has been widely observed in Hymenopterans. For example, *AmelOBP14–21* and *NvitOBP27, − 38, − 56, and − 58–62* are Minus-C OBPs. We identified one Minus-C OBP in each *S. noctilio* and *S. nitobei*. These two Minus-C OBPs were not homologous with the Minus-C OBPs of *A. mellifera* and *N. vitripennis*, but were homologous to a Classic OBP of *Meteorus pulchricornis*, *MpulOBP1* [50]. It is reasonable that the minus-C OBPs of the wasps could share a common evolutionary ancestor of a classic OBPs as speculated previously in *A. mellifera* that the eight *A. mellifera* minus-C OBPs and *AmelOBP13* shared a common evolutionary ancestor [39]. It can be inferred that the Minus-C OBP originated from a classic OBP that lost two cysteine residues during evolution, rather than having evolved from a Minus-C OBP of another species. The expression values of the two Minus-C OBPs in the *Sirex* transcriptomes were extremely high, indicating that these Minus-C OBPs may be specialized into special OBPs that play a key role in the wasp’s olfactory systems. However, the functions of Plus-C and Minus-C OBPs in insects are not known and need further studies.

### Different expression pattern of OBPs in two siricids

Between two closely related woodwasp species, most genes were found to be orthologous and displayed high sequence similarity (> 90%) and thus may be related to recognition of common host plants. Interestingly, difference in homologous genes between the two species and between the sexes were also found. *SnocOBP3* and *SnocOBP10* was highly expressed in female genitalia and male heads in *S. noctilio* respectively, while *SnitOBP3* and *SnitOBP10* did not show obvious tissue bias in *S. nitobei* due to low expression levels. *SnocOBP9* and *SnitOBP9*, which are *PBP* homologues, were mainly expressed in antennae and thus could be speculated to play a key role in identification of pheromone components. The high expression in both sexes of *SnocOBP9* suggested that the protein is used by both sexes to sense sex pheromones or aggregation pheromones. In contrast, *SnitOBP9* had a significantly differential expression between the two sexes, which have a higher expression in female antennae, which suggested it is more important for *S. nitobei* females to sense male pheromones. Taken together, different molecular mechanisms between the two closely-related species, and the different roles that the OBPs play between the two sexes are indicated.

### Expression pattern of non-antenna specific OBP

OBPs in the antenna play critical roles in the adaptation of insects to a wide variety of environments and life styles. One of these roles is olfaction, a central aspect of insect life, for example, recognizing sex pheromones or plant volatile components, and guiding normal behaviors such as feeding, mating, or laying egg [39].

Another interesting aspect of OBP research is the study of those members found in other sensory organs in addition to antennae. For example, OBPs and CSPs are identified in structures, such as pheromone glands and reproductive organs, where semiochemicals are delivered. The mandibular glands, in *A. mellifera*, known to be the site of synthesis and delivery of several pheromones, expressed a variety of OBPs in a caste and age dependent fashion [51]. In the honey bee only nine OBPs are antenna-specific, and the remaining genes are expressed either ubiquitously or are tightly regulated in specialized tissues or during development. In the mosquito *Aedes aegypti,* OBP22 is expressed in antennae and in male reproductive organs, and is transferred to females during mating [52, 53]. In some moths, CSP has been reported in pheromone glands [54, 55]. In our study, many OBP genes showed significantly tissue-biased expression, including in antennae, the female genitalia (*SnocOBP3, SnocOBP11* and *SnitOBP11*) or male external genitalia (*SnocOBP7 and SnitOBP7*), and male heads *(SnocOBP10*). In a previous report, four *OBP*s and five *CSP*s were found in the venom gland proteome of *S. noctilio* [56]. Through sequence alignment, it was found that the *OBP*s identified in the venom gland were *SnocOBP2*, *SnocOBP6*, *SnocOBP9*, and *SnocOBP11*, and the *CSP* identified was *SnocCSP2–5*, indicating that these *OBP*s and *CSP*s may play special roles in transport or recognition of chemical signals in venom glands [56]. *CSP5* could be a venom gland specific since it was not detected in our antennal transcriptomes. An important task in the subsequent research is to understand the other nonolfactory functions of these olfactory proteins.

### Olfactory genes located on close loci

Among the olfactory genes identified in the transcriptomes, we observed that some of the same types of olfactory genes were located on the close loci, such as *SnocOBP10* and *SnocOBP16*, and *SnitCSP3* and *SnitCSP4*. The distances between the two close loci were only 1000 bp. In previous studies, it was found that olfactory gene families expand through gene duplication and subsequent evolution [57]. The close loci suggest that these genes share a common ancestor gene and supports the gene duplication model of olfactory gene family expansion.

### Number of chemosensory receptor genes varies greatly among different species

ORco has been observed in Diptera, Lepidoptera, Coleoptera, and Hymenoptera. Compared to traditional odor receptors, ORco is highly conserved, and its homology among insects can reach 50–99%. Amino acid sequence analysis revealed a highly conserved region at the end of the ORco sequence [31]. In our phylogenetic analysis, we found two genes, *SnocORco* and *SnitORco*, that were clustered with the honey bee ORco *AmelOR2* [32] and other Hymenoptera ORco, suggesting they could function as a complex with other ORs in the woodwasps as the ORcos in other insects.

At present, in Hymenoptera, most research on chemosensory receptor has focused on the Apocrita, such as the honey bee, *A. mellifera*, *N. vitripennis*, *M. pulchricornis*, *M. mediator, Macrocentrus cingulum*, *Campoletis chlorideae*, and ants [50, 58–60]. However, few studies have focused on chemosensation in Symphyta species, only one study identified receptors in wheat stem sawfly [61]. We analyzed a total of 62 and 69 chemosensory genes in *S. noctilio* and in *S. nitobei*, respectively, including 41 ORs in *S. noctilio* and 43 ORs in *S. nitobei*. The numbers of ORs identified in *S. noctilio* and *S. nitobei* were less than those in most Hymenopteran species, such as *M. mediator* (60) [36], *C. cinctus* (72) [61], *Chouioia cunea* (80) [62], *A. mellifera* (174) [32], *M. pulchricornis* (99) [50], *N. vitripennis* (301) [63], *M. cingulum* (109) [64], and *C. chlorideae* (211) [60]. However, the numbers of ORs identified in *S. noctilio* and *S. nitobei* were greater than that of some Coleopteran species, such as *Anoplophora glabripennis* (37), *Agrilus planipennis* (2), and *Monochamus alternatus* (9) [65–67]. IRs are a relatively ancestral and conserved receptor family [34, 35]. We identified 13 and 16 IR transcripts in *S. noctilio* and *S. nitobei*, respectively, similar to the number in *M. cingulum* (13) [64] and *C. cunea* (10) [62], but less than those in Dipterans, such as *D. melanogaster* (66) [68, 69]. Our phylogenetic analysis revealed that SnocIR6 and SnitIR6 cluster into a highly conserved IR25a subfamily, and SnocIR4 and SnitIR4 belong to the IR8a family. Therefore, we speculate that IR6 and IR4 are co-receptors of the woodwasps. Similarly, two IRco genes were found in *M. mediator* [36], while only one IR25a homolog was found in the woodwasp species *C. cunea* and *M. pulchricornis.* And we identified a total of 18 GRs (8 in *S. noctilio* and 10 in *S. nitobei*), which is similar to the number identified in the honey bee but significantly fewer than those in *C. cinctus* (35), *N. vitripennis* (58), and *D. melanogaster* (68) [61, 63, 70].

There are many possible reasons for the differences in olfactory gene number, including sequencing depth. In addition, the physiological state of the woodwasps during sampling may affect gene expression [36, 71]. In addition, we only evaluated antennal transcription, and genes with low expression in other tissues may not be detected. It may also be related to the relative simpler chemical environment and biological habits of Symphyta species than those of social insects and parasitoids [32, 72].

### Characteristics and function of chemosensory receptors with biased expression

ORs with sex-biased expression in siricids was found. Most of antennae-enriched ORs were significantly female-biased and are possibly related to some female-specific behavior, such as identification of oviposition sites and detection of oviposition substances. However, there are four OR genes (*SnocOR18*, S*nocOR30*, *SnitOR18*, *and SnitOR30*) were found to be significantly male-biased and their expression levels in the antennae display 10-fold differences between the sexes (Additional file 5), which are possibly related to recognition of pheromones, such as the aggregation pheromone of *S. noctilio* reported [18].

It is worth noting that the expression patterns of most *SnocOR30* and *SnitOR30* display high homology with *AmelOR170*. *AmelOR170* has shown a biased expression pattern in drone antennae, but the receptor does not bind 9-oxo-2-decenoic acid [73]. Furthermore, none of the investigated woodwasp genes was clustered with *AmelOR11*, which is the receptor for 9-oxo-2-decenoic acid (9-ODA), the main component of the queen retinue pheromone (QRP) in the honey bee. This result confirms that the olfaction proteins are species specific and different among Hymenoptera due to their wide ecological variations in environment. *MmedOR9*, which is homologous to *SnocOR18* and *SnitOR18*, is highly and specifically expressed in males, suggesting that these genes may play an important role in males such as receiving sex pheromones in Hymenoptera [74].

Expression of trehalose receptors in siricids indicated 2 and 7 sweet receptors in the transcriptomes of *S. noctilio* and *S. nitobei*, respectively. Most of these sweet receptors were found to be trehalose receptors. Trehalose is a non-reducing sugar composed of two α-glucose molecules joined by an 1,1-glycosidic bond. The sugar is chemically stable and protects plants, plant cells, and plant proteins from freezing and drying. It is a stress-resistant protection mechanism. Trehalose is also present in the body fluid of insects and can be used as a energy source for flying. As an important blood sugar in insects, trehalose is present in almost all tissues and organs of insects [75]. Trehalose can influence insects’ choice for food via recognition by GRs. Thus, trehalose receptors are important for vital biological processes in insects.

## Conclusions

By examining their antennal transcriptomes, we analyzed 86 and 91 olfactory genes from *S. noctilio* and *S. nitobei*, respectively. The number of olfactory genes varies among different species, which may be related to possible reasons, including different olfactory discrimination capabilities based on individual chemical environment and biological habits for different insect species, sequencing depth and sampling status. Most of the olfactory genes identified in two species were homologous between the two species. However, some species-specific olfactory genes were identified from the antennal transcriptomes, which indicated different olfactory molecular mechanisms between the two closely-related species.

Additionally, we verified the expression of 10 *SnocOBP*s and 10 *SnitOBP*s in male and female antennae, respectively, thereby confirming the accuracy of our transcriptome data. The high expression of OBPs in antennae supports the function of OBPs in the semiochemical recognition process. Significant tissue-biased or sex-biased expression was observed for many OBP genes, especially those not greatly expressed in the antennae, which suggest OBPs are involved in activities of daily living, for example, recognizing sex pheromones or plant volatile components, and guiding normal behaviors such as feeding, mating, or oviposition. Species-specific expression for several OBP genes may suggest that they strengthened or lost their original function during species reproductive isolation, resulting in olfactory differences between the two species. The olfactory proteins of the siricids were obtained through antennal transcriptome sequencing. However, in order to explore the olfactory mechanisms of the siricids, it is necessary to perform protein expression, fluorescence binding competition, and molecular docking studies.

## Methods

### Sample collection and preservation

*S. noctilio* adults emerge in early July over a period of 2 months and *S. nitobei* adults emerge in early September, but adult woodwasps only live for 5–12 days [4, 14]. The *S. noctilio* and *S. nitobei* woodwasps used in these experiments were collected from Tongliao, Inner Mongolia. Injured wood that exhibited premature aging and teardrop-like flow gum points were selected. The selected woods were cut into 1-m long sections and placed in a net cage while waiting for the wasps to emerge. The wasps were caught immediately after eclosion and time, status, and sex information were recorded. We separated the antennae from the adults and put them in RNA later buffer solution (Invitrogen, Carlsbad, CA, USA). The antennae were stored at 4 °C for 24 h and then stored at − 20 °C or at − 80 °C for long-term storage at the Forest Conservation Laboratory.

### RNA extraction and Illumina transcriptome sequencing

Total RNA was extracted from adult antennae of both sexes separately (20 antennae each from males and females) using the QIAGEN RNeasy Mini Kit (No. 74134; Qiagen, Hilden, Germany) following the manufacturer’s instructions. The RNA concentration was quantified using a NanoDrop 8000 spectrophotometer (Thermo, Waltham, MA, USA). RNA quality was verified using a 2100 Bioanalyzer RNA Nanochip (Agilent, Santa Clara, CA, USA). The high-quality RNA samples (OD260/280 = 1.8–2.2, OD260/230 ≥ 2.0, RIN ≥ 6.5, 28S:18S ≥ 1.0, > 10 μg) were placed at − 80 °C and used to generate cDNA libraries.

The cDNA library construction and Illumina sequencing on the HiSeq 4000 platform were performed at Majorbio Corporation (Shanghai, China). RNA-seq transcriptome libraries were prepared using 5 μg of total RNA using the TruSeqTM RNA sample preparation kit from Illumina (San Diego, CA, USA). First, messenger RNA (mRNA) was isolated by oligo (dT) beads according to the poly-A selection, then the mRNA was fragmented to about 300 bp length using fragmentation buffer. Second, the mRNA fragments were reversed to synthesize one-stranded cDNA and double-stranded cDNA using the SuperScript double-stranded cDNA synthesis kit (Invitrogen) with random hexamer primers (Illumina). Next, according to the Illumina library construction plan, the synthesized cDNA was subjected to end-repair, phosphorylation, and ‘A’ base addition. PCR amplification was performed for 15 cycles using Phusion DNA polymerase (New England Biolabs, NEB) for cDNA library enrichment. The cDNA target fragments of 200–300 bp were selected using Certified Low Range Ultra Agarose (Bio-Rad) from cDNA library. The cDNA library after fragment selection process was quantified using a TBS-380 mini fluorometer, and was subjected to generate clusters by bridge PCR amplification according to quantitative proportions. Finally the cDNA library was sequenced using an Illumina HiSeq 4000 to generate 150 bp paired-end reads.

### Assembly and function annotation

Trimming and quality control of the raw paired-end reads was performed using SeqPrep (https://github.com/jstjohn/SeqPrep) and Sickle (https://github.com/najoshi/sickle) to generate clean, high-quality reads. De novo transcriptome assembly was carried out with the short read assembly program Trinity (http://trinityrnaseq.sourceforge.net/) [76]. The consensus cluster sequences and singletons comprise the UniGene dataset. The annotation of unigenes was performed by NCBI BLASTX against a combined database of non-redundant (Nr) String, KEGG, and SwissProt protein sequences with an E-value threshold <1e^− 5^. The BLAST results were then processed with the Blast2GO program (http://www.blast2go.com/b2ghome) for gene ontology (GO) annotation, which described the function of unigenes, such as their biological processes, molecular functions, and cellular components [77, 78]. The longest ORF for each unigene was determined by the NCBI ORF Finder tool (http://www.ncbi.nlm.nih.gov/gorf/gorf.html). Expression levels are displayed as FPKM values (fragments per kilobase per million reads), which were calculated by RSEM (RNA-Seq by Expectation-Maximization) v1.2.6.

### Identification of chemosensory-related genes

The annotation of olfactory unigenes (OBP, CSP, OR, GR, IR, and SNMP) was performed by searching the Nr protein database through NCBI BLASTX, with an E-value threshold <1e-5. The available protein sequences from Insecta species were used to identify candidate olfaction unigenes in *S. noctilio* and *S. nitobei*. Signal P4.0 (http://www.cbs.dtu.dk/services/SignalP/) was used to search for the presence of N-terminal signal peptides in candidate OBP and PBP, and TMHMM server v3.0 (http://www.dk/services/TMHMM/) was used to predict the transmembrane domains of candidate OR, IR, GR and SNMP. After performed amino acid sequence alignment using the Muscle method, phylogenetic trees of olfactory unigenes were constructed using the neighbor-joining (NJ) method with the P-distances model and pairwise deletion of gaps in the MEGA v6.0 software package. The sequences used to build the phylogenetic trees included the olfactory genes from *S. noctilio* and *S. nitobei* transcriptomes, and other olfactory genes from other insects by NCBI (Additional file 7). The reliability of the tree structure and node support was evaluated through an analysis of bootstrap with 1000 replicates. Only bootstrap values ≥0.5 were shown at the corresponding nodes. The lineages with bootstrap values ≥0.9 were labelled with red spots, and bootstrap values between 0.7 and 0.9 were marked with blue spots. The phylogenetic trees were modified with ITOL (https://itol.embl.de/).

### Expression analysis by quantitative real-time PCR

The expression of candidate chemosensory genes was performed using fluorescent quantitative real-time PCR (RT-qPCR). RT-qPCR experiments in *S. noctilio* and *S. nitobei* were performed separately. Antennae, legs (including the propodium, mesopodium, and metapedes), heads, and external genitals were collected from ten male or ten female adult woodwasps for each biological replicates. Three biological replicates and three technical replicates were used with each qPCR reaction for each tissue to examine reproducibility. Total RNA was extracted following the methods described above and used as template for cDNA synthesis using the PrimeScript RT Reagent Kit with gDNA Eraser (No. RR047A; Takara, Shiga, Japan). Gene-specific primers of the chemosensory genes were designed using Primer3Plus (http://www.primer3plus.com/cgi-bin/dev/primer3plus.cgi) (see Additional file 3).

In order to the accuracy of RT-qPCR, we selected six genes as reference genes from the *S.noctilio* antennal transcriptome (Actin1, Actin2, Alpha-Tubulin, Beta-Tubulin, TATA Binding protein TBP, Succinate dehydrogenase SDHA). The primers of candidate genes were designed in the same way as chemosensory genes, and the candidate genes were evaluated by GeNorm and Normfinder. GeNorm results indicated that the minimum number of β-tubulin is within the allowable range, and average expression stability M values of β-tubulin were less than 0.5 (Additional file 8, Figure S1-S4). Normfinder results showed that Beta-tubulin was the best reference gene in *S. noctilio* and *S. nitobei*, which were 0.136 and 0.095, respectively (Additional file 8, Table S1, S2). These results indicated that Beta-tubulin is a reliable reference gene for both *S. noctilio* and *S. nitobei* for RT-qPCR. Primers for the internal reference gene (*β-tubulin*) were listed in the Additional file 6.

PCR analysis was conducted using the Bio-Rad CFX Connect PCR System (Hercules, CA, USA). Each 25 μl PCR reaction contained 12.5 μl of SYBR Premix Ex Taq II (No. RR820A; Takara), 2 μl of sample cDNA, 1 μl of forward primer (10 mM), 1 μl of reverse primer (10 mM) and 8.5 μl of dH2O (sterile distilled water). The RT-qPCR cycling parameters were as follows: 95 °C for 30 s; then 40 cycles of 95 °C for 5 s and 60 °C for 30 s; followed by 65 °C to 95 °C in increments of 0.5 °C for 5 s. Each experiment included negative controls without template to maintain experimental accuracy. Bio-Rad CFX Manager (version 3.1.1517.0823) was used to normalize expression based on ΔΔCT values, with Beta-Tubulin as reference gene and the male external genitals samples as control samples. The 2^−ΔΔ^ CT method was used to generate relative expression values. The comparative analyses for each chemosensory gene among eight tissue samples were assessed using a one-way nested analysis of variance (ANOVA) and the significance analysis was performed with Tukey’s honestly significance difference tests in SPSS 21.0.

## Supplementary Information

**Additional file 1: **Transcriptome information of *S. noctilio* and *S. nitobei*. **Table S1.** Summary of raw reads obtained from *S. noctilio* and *S. nitobei* antennal transcriptomes. **Table S2.** Summary of clean reads obtained from *S. noctilio* and *S. nitobei* antennal transcriptomes. **Table S3.** Assembly statistics for the *S. noctilio* antennal transcriptome. **Table S4.** Assembly statistics for the *S. nitobei* antennal transcriptome.

**Additional file 2: **Best blastX hits for putative odorant binding proteins (OBPs), chemosensory proteins (CSPs), odorant receptors (ORs), sensory neuron membrane proteins (SNMPs), ionotropic receptors (IRs), and gustatory receptors (GRs) of *S. noctilio* and *S. nitobei*. (Table S1, Table S2, Table S3, Table S4, Table S5 and Table S6). **Table S1.** Sequence information and best blasts match information of odorant binding proteins (OBPs). **Table S2.** Sequence information and best blasts match information of chemosensory proteins (CSPs). **Table S3.** Sequence information and best blasts match information of odorant receptors (ORs). **Table S4.** Sequence information and best blasts match information of sensory neuron membrane proteins (SNMPs). **Table S5.** Sequence information and best blasts match information of gustatory receptors (GRs). **Table S6.** Sequence information and best blasts match information of ionotropic receptors (IRs).

**Additional file 3.** The sequence of OBPs with alignment were shown six conserved cysteine residues.

**Additional file 4.** The sequence of CSPs with alignment were shown four conserved cysteine residues.

**Additional file 5. **Significance test of The FPKM value of homologous genes (OBPs, CSPs, ORs, SNMPs, GRs and IRs) between two siricid species and male and female. *S. noctilio* data is colored red and *S. nitobei* data is colored blue.

**Additional file 6.** Primers of odorant binding proteins and reference gene for quantitative real-time PCR.

**Additional file 7.** The OBPs, CSPs, ORs, SNMPs, GRs and IRs of Hymenoptera, Diptera, Lepidoptera, Coleoptera, Blattaria and Orthoptera used in building neighbor-joining phylogenetic trees.

**Additional file 8: **Selection of reference gene in *S. noctilio* and *S. nitobei*. **Table S1.** Normfinder result of the six reference genes in *S. noctilio*. **Table S2.** Normfinder result of the six reference genes in *S. nitobei*. **Figure S1.** Average expression stability values of remaining control genes in *S. noctilio*. **Figure S2.** Determination of the optimal number of control genes for normalization in *S. noctilio*. **Figure S3.** Average expression stability values of remaining control genes in *S. nitobei*. **Figure S4.** Determination of the optimal number of control genes for normalization in *S. nitobei.*

## Data Availability

All supporting data is included within the article and its additional files. And the transcriptome data were submitted to NCBI, the accession number of *S. noctilio* are from SAMN11338151 to SAMN11338156 and the accession number of *S. nitobei* are from SAMN11338569 to SAMN11338574. All of the olfactory protein gene sequences were submitted to Genbank, accession number are MK425751- MK425766, MK674426- MK674453 and MK748989- MK749121.

## Abbreviations

OSNs: Olfactory sensory neurons
GO: Gene ontology
ORFs: Open reading frame
FPKM: Fragments per kilobase per million reads
NJ: Neighbor-joining
RT-qPCR: Fluorescent quantitative real-time PCR
OBPs: Odorant binding proteins
CSPs: Chemosensory proteins
ORs: Odorant receptors
ORco: Odorant receptor co-receptor
SNMPs: Sensory neuron membrane proteins
GRs: Gustatory receptors
IRs: Ionotropic receptors
ODEs: Odorant-degrading enzymes
