# Supplementary material for: Antennal transcriptome analysis of olfactory genes and characterizations of odorant binding proteins in two woodwasps, Sirex noctilio and Sirex nitobei (Hymenoptera: Siricidae)
Source: BMC Genomics. 2021 Mar 10;22:172. doi: 10.1186/s12864-021-07452-1 (PMC7945326; doi:10.1186/s12864-021-07452-1)
Supplement: Supplementary file 1 — Additional file 1: Transcriptome information of S. noctilio and S. nitobei. Table S1. Summary of raw reads obtained from S. noctilio and S. nitobei antennal transcriptomes. Table S2. Summary of clean reads obtained from S. noctilio and S. nitobei antennal transcriptomes. Table S3. Assembly statistics for the S. noctilio antennal transcriptome. Table S4. Assembly statistics for the S. nitobei antennal transcriptome. [file 12864_2021_7452_MOESM1_ESM.pdf]

Table S1. Summary of raw reads obtained from *S. noctilio* and *S. nitobei* antennal transcriptomes

Sample_ID	Total_Reads	Total_Bases	Error%	Q20%	Q30%	GC%
Snoc 1_F	51044156	7707667556	0.0145	96.76	92.3	42.67
Snoc 2_F	59929292	9049323092	0.0144	96.8	92.36	42.6
Snoc 3_F	57039344	8612940944	0.0142	96.9	92.63	39.02
Snoc 1_M	64496020	9738899020	0.0132	97.44	93.67	41.36
Snoc 2_M	56140478	8477212178	0.0141	96.96	92.64	46.55
Snoc 3_M	53538322	8084286622	0.0146	96.72	92.21	42.87
Snit 1_F	65908130	9952127630	0.0143	96.88	92.51	42.13
Snit 2_F	48942274	7390283374	0.014	96.96	92.79	36.92
Snit 3_F	49483604	7472024204	0.0142	96.87	92.64	36.44
Snit 1_M	54558776	8238375176	0.0146	96.73	92.18	42.19
Snit 2_M	57064726	8616773626	0.0143	96.92	92.57	42.15
Snit 3_M	53771404	8119482004	0.0143	96.87	92.51	41.91

Note: Q20% and Q30% are the percentage of bases which the Phred score was greater than 20 or 30. GC% is GC content percentage.

Table S2. Summary of clean reads obtained from *S. noctilio* and *S. nitobei* antennal transcriptomes

Sample_ID	Total_Reads	Total_Bases	Error%	Q20%	Q30%	GC%
Snoc 1_F	49891070	7371454411	0.0127	98.18	94.49	42.48
Snoc 2_F	58669346	8664078283	0.0127	98.18	94.49	42.41
Snoc 3_F	55887482	8253513321	0.0125	98.25	94.71	38.84
Snoc 1_M	62192700	9219770317	0.0119	98.52	95.37	41.23
Snoc 2_M	54045142	7992360451	0.0125	98.25	94.65	46.39
Snoc 3_M	52337684	7728241956	0.0128	98.16	94.42	42.68
Snit 1_F	64371826	9532004071	0.0126	98.24	94.63	41.98
Snit 2_F	47969434	7087270978	0.0124	98.29	94.83	36.73
Snit 3_F	48482000	7151845379	0.0125	98.26	94.77	36.21
Snit 1_M	53213400	7872680155	0.0128	98.15	94.39	42.03
Snit 2_M	55797468	8257697138	0.0126	98.24	94.62	41.99
Snit 3_M	52505128	7771215028	0.0126	98.25	94.65	41.76

Note: Q20% and Q30% are the percentage of bases which the Phred score was greater than 20 or 30.
GC% is GC content percentage.

Table S3. Assembly statistics for the *S. noctilio* antennal transcriptome

Type	Total sequences	Total sequence (bp)	GC%	Max length (bp)	Min length (bp)	Average length (bp)	N50	N90
Unigene	47253	61586545	40	56024	201	1303.34	2536	609
Transcript	66977	126429451	40.09	56024	201	1887.65	3937	800

Table S4. Assembly statistics for the *S. nitobei* antennal transcriptome

Type	Total sequences	Total sequence (bp)	GC%	Max length (bp)	Min length (bp)	Average length (bp)	N50	N90
Unigene	46866	55062400	40.71	39567	201	1174.89	2722	406
Transcript	64055	113931759	40.32	39567	201	1778.66	4181	745
