# Supplementary material for: Antennal transcriptome analysis of olfactory genes and characterizations of odorant binding proteins in two woodwasps, Sirex noctilio and Sirex nitobei (Hymenoptera: Siricidae)
Source: BMC Genomics. 2021 Mar 10;22:172. doi: 10.1186/s12864-021-07452-1 (PMC7945326; doi:10.1186/s12864-021-07452-1)
Supplement: Supplementary file 2 — Additional file 2: Best blastX hits for putative odorant binding proteins (OBPs), chemosensory proteins (CSPs), odorant receptors (ORs), sensory neuron membrane proteins (SNMPs), ionotropic receptors (IRs), and gustatory receptors (GRs) of S. noctilio and S. nitobei. (Table S1, Table S2, Table S3, Table S4, Table S5 and Table S6). Table S1. Sequence information and best blasts match information of odorant binding proteins (OBPs). Table S2. Sequence information and best blasts match information of chemosensory proteins (CSPs). Table S3. Sequence information and best blasts match information of odorant receptors (ORs). Table S4. Sequence information and best blasts match information of sensory neuron membrane proteins (SNMPs). Table S5. Sequence information and best blasts match information of gustatory receptors (GRs). Table S6. Sequence information and best blasts match information of ionotropic receptors (IRs). [file 12864_2021_7452_MOESM2_ESM.pdf]

Table S1. Sequence information and best blasts match information of odorant binding proteins (OBPs).

Number	Unigene ID	Transcript ID	Transcript Length（bp）	ORF Length（aa）	Complete ORF	Signal peptide	FPKM		Blastx match					
							F	M	Name	Acc.number	Species	Score	E-value	Identity(%)
SnocOBP1	c34834_g1	c34834_g1_i1	707	173	YES	1_20	0.712	0.632	OBP1	ASL05033.1	*Nilaparvata lugens*	325	5.00E-111	100%
SnocOBP2	c37677_g1	c37677_g1_i1	939	135	YES	NO	0.181	0.398	OBP8	ARN17864.1	*Cephus cinctus*	182	1.00E-53	59%
SnocOBP3	c41939_g1	c41939_g1_i1	754	140	YES	1_18	6.346	5.457	GOBP83a	XP_012281355.1	*Orussus abietinus*	61.2	3.00E-08	27%
SnocOBP4	c43031_g1	c43031_g1_i1	1642	141	YES	1_20	3996.303	2309.888	GOBP72	XP_012274260.1	*Orussus abietinus*	124	1.00E-29	45%
SnocOBP5	c52139_g1	c52139_g1_i1	631	137	YES	1_19	10.167	13.484	OBP3	AKC02194.1	*Oedaleus asiaticus*	66.6	2.00E-10	32%
SnocOBP6	c52489_g1	c52489_g1_i1	1168	137	YES	1_20	8655.034	11966.271	OBP17	AMQ76470.1	*Apolygus lucorum*	109	3.00E-25	44%
SnocOBP7	c58355_g2	c58355_g2_i3	1957	131	YES	YES	4.413	2.648	OBP10	ARN17866.1	*Cephus cinctus*	185	5.00E-52	67%
SnocOBP8	c59491_g17	c59491_g17_i2	4928	115	YES	NO	144.678	78.556	OBP4	AGI05203.1	*Osmia cornuta*	60.5	8.00E-07	27%
SnocOBP9	c60123_g1	c60123_g1_i1	2640	141	YES	1_21	2276.798	2005.290	ASP1	AAD51944.1	*Apis mellifera*	138	3.00E-34	40%
SnocOBP10	c61320_g1	c61320_g1_i5	5328	145	YES	1_18	1.553	0.986	GOBP69a	XP_003398556.1	*Bombus terrestris*	245	3.00E-71	79%
SnocOBP11	c664_g1	c664_g1_i1	1718	133	YES	1_17	8559.988	18477.538	OBP1	AQN78379.1	*Meteorus pulchricornis*	58.2	2.00E-06	32%
SnocOBP12	c95599_g1	c95599_g1_i1	1063	140	YES	1_20	908.174	1494.060	OBP2	ASM47934.1	*Macrocentrus cingulum*	127	3.00E-32	46%
SnocOBP13	c97258_g1	c97258_g1_i1	203	54	NO	NO	0.000	0.000	OBP2	ACI30680.1	*Nilaparvata lugens*	134	4.00E-39	99%
SnocOBP14	c50164_g1	c50164_g1_i2	687	138	YES	1_17	2.303	1.731	GOBP69a	XP_012287798.1	*Orussus abietinus*	90.9	2.00E-19	36%
SnocOBP15	c60675_g17	c60675_g17_i1	7413	134	YES	1_19	65.389	213.434	GOBP83a	XP_015601898.1	*Cephus cinctus*	155	3.00E-44	54%
SnocOBP16	c61320_g1	c61320_g1_i5	5328	144	YES	1_21	1.553	0.986	OBP12	RLZ02154.1	*Cephus cinctus*	122	2.00E-30	47%
SnitOBP1	c1846_g1	c1846_g1_i1	758	173	YES	1_20	0.525	0.527	OBP1	ASL05033.1	*Nilaparvata lugens*	324	2.00E-110	99%
SnitOBP2	c48080_g1	c48080_g1_i1	1042	161	YES	1_22	0.488	1.522	OBP8	ARN17864.1	*Cephus cinctus*	189	8.00E-56	54%
SnitOBP3	c10755_g1	c10755_g1_i1	693	140	YES	1_18	89.042	35.679	GOBP83a	XP_012281356.1	*Orussus abietinus*	61.2	2.00E-08	27%
SnitOBP4	c55509_g2	c55509_g2_i2	6105	141	YES	1_20	3414.188	1867.683	GOBP72	XP_012274260.1	*Orussus abietinus*	125	6.00E-34	46%
SnitOBP5	c12695_g1	c12695_g1_i1	594	137	YES	1_19	27.377	23.854	OBP3	AKC02194.1	*Oedaleus asiaticus*	66.6	2.00E-10	32%
SnitOBP6	c51375_g1	c51375_g1_i1	5404	137	YES	1_20	1941.750	4972.144	OBP17	AMQ76470.1	*Apolygus lucorum*	107	8.00E-24	43%
SnitOBP7	c56521_g1	c56521_g1_i2	912	131	YES	1_22	2.299	0.741	OBP10	ARN17866.1	*Cephus cinctus*	186	1.00E-55	67%
SnitOBP8	c55509_g2	c55509_g2_i2	6105	139	YES	1_19	3414.188	1867.683	OBP4	AGI05203.1	*Osmia cornuta*	68.2	8.00E-12	24%
SnitOBP9	c46404_g2	c46404_g2_i1	990	141	YES	1_21	14770.289	9164.839	ASP1	AAD51944.1	*Apis mellifera*	137	9.00E-39	44%
SnitOBP10	c59240_g5	c59240_g5_i3	7116	145	YES	1_18	1.936	2.296	GOBP69a	XP_003398556.1	*Bombus terrestris*	245	3.00E-71	79%
SnitOBP11	c20656_g3	c20656_g3_i1	646	133	YES	1_17	25171.412	54215.036	OBP1	AQN78379.1	*Meteorus pulchricornis*	60.5	5.00E-08	32%
SnitOBP12	c55509_g2	c55509_g2_i2	6105	146	YES	1_26	3414.188	1867.683	OBP2	ASM47934.1	*Macrocentrus cingulum*	126	3.00E-34	45%
SnitOBP14	c46081_g1	c46081_g1_i1	692	138	YES	1_17	1.160	1.950	GOBP56d	KZC12387.1	*Dufourea novaeangliae*	84.7	5.00E-17	37%
SnitOBP15	c52959_g2	c52959_g2_i1	785	134	YES	1_19	1709.497	7371.440	GOBP83a	XP_015601898.1	*Cephus cinctus*	159	9.00E-46	56%
SnitOBP16	c59240_g5	c59240_g5_i3	7116	144	YES	1_21	1.936	2.296	OBP2	AGZ04921.1	*Laodelphax striatella*	55.8	5.00E-07	28%

Table S2. Sequence information and best blasts match information of chemosensory proteins (CSPs).

Number	Unigene ID	Transcript ID	Transcript Length （bp）	ORF Length （aa）	Complete ORF	Signal peptide	FPKM		Blastx match					
							F	M	Name	Acc.number	Species	Score	E-value	Identity(%)
SnocCSP1	c40184_g1	c40184_g1_i1	535	131	YES	1_17	0.585	1.125	CSP12	AGZ04940.1	*Laodelphax striatella*	222	6.00E-72	91%
SnocCSP2	c57844_g2	c57844_g2_i3	1043	124	YES	1_17	6.151	6.120	CSP3	ALG36156.1	*Sclerodermus sp. MQW-2015*	170	2.00E-47	75%
SnocCSP3	c58129_g2	c58129_g2_i1	1030	138	YES	1_25	164.000	159.828	CSP1	AGZ04911.1	*Sogatella furcifera*	119	2.00E-29	47%
SnocCSP4	c61484_g2	c61484_g2_i1	1348	131	YES	1_18	79.513	118.111	CSP7	ALG36160.1	*Sclerodermus sp. MQW-2015*	177	7.00E-51	61%
SnocCSP5	c9266_g1	c9266_g1_i1	1007	114	YES	1_19	35.163	7.429	CSP5	ALG36158.1	*Sclerodermus sp. MQW-2015*	180	3.00E-53	74%
SnocCSP6	c95675_g1	c95675_g1_i1	470	71	YES	NO	0.316	0.334	CSP9	AGZ04919.1	*Sogatella furcifera*	162	8.00E-49	97%
SnocCSP7	c72059_g1	c72059_g1_i1	462	78	NO	NO	0.168	0.092	CSP4	AGZ04932.1	*Laodelphax striatella*	159	3.00E-47	97%
SnitCSP1	c10722_g1	c10722_g1_i1	384	69	NO	NO	0.146	0.396	CSP1	AGZ04911.1	*Sogatella furcifera*	106	4.00E-27	72%
SnitCSP2	c56120_g2	c56120_g2_i2	2050	124	YES	1_17	18.436	11.745	CSP3	ALG36156.1	*Sclerodermus sp. MQW-2015*	170	1.00E-47	75%
SnitCSP3	c44692_g1	c44692_g1_i1	3280	138	YES	1_25	177.876	275.049	CSP1	ALG36160.1	*Sogatella furcifera*	119	8.00E-32	47%
SnitCSP4	c44692_g1	c44692_g1_i1	3280	131	YES	1_18	177.876	275.049	CSP7	ALG36160.1	*Sclerodermus sp. MQW-2015*	182	1.00E-49	62%
SnitCSP5	c28566_g1	c28566_g1_i1	1013	114	YES	1_19	272.540	1298.647	CSP2	NP_001071278.1	*Apis mellifera*	181	1.00E-53	74%
SnitCSP7	c72249_g1	c72249_g1_i1	236	76	NO	NO	0.000	2.151	CSP4	AGZ04932.1	*Laodelphax striatella*	154	6.00E-47	97%

Table S3. Sequence information and best blasts match information of odorant binding proteins (ORs).

Number	Unigene ID	Transcript ID	Transcript Length (bp)	ORF Length (aa)	Complete ORF	FPKM		Blastx match					
						F	M	Name	Acc.number	Species	Score	E-value	Identity(%)
SnocORco	c104293_g1	c104293_g1_i1	4242	481	YES	47.069	38.235	Orco	XP_012273699.1	*Orussus abietinus*	853	0.00E+00	88%
SnocOR1	c36926_g1	c36926_g1_i3	953	80	NO	1.099	0.463	OR13a	XP_012167490.1	*Bombus terrestris*	107	3.00E-35	38%
SnocOR2	c50984_g1	c50984_g1_i1	1622	388	YES	10.366	9.808	OR82a	XP_003393433.2	*Bombus terrestris*	330	6.00E-105	42%
SnocOR3	c52371_g1	c52371_g1_i1	2840	407	YES	5.686	2.181	OR77	NP_001164671.1	*Nasonia vitripennis*	313	3.00E-94	38%
SnocOR4	c52458_g1	c52458_g1_i1	2449	384	YES	3.672	4.517	OR78	NP_001177510.1	*Nasonia vitripennis*	91.7	9.00E-16	26%
SnocOR5	c54059_g2	c54059_g2_i1	2189	405	YES	5.838	2.051	OR21	ARO70233.1	*Dendrolimus punctatus*	102	1.00E-19	36%
SnocOR6	c54468_g1	c54468_g1_i1	3602	246	YES	25.894	28.627	OR1	KYM79613.1	*Atta colombica*	392	1.00E-125	57%
SnocOR7	c55521_g1	c55521_g1_i7	2283	381	YES	13.048	9.698	OR294	NP_001177622.1	*Nasonia vitripennis*	123	8.00E-27	26%
SnocOR8	c56607_g1	c56607_g1_i10	1662	406	YES	2.689	1.313	OR18	ARN17891.1	*Cephus cinctus*	342	7.00E-110	48%
SnocOR9	c57498_g11	c57498_g11_i2	3111	432	YES	10.444	3.939	OR13a	XP_012167490.1	*Bombus terrestris*	316	6.00E-95	42%
SnocOR10	c57729_g2	c57729_g2_i2	2020	445	YES	0.861	0.467	OR5	ALD51501.1	*Locusta migratoria*	83.6	2.00E-13	22%
SnocOR11	c57751_g1	c57751_g1_i5	3933	428	YES	4.980	2.473	OR14	AKO89978.1	*Microplitis mediator*	261	2.00E-73	35%
SnocOR12	c58296_g1	c58296_g1_i1	2120	412	YES	40.293	14.332	OR5-T2	RLU18882.1	*Ooceraea biroi*	345	9.00E-109	44%
SnocOR13	c58763_g3	c58763_g3_i4	5705	406	YES	14.461	13.106	OR74	AQN78476.1	*Meteorus pulchricornis*	283	2.00E-81	40%
SnocOR14	c59123_g1	c59123_g1_i3	1732	362	YES	1.093	1.361	OR56	RLZ02203.1	*Cephus cinctus*	228	3.00E-65	37%
SnocOR15	c59331_g1	c59331_g1_i3	4510	392	YES	1.659	1.741	OR34	ARN17905.1	*Cephus cinctus*	234	1.00E-66	45%
SnocOR16	c59347_g1	c59347_g1_i3	4260	381	YES	4.176	1.672	OR23	RLZ02273.1	*Cephus cinctus*	272	1.00E-77	36%
SnocOR17	c59788_g6	c59788_g6_i1	1108	355	NO	6.254	3.281	OR115	NP_001229918.1	*Apis mellifera*	229	3.00E-68	37%
SnocOR18	c59843_g4	c59843_g4_i2	1725	408	YES	1.361	50.789	OR58	AQN78460.1	*Meteorus pulchricornis*	273	2.00E-82	36%
SnocOR19	c60227_g1	c60227_g1_i1	8310	400	YES	18.451	16.682	OR4	AQN78406.1	*Meteorus pulchricornis*	304	8.00E-89	41%
SnocOR20	c60286_g1	c60286_g1_i2	2849	408	YES	14.550	5.341	OR5-T2	RLU18882.1	*Ooceraea biroi*	358	3.00E-111	45%
SnocOR21	c60299_g1	c60299_g1_i1	2237	396	YES	52.889	24.237	OR23	ARN17895.1	*Cephus cinctus*	358	1.00E-113	43%
SnocOR22a	c60686_g2	c60686_g2_i3	1861	323	NO	14.631	3.057	OR5-T2	RLU18882.1	*Ooceraea biroi*	268	5.00E-80	41%
SnocOR22b	c60686_g2	c60686_g2_i7	1039	288	NO	14.631	3.057	OR5-T2	RLU18882.1	*Ooceraea biroi*	266	1.00E-82	44%
SnocOR23	c60686_g3	c60686_g3_i2	1577	409	YES	5.530	1.029	OR5-T2	RLU18882.1	*Ooceraea biroi*	289	3.00E-89	39%
SnocOR24	c61095_g12	c61095_g12_i1	1200	359	NO	6.449	2.431	OR24	ARN17896.1	*Cephus cinctus*	299	3.00E-95	43%
SnocOR25	c61095_g3	c61095_g3_i1	2989	396	YES	8.426	4.883	OR24	ARN17896.1	*Cephus cinctus*	308	1.00E-92	39%
SnocOR26	c61095_g4	c61095_g4_i3	1854	337	YES	1.247	0.177	OR24	ARN17896.1	*Cephus cinctus*	345	1.00E-110	44%
SnocOR27	c61323_g1	c61323_g1_i3	5427	384	YES	6.075	1.907	OR24	ARN17896.1	*Cephus cinctus*	352	1.00E-105	46%
SnocOR28a	c61323_g2	c61323_g2_i1	2630	391	YES	8.815	5.029	OR24	ARN17896.1	*Cephus cinctus*	376	2.00E-119	49%
SnocOR28b	c61323_g3	c61323_g3_i1	1674	391	YES	42.908	17.674	OR24	ARN17896.1	*Cephus cinctus*	393	6.00E-130	51%
SnocOR29	c61095_g7	c61095_g7_i2	1479	147	YES	0.958	0.326	OR24	ARN17896.1	*Cephus cinctus*	124	3.00E-77	45%
SnocOR30	c7309_g1	c7309_g1_i1	1451	412	YES	4.780	123.038	OR1	KYN43233.1	*Trachymyrmex septentrionalis*	220	2.00E-63	30%
SnocOR31	c58763_g3	c58763_g3_i4	5705	129	YES	14.461	13.106	OR1d	AGS43049.1	*Cephus cinctus*	73.6	5.00E-11	37%
SnocOR32	c61095_g7	c61095_g7_i2	1479	84	YES	0.958	0.326	OR15	ARN17889.1	*Cephus cinctus*	69.3	4.00E-11	43%
SnocOR33	c61323_g4	c61323_g4_i1	4503	160	YES	0.665	0.240	OR24	ARN17896.1	*Cephus cinctus*	237	1.00E-65	45%
SnocOR34	c61323_g1	c61323_g1_i3	5427	190	YES	6.075	1.907	OR23	ARN17895.1	*Cephus cinctus*	283	4.00E-83	35%
SnocOR35	c55360_g1	c55360_g1_i6	1688	349	YES	12.319	10.268	OR41	ARN17912.1	*Cephus cinctus*	168	2.00E-43	34%
SnocOR36	c60725_g2	c60725_g2_i2	15905	399	YES	28.301	35.399	OR9	AQN78411.1	*Meteorus pulchricornis*	94	7.00E-17	24%
SnocOR37	c60852_g2	c60852_g2_i4	11915	413	YES	5.853	4.986	OR35	NP_001229900.1	*Apis mellifera*	361	8.00E-109	47%
SnocOR38	c60918_g2	c60918_g2_i9	20641	373	YES	23.466	21.094	OR2	XP_026827586.1	*Ooceraea biroi*	152	4.00E-36	31%
SnitORco	c55324_g1	c55324_g1_i1	4046	481	YES	104.764	87.378	Orco	XP_012273699.1	*Orussus abietinus*	854	0	88%
SnitOR1a	c60056_g1	c60056_g1_i1	2209	164	YES	23.943	15.542	OR13a	XP_012167490.1	*Bombus terrestris*	127	6.00E-55	44%
SnitOR1b	c60056_g1	c60056_g1_i2	2224	294	YES	23.943	15.542	OR5-T2	RLU18882.1	*Ooceraea biroi*	244	6.00E-70	42%

Number	Unigene ID	Transcript ID	Transcript Length（bp）	ORF Length（aa）	Complete ORF	FPKM		Blastx match					
						F	M	Name	Acc.number	Species	Score	E-value	Identity(%)
SnitOR2	c37442_g1	c37442_g1_i1	1677	388	YES	9.169	8.931	OR82a	XP_003393433.	*Bombus terrestris*	327	2.00E-103	42%
SnitOR3	c53338_g1	c53338_g1_i1	1706	407	YES	38.135	10.689	OR77	NP_001164671.1	*Nasonia vitripennis*	310	1.00E-96	38%
SnitOR4	c57594_g2	c57594_g2_i2	2744	384	YES	3.204	5.112	OR78	NP_001177510.1	*Nasonia vitripennis*	96.3	2.00E-17	28%
SnitOR5	c54469_g1	c54469_g1_i3	2179	399	YES	19.505	6.926	OR21	ARO70233.1	*Dendrolimus punctatus*	103	9.00E-20	37%
SnitOR6	c52595_g1	c52595_g1_i1	3650	414	YES	32.996	61.869	OR1	XP_011171479.2	*Solenopsis invicta*	464	6.00E-154	55%
SnitOR7	c52233_g1	c52233_g1_i1	2124	369	YES	7.425	5.217	OR294	NP_001177622.1	*Nasonia vitripennis*	127	3.00E-28	27%
SnitOR8	c60118_g1	c60118_g1_i2	1872	406	YES	2.760	1.420	OR18	ARN17891.1	*Cephus cinctus*	338	2.00E-107	48%
SnitOR9	c59855_g2	c59855_g2_i2	5287	438	YES	3.284	1.211	OR13a	XP_012167490.1	*Bombus terrestris*	317	1.00E-92	42%
SnitOR10	c57074_g1	c57074_g1_i1	2735	445	YES	4.476	1.903	OR	AXM05126.1	*Campoletis chlorideae*	286	8.00E-84	38%
SnitOR11	c55631_g1	c55631_g1_i1	3089	428	YES	8.848	2.976	OR14	AKO89978.1	*Microplitis mediator*	261	3.00E-73	35%
SnitOR12	c59218_g1	c59218_g1_i1	1191	377	NO	10.472	5.262	OR5-T2	RLU18882.1	*Ooceraea biroi*	315	5.00E-101	43%
SnitOR13	c54981_g1	c54981_g1_i1	5681	406	YES	4.295	7.605	OR74	AQN78476.1	*Meteorus pulchricornis*	285	9.00E-82	40%
SnitOR14	c52297_g1	c52297_g1_i2	1929	424	YES	9.866	1.976	OR56	RLZ02203.1	*Cephus cinctus*	263	7.00E-78	36%
SnitOR15	c59143_g1	c59143_g1_i3	7769	392	YES	1.721	2.618	OR34	ARN17905.1	*Cephus cinctus*	328	2.00E-103	52%
SnitOR16	c59920_g4	c59920_g4_i1	7483	381	YES	2.959	1.303	OR23	RLZ02273.1	*Cephus cinctus*	271	2.00E-77	36%
SnitOR17	c59218_g3	c59218_g3_i1	1591	403	YES	45.848	23.789	OR115	NP_001229918.1	*Apis mellifera*	262	5.00E-79	39%
SnitOR18	c59643_g2	c59643_g2_i1	1519	408	YES	1.921	115.206	OR58	AQN78460.1	*Meteorus pulchricornis*	276	1.00E-84	37%
SnitOR19	c58567_g1	c58567_g1_i3	5301	400	YES	12.524	9.263	OR4	AQN78406.1	*Meteorus pulchricornis*	281	6.00E-80	42%
SnitOR20a	c59424_g2	c59424_g2_i2	3072	429	YES	4.819	1.409	OR5-T2	RLU18882.1	*Ooceraea biroi*	357	5.00E-110	45%
SnitOR20b	c59424_g3	c59424_g3_i2	1610	347	YES	3.262	1.927	OR5-T2	RLU18882.1	*Ooceraea biroi*	326	2.00E-103	43%
SnitOR21a	c55031_g1	c55031_g1_i1	3648	396	YES	36.837	17.179	OR23	RLZ02273.1	*Cephus cinctus*	333	3.00E-100	44%
SnitOR21b	c55128_g1	c55128_g1_i1	1512	366	YES	45.542	13.641	OR24	ARN17896.1	*Cephus cinctus*	263	1.00E-79	40%
SnitOR22a	c59218_g2	c59218_g2_i1	1086	351	NO	20.046	14.339	OR5-T2	RLU18882.1	*Ooceraea biroi*	302	1.00E-96	43%
SnitOR22b	c60056_g3	c60056_g3_i1	1533	137	YES	11.882	2.070	OR14	NP_001177470.1	*Nasonia vitripennis*	152	1.00E-37	53%
SnitOR23	c57887_g2	c57887_g2_i4	831	273	NO	3.405	1.493	OR5-T2	RLU18882.1	*Ooceraea biroi*	164	3.00E-44	37%
SnitOR24	c59923_g2	c59923_g2_i2	6092	392	YES	21.673	7.837	OR24	ARN17896.1	*Cephus cinctus*	329	3.00E-107	42%
SnitOR25	c59923_g2	c59923_g2_i2	6092	396	YES	21.673	7.837	OR24	ARN17896.1	*Cephus cinctus*	309	3.00E-99	39%
SnitOR27	c56730_g1	c56730_g1_i1	2018	384	YES	3.807	1.558	OR24	ARN17896.1	*Cephus cinctus*	348	9.00E-111	45%
SnitOR28a	c59052_g1	c59052_g1_i1	1426	391	YES	72.618	35.562	OR24	ARN17896.1	*Cephus cinctus*	383	4.00E-127	49%
SnitOR28b	c59052_g1	c59052_g1_i2	1426	391	YES	72.618	35.562	OR24	ARN17896.1	*Cephus cinctus*	393	3.00E-126	51%
SnitOR29	c36487_g1	c36487_g1_i2	598	138	YES	0.277	0.216	OR24	ARN17896.1	*Cephus cinctus*	125	4.00E-31	45%
SnitOR30	c46348_g1	c46348_g1_i1	1590	412	YES	9.699	201.211	OR1	KYN43233.1	*rachymyrmex septentrionali*	222	1.00E-63	31%
SnitOR31	c51655_g1	c51655_g1_i4	410	82	NO	0.773	1.008	OR1d	AGS43049.1	*Cephus cinctus*	104	1.00E-24	56%
SnitOR32	c53192_g1	c53192_g1_i1	909	93	YES	0.562	0.097	OR15	ARN17889.1	*Cephus cinctus*	68.6	3.00E-09	48%
SnitOR33	c59253_g1	c59253_g1_i4	3343	160	YES	1.166	0.225	OR24	ARN17896.1	*Cephus cinctus*	150	5.00E-78	45%
SnitOR34	c55031_g1	c55031_g1_i1	3648	141	YES	36.837	17.179	OR24	ARN17896.1	*Cephus cinctus*	219	3.00E-62	30%
SnitOR35	c59334_g1	c59334_g1_i6	5043	349	YES	7.386	5.729	OR13a	XP_012167490.1	*Bombus terrestris*	180	5.00E-46	32%
SnitOR36	c57967_g1	c57967_g1_i3	3667	398	YES	2.545	1.828	OR9	AQN78411.1	*Meteorus pulchricornis*	97.4	3.00E-18	24%
SnitOR37	c58487_g2	c58487_g2_i14	7888	413	YES	23.421	23.000	OR35	NP_001229900.1	*Apis mellifera*	362	6.00E-108	47%
SnitOR38	c59363_g1	c59363_g1_i3	19836	373	YES	32.325	34.126	OR82a	XP_019696528.1	*Harpegnathos saltator*	147	4.00E-36	31%

Table S4. Sequence information and best blasts match information of sensory neuron membrane proteins (SNMPs).

Number	Unigene ID	Transcript ID	Transcript Length（bp）	ORF Length（aa）	Complete ORF	FPKM		Blastx match					
						F	M	Name	Acc.number	Species	Score	E-value	Identity(%)
SnocSNMP1	c60539_g2	c60539_g2_i2	3404	526	YES	330.887	543.047	SNMP1	XP_015587038.1	*Cephus cinctus*	457	3.00E-144	69%
SnitSNMP1	c56105_g1	c56105_g1_i2	5629	526	YES	366.54	879.837	SNMP1	XP_012278791.2	*Orussus abietinus*	788	0	67%

Table S5. Sequence information and best blasts match information of gustatory receptors (GRs).

Number	Unigene ID	Transcript ID	Transcript Length （bp）	ORF Length （aa）	Complete ORF	FPKM		Blastx match					
						F	M	Name	Acc.number	Species	Score	E-value	Identity(%)
SnocGR1	c101886_g1	c101886_g1_i1	207	67	NO	0.000	0.000	GR22	XP_012265848.2	*Athalia rosae*	122	2.00E-31	78%
SnocGR2	c45400_g1	c45400_g1_i1	1900	179	YES	21.104	26.386	GR24	XP_012260950.1	*Athalia rosae*	297	1.00E-90	82%
SnocGR3	c50214_g1	c50214_g1_i1	1136	275	YES	0.297	0.209	GR22	XP_012265848.2	*Athalia rosae*	441	4.00E-150	87%
SnocGR4	c58124_g1	c58124_g1_i1	5307	431	YES	10.204	10.342	GR2	ALG36126.1	*Sclerodermus sp. MQW-2015*	99.8	2.00E-19	35%
SnocGR5	c59973_g1	c59973_g1_i6	2810	432	YES	1.062	1.478	GR5a for trehalose	XP_023290777.1	*Orussus abietinus*	611	0.00E+00	66%
SnocGR6	c59973_g2	c59973_g2_i1	573	163	NO	0.210	0.292	GR5a for trehalose	XP_012278986.1	*Orussus abietinus*	93.2	1.00E-18	40%
SnocGR7	c66796_g1	c66796_g1_i1	438	132	NO	0.000	0.322	GR24	XP_012260950.1	*Athalia rosae*	207	1.00E-62	62%
SnocGR8	c69089_g1	c69089_g1_i1	232	75	NO	0.000	1.235	GR3	ALG36127.1	*Sclerodermus sp. MQW-2015*	95.9	7.00E-23	54%
SnitGR3	c59968_g2	c59968_g2_i5	1483	453	YES	1.487	0.443	GR22	XP_012265848.2	*Athalia rosae*	696	0	80%
SnitGR4	c57452_g3	c57452_g3_i1	5344	431	YES	15.900	25.558	GR2	ALG36126.1	*Sclerodermus sp. MQW-2015*	96.3	3.00E-18	36%
SnitGR5a	c56737_g1	c56737_g1_i2	2592	170	YES	0.465	1.017	GR5a for trehalose	XP_023290777.1	*Orussus abietinus*	271	3.00E-86	80%
SnitGR5b	c33899_g1	c33899_g1_i1	383	126	NO	0.116	0.759	GR5a for trehalose	XP_023290777.1	*Orussus abietinus*	189	5.00E-55	71%
SnitGR6	c50769_g1	c50769_g1_i2	577	163	NO	0.190	0.615	GR5a for trehalose	XP_012278986.1	*Orussus abietinus*	96.3	1.00E-19	41%
SnitGR7	c51382_g1	c51382_g1_i1	669	130	YES	0.271	0.191	GR24	XP_012260950.1	*Athalia rosae*	238	2.00E-73	58%
SnitGR8	c21652_g1	c21652_g1_i1	609	140	NO	0.835	1.172	GR43a for sugar taste	XP_012270798.1	*Orussus abietinus*	228	3.00E-69	54%
SnitGR9	c44554_g1	c44554_g1_i3	1061	161	YES	0.412	0.147	GR43a for sugar taste	XP_012270798.1	*Orussus abietinus*	230	1.00E-67	60%
SnitGR10	c56368_g1	c56368_g1_i1	2815	139	YES	4.356	3.326	GR43a for sugar taste	XP_012270798.1	*Orussus abietinus*	92	2.00E-15	41%
SnitGR11	c56737_g1	c56737_g1_i2	2592	270	YES	0.465	1.017	GR5a for trehalose	XP_012278986.1	*Orussus abietinus*	335	5.00E-110	65%

Table S6. Sequence information and best blasts match information of ionotropic receptors (IRs).

**Number**	**Unigene ID**	**Transcript ID**	**Transcript Length （bp）**	**ORF Length （aa）**	**Complete ORF**	**FPKM**		**Blastx match**					
						**F**	**M**	**Name**	**Acc.number**	**Species**	**Score**	**E-value**	**Identity(%)**
SnocIR1	c48866_g1	c48866_g1_i2	1390	81	YES	0.690	0.655	IR	EZA49990.1	*Ooceraea biroi*	58.2	4.00E-06	35%
SnocIR2	c55219_g2	c55219_g2_i1	3207	719	YES	1.344	1.840	IR2	XP_012273738.1	*Orussus abietinus*	979	0	69%
SnocIR3	c56494_g7	c56494_g7_i1	1552	373	NO	5.851	8.756	IR6	ARN17852.1	*Cephus cinctus*	499	5.00E-168	61%
SnocIR4	c58718_g2	c58718_g2_i2	4805	613	YES	12.889	9.015	IR1	ARN17847.1	*Cephus cinctus*	978	0	78%
SnocIR5	c59413_g1	c59413_g1_i4	4190	917	YES	0.832	0.832	IR2	XP_012273732.1	*Orussus abietinus*	1206	0	66%
SnocIR6	c59444_g2	c59444_g2_i2	4518	705	YES	16.629	16.088	IR2	ARN17848.1	*Cephus cinctus*	1057	0	77%
SnocIR7	c60562_g3	c60562_g3_i2	820	152	NO	0.861	1.075	IR2	XP_012273728.1	*Orussus abietinus*	294	6.00E-90	86%
SnocIR8	c60562_g4	c60562_g4_i3	3771	642	YES	1.074	0.797	IR2	XP_012263948.1	*Athalia rosae*	1351	0	95%
SnocIR9	c61059_g3	c61059_g3_i1	4632	914	YES	0.943	0.874	IR2	KZC14133.1	*Dufourea novaeangliae*	1718	0	91%
SnocIR10	c61207_g1	c61207_g1_i3	5402	1134	YES	0.667	0.944	IR NMDA 2B	XP_020280170.1	*Pseudomyrmex gracilis*	1152	0	87%
SnocIR11	c56517_g1	c56517_g1_i1	3541	874	YES	2.149	2.864	IR3	ARN17849.1	*Cephus cinctus*	1089	0	61%
SnocIR12	c58489_g4	c58489_g4_i2	7649	1019	NO	4.343	3.626	IR NMDA 3A	EZA62306.1	*Ooceraea biroi*	1519	0	77%
SnocIR13	c56460_g2	c56460_g2_i3	1138	333	YES	0.244	0.286	IR1	ARN17847.1	*Cephus cinctus*	648	0	93%
SnitIR1	c61375_g1	c61375_g1_i1	247	73	NO	0.000	1.781	IR	EZA49990.1	*Ooceraea biroi*	85.5	5.00E-19	46%
SnitIR2	c58928_g5	c58928_g5_i5	4546	905	YES	1.780	1.146	IR2	XP_012273738.1	*Orussus abietinus*	1211	0	66%
SnitIR3	c54076_g4	c54076_g4_i1	1891	382	NO	8.352	13.977	IR6	ARN17852.1	*Cephus cinctus*	513	1.00E-171	62%
SnitIR4	c56458_g1	c56458_g1_i4	5392	613	YES	9.697	9.475	IR1	ARN17847.1	*Cephus cinctus*	978	0	78%
SnitIR5	c53729_g1	c53729_g1_i4	2959	917	YES	0.840	0.399	IR2	OAD59458.1	*Eufriesea mexicana*	740	0	73%
SnitIR6	c56562_g2	c56562_g2_i1	4589	829	YES	18.802	24.364	IR2	ARN17848.1	*Cephus cinctus*	1279	0	80%
SnitIR8	c58928_g3	c58928_g3_i7	6131	860	YES	4.406	5.109	IR2	XP_012263948.1	*Athalia rosae*	1602	0	93%
SnitIR9	c59833_g2	c59833_g2_i2	4777	921	YES	5.279	3.599	IR2	KZC14133.1	*Dufourea novaeangliae*	1718	0	92%
SnitIR10	c60102_g2	c60102_g2_i1	7186	1134	YES	1.582	2.930	IR NMDA 2B	XP_020719424.1	*Bombus terrestris*	1796	0	83%
SnitIR11	c54545_g1	c54545_g1_i1	6307	802	YES	5.475	15.750	IR3	ARN17849.1	*Cephus cinctus*	1087	0	61%
SnitIR12	c56707_g1	c56707_g1_i3	13901	1350	YES	3.607	5.375	IR NMDA	PBC30154.1	*Apis cerana cerana*	1980	0	74%
SnitIR13	c57346_g2	c57346_g2_i2	4250	972	YES	2.802	4.447	IR1	XP_012166058.1	*Bombus terrestris*	1754	0	89%
SnitIR14	c88251_g1	c88251_g1_i1	238	71	NO	0.000	2.017	IR2	XP_023288447.1	*Orussus abietinus*	167	2.00E-46	99%
SnitIR15	c102040_g1	c102040_g1_i1	252	70	NO	1.987	0.000	IR	PBC33125.1	*Apis cerana cerana*	174	8.00E-52	98%
SnitIR16	c36298_g1	c36298_g1_i1	256	82	NO	0.000	0.000	IR2	PNF32816.1	*Cryptotermes secundus*	173	2.00E-52	95%
SnitIR17	c56458_g1	c56458_g1_i4	5392	270	YES	9.697	9.475	IR1	ARN17847.1	*Trachymyrmex septentrionalis*	356	2E-113	65%
