# Supplementary material for: Antennal transcriptome analysis of olfactory genes and characterizations of odorant binding proteins in two woodwasps, Sirex noctilio and Sirex nitobei (Hymenoptera: Siricidae)
Source: BMC Genomics. 2021 Mar 10;22:172. doi: 10.1186/s12864-021-07452-1 (PMC7945326; doi:10.1186/s12864-021-07452-1)
Supplement: Supplementary file 3 — Additional file 3. The sequence of OBPs with alignment were shown six conserved cysteine residues. [file 12864_2021_7452_MOESM3_ESM.pdf]

```
                  10        20        30        40        50        60        70        80        90       100
         ....|....|....|....|....|....|....|....|....|....|....|....|....|....|....|....|....|....|....|....|
SnocOBP1  DEATSSSDAESLITSTTLSPASNESDAARSAIKEQLAKLTESCKTSSQANSDDAKIIGTESVPKTEG-----EKCFLQCVYTGLGIVKND-QFSVEGARL
SnocOBP2  --------------------------------MKGDLSILRLCNASSPVSLEAVNSVLIYRHMQHRETKTKSFKCFLLCLYVEYDWMDREGS----FKLN
SnocOBP3  ----------------------VPVDEVEPSEETKNADIRKFCRESTGITLEEIEQLRKDPKKNVLE----SGKCYVKCMAERSGVEKDGEV------QM
SnocOBP4  -------------------------AMTKEQLDSMVKALRKTCQSKTEVDTAILDGMRRGEFPEDPK-----FQCYLKCLLKTSRAIRDDKLD---LNMM
SnocOBP5  -------------------------------VKPVNKEHRSTCLKENPISDAVLADLRDAKFADHTD----DVKCYVACLMKISGVLKDGAY------NV
SnocOBP6  -------------------------GAIPDDMKAFAKTLRDICIAESGTMKEYIEKASKGEFTNDE-----KLKCYFKCLFEKLDLITEAGE----LDYE
SnocOBP7  -------------------------------------DIRRDCRKQTNVSWASLKKLRAGDFNQDDQ----KLKCYLKCFMVKNNIIDDNSR----VETD
SnocOBP8  -------------------------------MIKEFKELIDQCAAEMDVTEEQLKMMRDKMVNEEDER---KIACMKACILQKAGIMVSSKLLKEEIDNL
SnocOBP9  ----------------------KLPDWVPPDIIELVAEDKKRCMDEHKVDQATIDKADNGDIPNEQ-----NIKCYMHCMMESFSVIDENGE----IEEE
SnocOBP10 ---------------------GTRPSFVSDEMIMTAASVVNACQTQTGVATADIEAVRNGKWSEER-----SLKCYMYCLWEQFGLVDDKSD----LSLN
SnocOBP11 --------------------------------ARPKAETKEECYKSSGLTADDVKAVKSSSLTPEQQK---VLGAYFACFLKDRGILSDNGHFDIEAS--
SnocOBP12 --------------------------MTRAQIENGMKIMRKVCQPKFGVSNDILDAAMEGHFPADRA-----LQCYQKCVLGLMKVLKNDKIQ---LESL
SnocOBP14 -----------------LTPAN----------RVKVIAANVRCLEESGLDPEILERTKNGEDVDDKN-----LDCFGACLLKDFGILNNDGTFNKDEA--
SnocOBP15 ---------------------------YSEEIEEIMRLLHDTCVSQTGVDENLIAKIRVGDFVDDN-----NLKCYLRCLMVEVSSMDENGI----VDEE
SnocOBP16 ---------------------IDRPIFITERMLQAARPMKEICYELANVSEEEVNEASNGNFIAGN-----NIGCYTHCIWERMGLLDELDN----VKTN
SnitOBP1  DEATSSSDAESLITSTTLSPASNETDAARSAIKEQLAKLTESCKTSSQANSDDAKIIGTESVPKTEG-----EKCFLQCVYTGLGIVKND-QFSVEGARL
SnitOBP2  ----------------------------FTSSMKGDLSILRLCNASSPVSLEAVNSVLIYRHMQHRETKTKSFKCFLLCLYVEYDWMDREGS----FKLN
SnitOBP3  ----------------------VPVDEVEPSEETKNADIRKFCRESTGITLEEIEQLRKDPKNNVLE----SGKCYVKCMAERSGVEKDGEV------QM
SnitOBP4  -------------------------AMTKEQLDSMIKALRKTCQSKTEVDTAILDGMRRGEFPEDPK-----FQCYLKCLLKTSRAIRDDKLD---LNMM
SnitOBP5  -------------------------------VKPVNKEHRSTCLKENPISDAVLADLRDAKFADHTD----DVKCYVACLMKISGVLKDGAY------NV
SnitOBP6  -------------------------GAIPDDMKAFAKALRDICIAESGTMKEYIEKASKGEFTNDE-----KLKCYFKCLFEKLDLITEAGE----LDYE
SnitOBP7  -------------------------------------DIRRDCRKQTNVSWASLKKLRAGDFNQDDQ----KLKCYLKCFMVKNNIIDDNSR----VETD
SnitOBP8  --------------------------EKNHEMMKEFKELIDQCAAEMDVTEEQLKMMRDKMVNEEDER---KIACMKACILQKAGIMVSSKLSKEEIDNL
SnitOBP9  ----------------------KLPDWAPPDIIELVAEDKKRCMDEHKVDQATIDKADNGDIPNEQ-----NIKCYMHCMMESFSVVDEDGE----IEEE
SnitOBP10 ---------------------GTRPSFVSDEMIMTAASVVNACQTQTGVATADIEAVRNGKWSEER-----SLKCYMYCLWEQFGLVDDKSD----LSLN
SnitOBP11 --------------------------------ARPKAETKEECYKSSGLTADDVKAVKSSSLTPEQQK---VLGAYFVCFLKDRGILSDNGHFDIEAS--
SnitOBP12 --------------------------MTRAQIENGMKIMRKVCQPKFGVSNDILDAAMEGHFPADRA-----LQCYQKCVLGLMKVLKNDKVQ---LESL
SnitOBP14 -----------------LTPAN----------RVKVIAANVRCLEESGLDPEILERTKNGEDVDDKN-----LDCFGACLLKDFGILNDDGTFNKDEA--
SnitOBP15 ---------------------------YSEEIEEIMRLLHDTCVSQTGVDENLIAKIRVGDFVEDN-----NLKCYLRCLMVEVSSMDENGI----VDEE
SnitOBP16 ---------------------IDRPIFITERMLQAARPMKEICYELANVSEEEVNEASNGNFIAGN-----NIGCYTHCIWERMGLLDEFDN----VKTN
```

```
                    110       120       130       140       150       160       170
           ....|....|....|....|....|....|....|....|....|....|....|....|....|....|....|
SnocOBP1   LAQKRFGAFPEELEKANQLIETCSKEAVKKDSKDKCPMGFLIRQCFVKNGQKINFFPKA----------------
SnocOBP2   NIKSSLQSTIVEDHHVKVLIYKCT----AIELIDPCDRAFHFTECFWSQDDEEKDSKANITEKKTKDELSGFYHT
SnocOBP3   DVAIVKRPEGVSQEDVEKLISECSGETKS---DDLCERSFQRYICFFEKSEKKITIT------------------
SnocOBP4   LKQADIMIADEFQERTKSAMRKCTAETSS---SDMCVAAYQFFKCFWETDSEMFIFM------------------
SnocOBP5   NAALEFFPDDV-QSVFKSVFDKCVGQVNDKGATEDCEIAKIMFQCFYDVEPSLLEAGDFF---------------
SnocOBP6   KMMDFAP--KFLKQSAMKMIENCR----STTGTDLCDTAFKVNKCFQKTDPTTYFVI------------------
SnocOBP7   KALRHLP--PKFQESSRRILDRCK----NSPGNDSCDTAFQVAKCYFRSQPEILKQIAFV---------------
SnocOBP8   VQKLHFDDDEN-REKMQKTVSDCYDEVQN--LEDECEIAKSFTKCFEHGKSD-----------------------
SnocOBP9   TFVGFLP--EQFQTKARQSLSACA----NKGGADPCDKLYNTMMCFIPLAPELWYVL------------------
SnocOBP10  GMLTFFQRIPAYRIEVQTAVNECKGISKYLVHGDNCEYAYVFNKCYAKRSPKTYYLF------------------
SnocOBP11  --KKDIEP--EHLEIALPVLEVCG----KDEGSNALETTMKLLMCTKEKSG-NVLFETLDLF-------------
SnocOBP12  LKEIGKILPSDMIERSKEVSIECVPKATS---EDACEAAWQFVKCYYETDKTMYIFA------------------
SnocOBP14  --VKNLPDS-VRNDDVINMINACS----NKKGETDCDTAHLIFSCIREKAGFKYIQEEFRDI-------------
SnocOBP15  MVVNAMP--EELIEFAEPVIRSCG----TVKGVDDCDTAFQTNKCYYFKDPEHYLAV------------------
SnocOBP16  VLENLLP--ENVKDEIISTLIVCQ--RQNPAMSDKCSRTLAILKCYCSLSPKTYYLM------------------
SnitOBP1   LAQKRFGAFPEELEKANQLIETCSKEAVKKDSKDKCPMGFLIRQCFVKNGQKINFFPKA----------------
SnitOBP2   NIKSSLQSTIVEDHHVKVLIYKCT----AIELIDPCDRAFHFTECFWSQDDKEKDSKANITEKKTKDELSGFYHT
SnitOBP3   DVAIVKRPEGVSQEDVEKLISECSGETKS---DDLCERSFQRYICFFEKSEKKITIT------------------
SnitOBP4   LKQADIMIAGEFQERTKSVMRKCTAETSS---SDMCVAAYQFFKCFWETDSEMFIFM------------------
SnitOBP5   NAALEFFPDDV-QSVFKSVFDKCVAQVNDKGATEDCEIAKIMFQCFYDVEPSLLEAGDFF---------------
SnitOBP6   KMMDFAP--KFLKQSAMKMIENCR----STTGTDLCDTAFKVNKCFQKTDPTTYFVI------------------
SnitOBP7   KALRHLP--PKLQESSRRILDRCK----NSPGNDSCDTAFQVAKCYFRSQPEILKQIAFV---------------
SnitOBP8   VQKLHFDDDEN-REKMQKTASDCYDEVQN--LEDECEIAKSFTKCFEHGKSN-----------------------
SnitOBP9   TFVGFLP--EQFQTKARQSLSACA----NKGGADPCDKLYNTMMCFIPLAPELWYVL------------------
SnitOBP10  GMLTFFQRIPAYRIEVQTAVNECKGISKYLVHGDNCEYAYVFNKCYAKRSPKTYYLF------------------
SnitOBP11  --KKDIEP--EHLEIAQPVLEVCG----KDEGSNALETTMKLLMCTKEKSG-NVLFETLDLF-------------
SnitOBP12  LKEIGKILPPDMIERSKEVSIECVPKATS---EDACEAAWQFVKCYYETDKTMYIFA------------------
SnitOBP14  --VKNLPDS-VRNDDVINMINACS----NKKGETDCDTAYLIFSCIREKAGFKYIQEEFRDI-------------
SnitOBP15  MVVNAMP--EELIEFAEPVIRSCG----TVKGVDDCDTAFQTNKCYYFKDPEHYLAV------------------
SnitOBP16  VLENLLP--EDVKNEIISTLIVCQ--RQNPAMSDKCSRTLAILKCYCSLSPKTYYLM------------------
```
