# Supplementary material for: Antennal transcriptome analysis of olfactory genes and characterizations of odorant binding proteins in two woodwasps, Sirex noctilio and Sirex nitobei (Hymenoptera: Siricidae)
Source: BMC Genomics. 2021 Mar 10;22:172. doi: 10.1186/s12864-021-07452-1 (PMC7945326; doi:10.1186/s12864-021-07452-1)
Supplement: Supplementary file 4 — Additional file 4. The sequence of CSPs with alignment were shown four conserved cysteine residues. [file 12864_2021_7452_MOESM4_ESM.pdf]

```
                  10        20        30        40        50        60        70        80        90       100
         ....|....|....|....|....|....|....|....|....|....|....|....|....|....|....|....|....|....|....|....|
SnocCSP1 KPKPAEKKQYTTKYDNIDLDEILNNQRLFDNYYKCLLG---GKCTPDGQELREALPDALATACSKCTEKQRVGTEKVIKYLIEKKPTEYSELEKKYDPQG
SnocCSP2 ------IEKYSSRYDDVDVGRILANNRVLTAYIRCMLDE--GSCTAEGRELKKTLPDALATGCSKCNEKQKVMAQKVIDHIQKKMPTDWNRLIVKYDPQG
SnocCSP3 -YLWPKQDTYTTRWDKVNVDDILDSKRLLQYYFNCLMS--RGPCPPDGQELRRVLPEALNTACAKCTKSQIEGSVKVIRYLREFEPKKFENLADKYDPQG
SnocCSP4 --QQQGHDYYTGRWNDINTKDIIDNARLFKKYKECVISNSAVGCPKEALELKRVLPEALETVCAKCSHVQVTKVQDTLSHICKTRKPDFDEILAKIDPEK
SnocCSP5 --QKTEKQSQTSRVTDDQLDVALSDERYLRRQLKCALG--EAPCDPVGRRLKSLAPLVLRGACPQCSPEEIRQIKKVLSHIQRSFPKEWAKVVQQY--AG
SnitCSP2 ------IEKYSSRYDDVDVGRILANNRVLTAYIRCMLDE--GSCTAEGRELKKTLPDALATGCSKCNEKQKVMAQKVIDHIQKKMPTDWNRLIVKYDPQG
SnitCSP3 -YLWPKQDTYTTRWDKVNVDDILDSKRLLQYYFNCLMS--RGPCPPDGQELRRVLPEALNTACAKCTKSQIEGSVKVIRYLREFEPKKFENLADKYDPQG
SnitCSP4 --QQQGQDYYTGRWNDINTKDIIDNARLFKKYKECVISNSAVGCPKEALELKRVLPEALETVCAKCSPVQVTKVQDTLSHICKTRKPDFDEILAKIDPEK
SnitCSP5 --QKTEKQSQTSRVTDDQLDVALSDERYLRRQLKCALG--EAPCDPVGRRLKSLAPLVLRGACPQCSPEEIRQIKKVLSHIQRSFPKEWAKVVQQY--AG

                 110
         ....|....|....|..
SnocCSP1 NYKRKYQAEAAKRGIKV
SnocCSP2 EYKKRYDTLQAAKKI--
SnocCSP3 IYRRRYLEPPPDNNTA-
SnocCSP4 TFRPRFEEKFGKLNC--
SnocCSP5 V----------------
SnitCSP2 EYKKRYDTLQAAKNI--
SnitCSP3 IYRRRYLEPPPDNNTA-
SnitCSP4 TFRPRFEEKFGKLNC--
SnitCSP5 V----------------
```
