# Supplementary figures and images for: Antennal transcriptome analysis of olfactory genes and characterizations of odorant binding proteins in two woodwasps, Sirex noctilio and Sirex nitobei (Hymenoptera: Siricidae)
Source: BMC Genomics. 2021 Mar 10;22:172. doi: 10.1186/s12864-021-07452-1 (PMC7945326; doi:10.1186/s12864-021-07452-1)

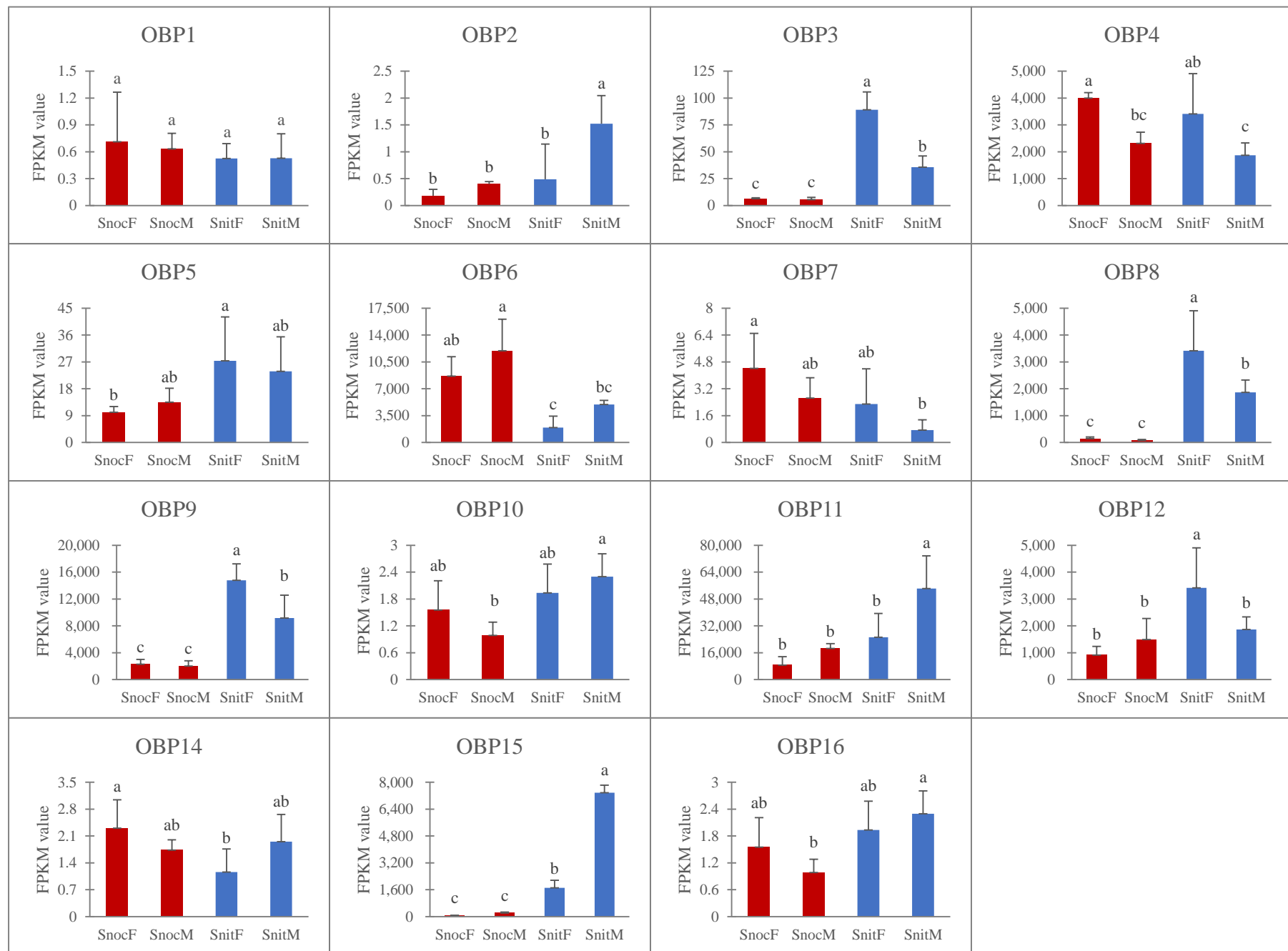

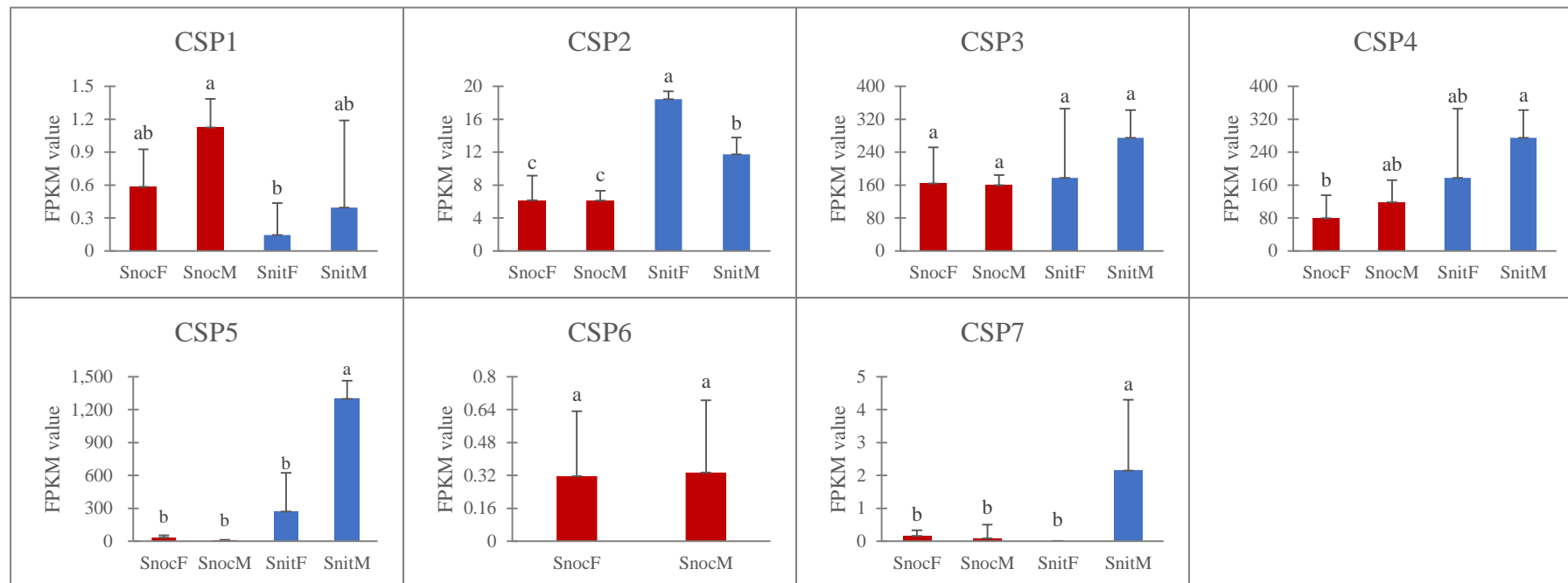

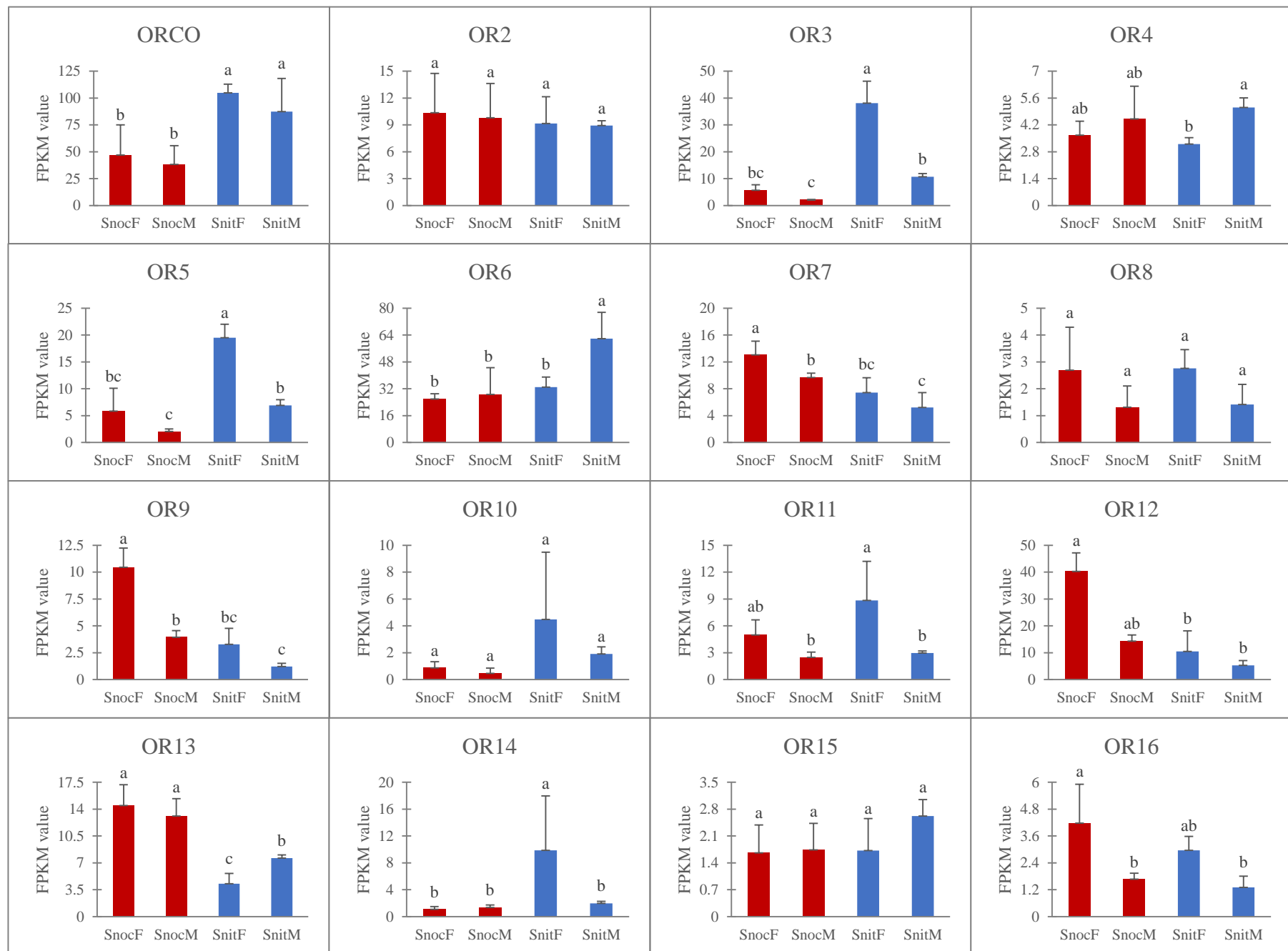

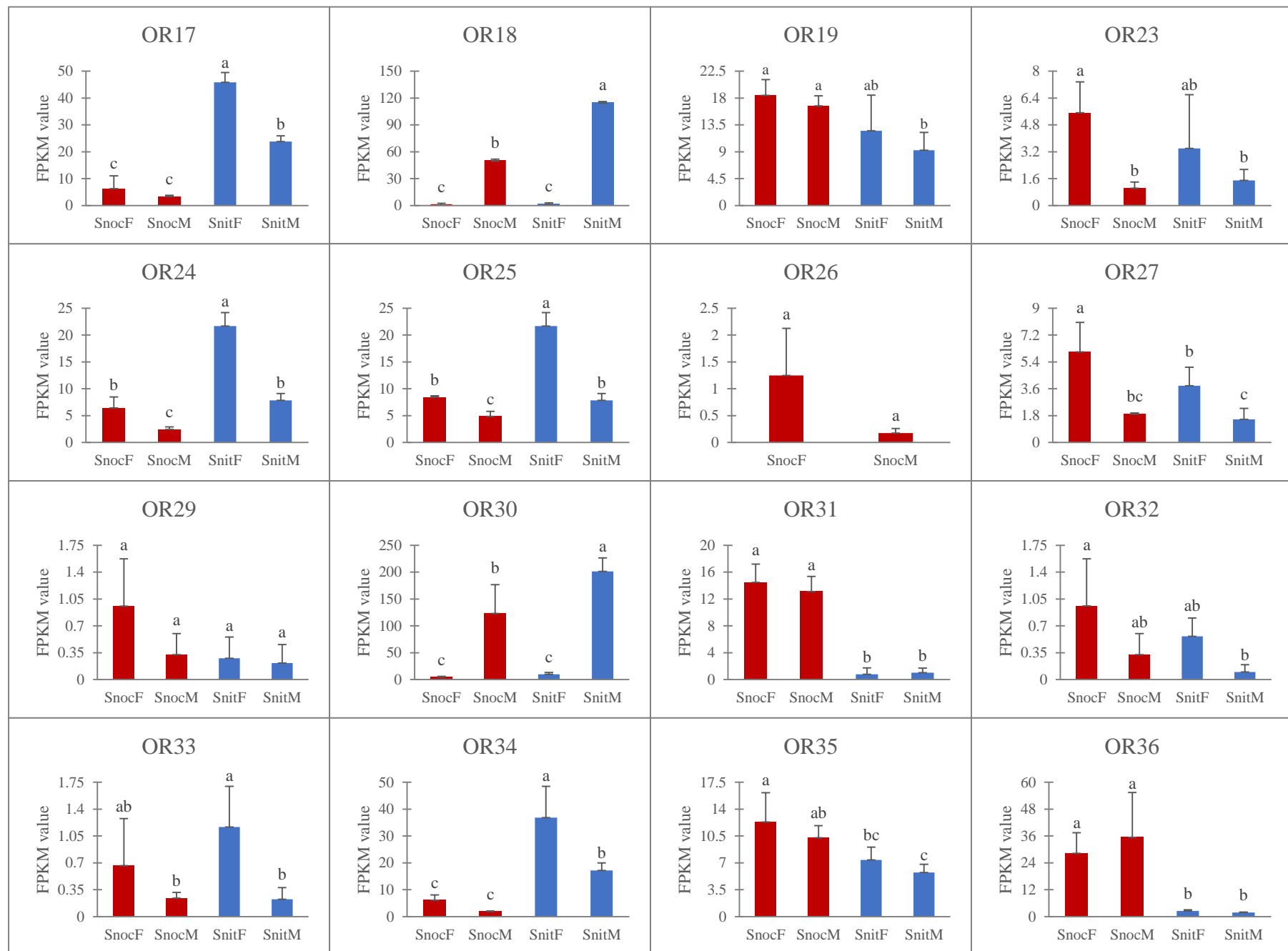

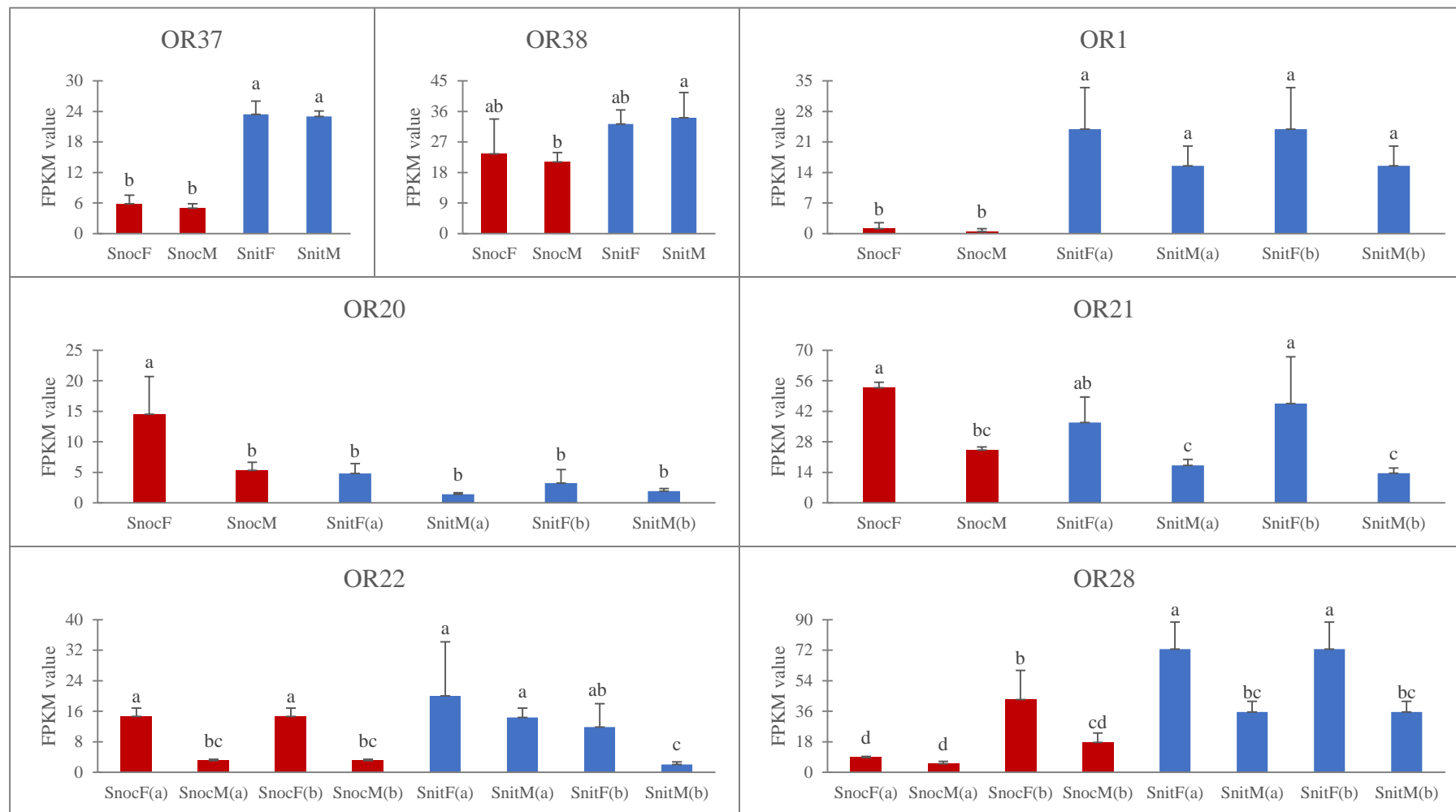

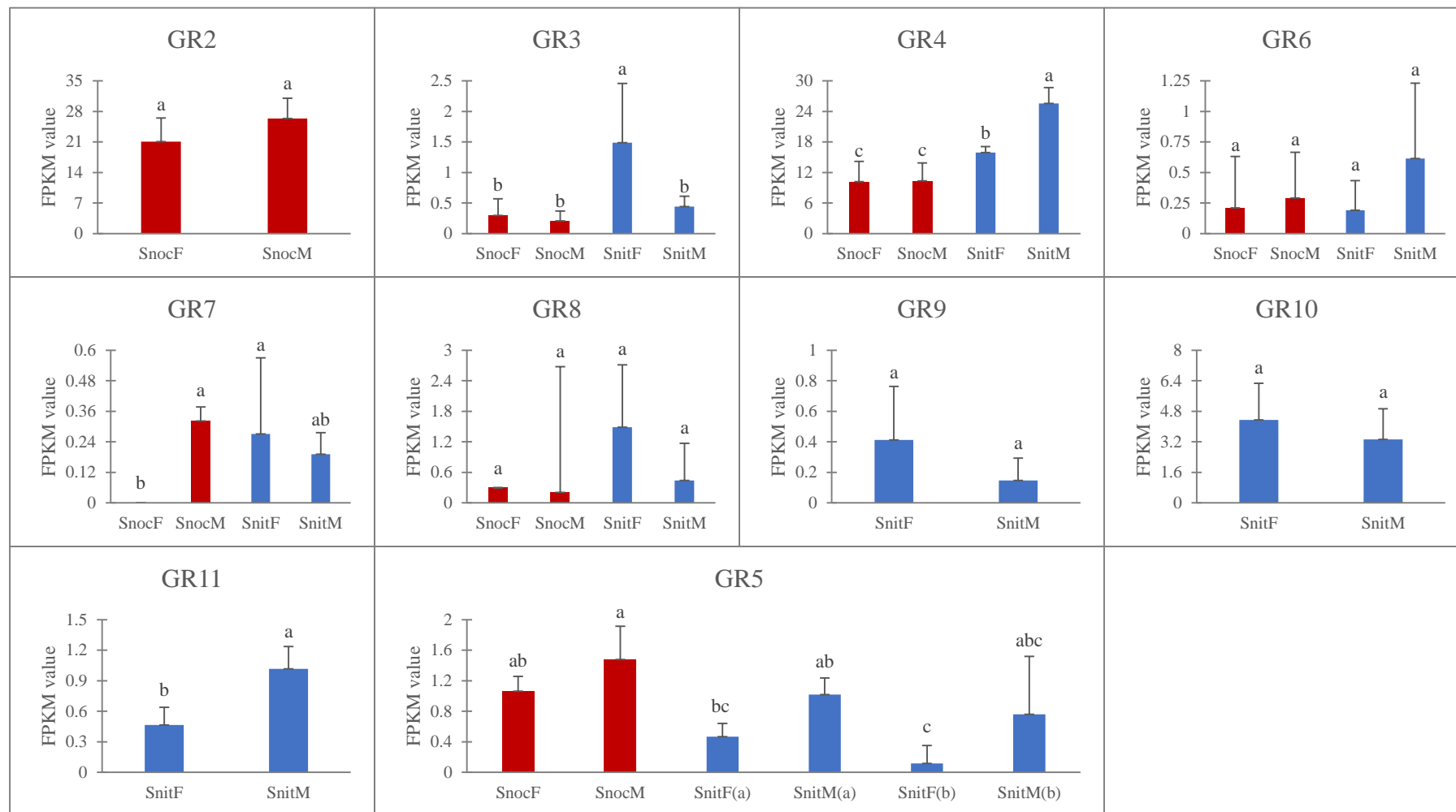

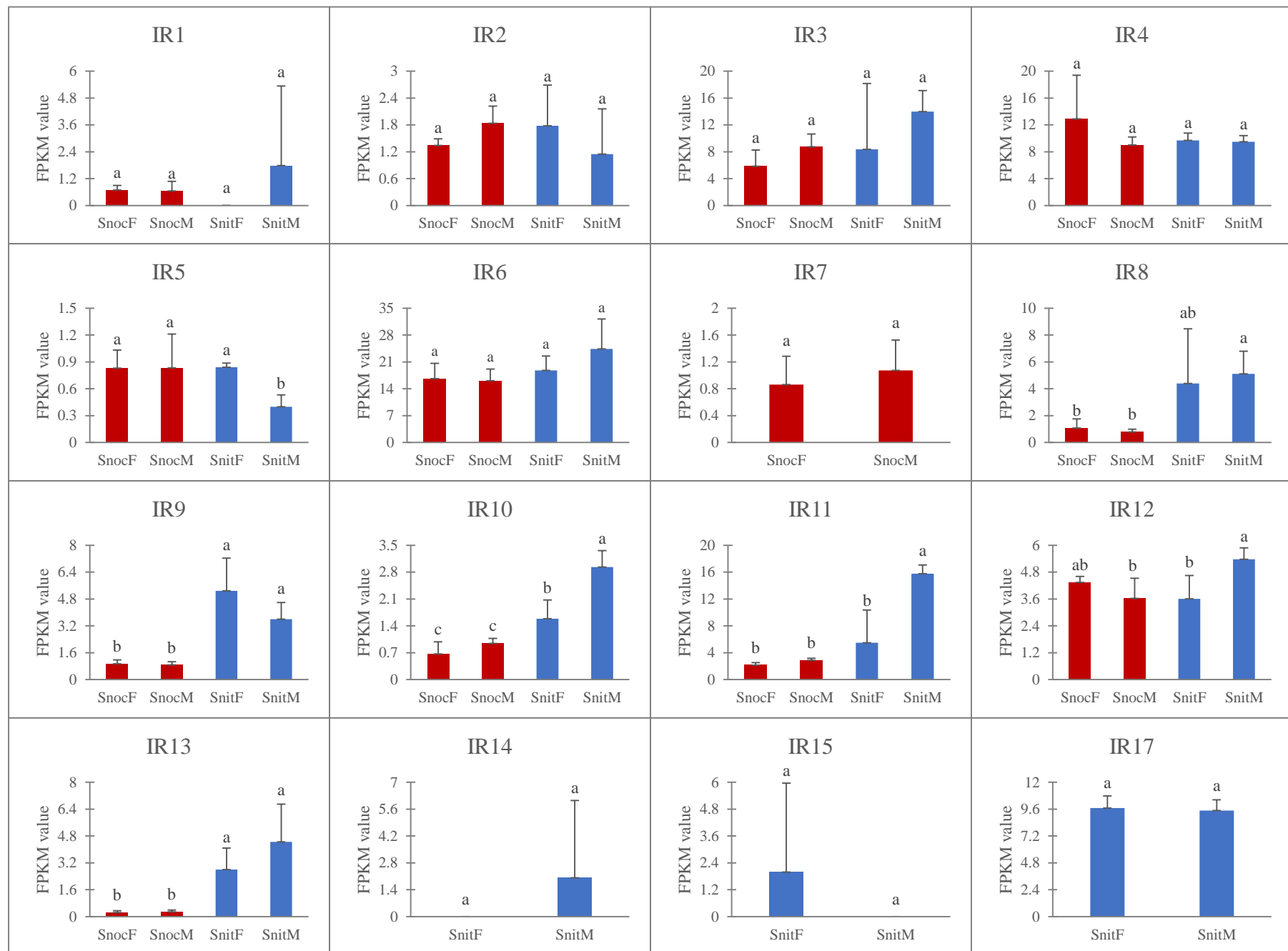

Supplement: Supplementary file 5 — Additional file 5. Significance test of The FPKM value of homologous genes (OBPs, CSPs, ORs, SNMPs, GRs and IRs) between two siricid species and male and female. S. noctilio data is colored red and S. nitobei data is colored blue. [file 12864_2021_7452_MOESM5_ESM.pdf]
