# Supplementary material for: Antennal transcriptome analysis of olfactory genes and characterizations of odorant binding proteins in two woodwasps, Sirex noctilio and Sirex nitobei (Hymenoptera: Siricidae)
Source: BMC Genomics. 2021 Mar 10;22:172. doi: 10.1186/s12864-021-07452-1 (PMC7945326; doi:10.1186/s12864-021-07452-1)
Supplement: Supplementary file 6 — Additional file 6. Primers of odorant binding proteins and reference gene for quantitative real-time PCR. [file 12864_2021_7452_MOESM6_ESM.pdf]

Primer	Sequence(5'to3')
SnocOBP3_f	GGAGGAGACGAAGAATGCAG
SnocOBP3_r	GACTATGGCCACGTCCATTT
SnocOBP4_f	GAGGTCGACACTGCGATTCT
SnocOBP4_r	GATCATTATGTCCGCCTGCT
SnocOBP6_f	TTGCAGAAAGTGGAACGATG
SnocOBP6_r	GCGCAAAGTCCATCATTTTT
SnocOBP7_f	TCGAGTGGAGACCGATAAGG
SnocOBP7_r	TCTCGGGCTGAGACCTAAAG
SnocOBP8_f	GTCAACGAGGAGGACGAGAG
SnocOBP8_r	CTCCAGGTTTTGCACCTCAT
SnocOBP9_f	GCAACGATTGACAAAGCAGA
SnocOBP9_r	TTTGTCGCGCTTTAGTCTGA
SnocOBP10_f	GGGAACAATTTGGTTTGGTG
SnocOBP10_r	ACGCGTACTCGCAGTTATCA
SnocOBP11_f	ACGAATGTTGCAAGCAGCTA
SnocOBP11_r	CAATCCCATTCTTTCCCAAA
SnocOBP12_f	TCGTCATCAGCATCGAAAAC
SnocOBP12_r	ACCTTCATCAGTCCCAGCAC
SnocOBP15_f	ATTTCGGCATTTTGAACGAC
SnocOBP15_r	TGTACTTGAAGCCCGCTTTT

Primer	Sequence(5'to3')
SnitOBP3_f	GGAGGAGACGAAGAATGCAG
SnitOBP3_r	GACTATGGCCACGTCCATTT
SnitOBP4_f	GAGGTCGACACTGCGATTCT
SnitOBP4_r	GATCATTATGTCCGCCTGCT
SnitOBP6_f	ATCGCAGAAAGTGGAACGAT
SnitOBP6_r	GCGCAAAGTCCATCATTTTT
SnitOBP7_f	GGGCATCCTTGAAGAAACTG
SnitOBP7_r	TCCGCGAAGACTCCTGTAAT
SnitOBP8_f	GTCAACGAGGAGGACGAGAG
SnitOBP8_r	CTCCAGGTTTTGCACCTCAT
SnitOBP9_f	GCAACGATTGACAAAGCAGA
SnitOBP9_r	TTTGTCGCGCTTTAGTCTGA
SnitOBP10_f	GGGAACAATTTGGTTTGGTG
SnitOBP10_r	ACGCGTACTCGCAGTTATCA
SnitOBP11_f	TGCAAGCAGCTAGACCAATG
SnitOBP11_r	CCAGGACGTTTGTTTTCACA
SnitOBP12_f	GCCTGAACAGCAGAAGGTCT
SnitOBP12_r	TGTAGTTTCCAGTGCGTTGC
SnitOBP15_f	ATTTCGGCATTTTGAACGAC
SnitOBP15_r	TGTACTTGAAGCCCGCTTTT

Primer	Sequence(5'to3')
β-tubulin_f	TGGAAATTCTACGGCCATTC
β-tubulin_r	CTGCTGATACTCCGACACCA
α-tubulin_f	CAGAGAAGGCCTACCACGAG
α-tubulin_r	GTTTACGTCCTTGGGAACGA
actin1_f	CGAGAAGATGACGCAGATCA
actin1_r	AGGGCGTAACCCTCGTAGAT
actin2_f	AGATGCTGCCATCAGCGAA
actin2_r	AAGACTGGACCGACCTCAAG
TBP_f	AATAAACCCGGCGAACCTGT
TBP_r	TCGGCAGTAGAAATGGAGCC
SDHA_f	GGTACTGCCGTTCCTGTTGA
SDHA_r	ACTACGCTGAAGTGGCGAAA
