# Supplementary material for: Antennal transcriptome analysis of olfactory genes and characterizations of odorant binding proteins in two woodwasps, Sirex noctilio and Sirex nitobei (Hymenoptera: Siricidae)
Source: BMC Genomics. 2021 Mar 10;22:172. doi: 10.1186/s12864-021-07452-1 (PMC7945326; doi:10.1186/s12864-021-07452-1)
Supplement: Supplementary file 7 — Additional file 7. The OBPs, CSPs, ORs, SNMPs, GRs and IRs of Hymenoptera, Diptera, Lepidoptera, Coleoptera, Blattaria and Orthoptera used in building neighbor-joining phylogenetic trees. [file 12864_2021_7452_MOESM7_ESM.pdf]

Species	Name	Accession Number
Apis cerana cerana	OBP1	AEZ65022.1
Apis mellifera	ASP1	AAD51944.1
Apis mellifera	OBP8	NP_001164515.1
Apolygus lucorum	OBP5	AEA07663.1
Bombus impatiens	GOBP69a	XP_003493474.1
Bombus impatiens	GOBP19d	XP_012240863.1
Bombus terrestris	GOBP56d	XP_003397921.1
Bombus terrestris	GOBP69a	XP_003398556.1
Bombus terrestris	GOBP83a	XP_003401135.1
Bombyx mori	OBP23	NP_001153665.1
Bombyx mori	OBP26	NP_001159622.1
Bombyx mori	GOBP1	NP_001037496.1
Bombyx mori	GOBP2	NP_001037498.1
Bombyx mori	PBP1	CAA64443.1
Bombyx mori	PBP3	CAL47309.1
Bombyx mori	OBP8	BAI44701.1
Bombyx mori	OBP11	BAH36759.1
Bombyx mori	OBP12	NP_001153664.1
Bombyx mori	OBP14	CAS90125.1
Bombyx mori	OBP20	CAA64446.1
Cephus cinctus	OBP1	ARN17857.1
Cephus cinctus	OBP6	ARN17862.1
Cephus cinctus	OBP8	ARN17864.1
Cephus cinctus	OBP9	ARN17865.1
Cephus cinctus	OBP10	ARN17866.1
Drosophila melanogaster	OBP50c	NP_725387.3
Drosophila melanogaster	OBP50d	NP_725388.2
Drosophila melanogaster	OBP50a	NP_725385.1
Drosophila melanogaster	OBP47b	NP_610669.1
Drosophila melanogaster	OBP50e	NP_610959.2
Drosophila melanogaster	OBP49a	NP_610812.1
Drosophila melanogaster	OBP85a	NP_649802.2
Drosophila melanogaster	OBP46a	NP_610574.1
Drosophila melanogaster	OBP93a	NP_996254.1
Drosophila melanogaster	OBP58b	NP_611709.1
Drosophila melanogaster	OBP58c	NP_611710.1
Drosophila melanogaster	OBP59a	NP_788429.1
Drosophila melanogaster	PBP	NP_476990.1
Drosophila melanogaster	OBP19c	NP_608392.1
Drosophila melanogaster	OBP50b	NP_725386.1
Drosophila melanogaster	OBP58d	NP_611711.1
Drosophila melanogaster	OBP83ef	AAF51918.2
Drosophila melanogaster	OBP19d	ACY93964.1
Drosophila melanogaster	OBP28a	NP_523505.1
Drosophila melanogaster	OBP18a	NP_573350.1
Drosophila melanogaster	OBP56b	NP_611443.1
Drosophila melanogaster	OBP99a	NP_651707.1
Drosophila melanogaster	OBP99b	NP_651713.1
Drosophila melanogaster	OBP99c	NP_651711.1
Drosophila melanogaster	OBP44a	NP_610358.1
Drosophila melanogaster	OBP83g	NP_731043.1
Drosophila melanogaster	OBP8a	NP_727322.1
Drosophila melanogaster	OBP99d	NP_651712.1
Drosophila melanogaster	OBP83cd	AAF51919.2
Drosophila melanogaster	OBP19b	NP_608391.2
Drosophila melanogaster	OBP57d	NP_725973.1
Drosophila melanogaster	OBP57e	NP_611488.1
Drosophila melanogaster	OBP47a	NP_995810.1
Drosophila melanogaster	OBP56c	NP_725925.3
Drosophila melanogaster	OBP51a	NP_725436.1
Drosophila melanogaster	OBP56a	NP_611442.1
Drosophila melanogaster	OBP56d	NP_611444.2
Drosophila melanogaster	OBP56e	NP_611445.1
Drosophila melanogaster	OBP57a	NP_725966.1
Drosophila melanogaster	OBP57b	NP_725965.1
Drosophila melanogaster	OBP57c	NP_611481.1
Drosophila melanogaster	OBP22a	NP_722746.2
Drosophila melanogaster	OBP19a	NP_728338.2
Drosophila melanogaster	OBP83a	NP_524241.1
Drosophila melanogaster	OBP83b	NP_524242.2
Macrocentrus cingulum	OBP4	AQV03778.1
Macrocentrus cingulum	OBP2	ASM47934.1
Meteorus pulchricornis	OBP1	AQN78379.1
Meteorus pulchricornis	OBP2	AQN78380.1
Meteorus pulchricornis	OBP15	AQN78393.1
Meteorus pulchricornis	OBP16	AQN78394.1
Meteorus pulchricornis	OBP14	AQN78392.1

Species	Name	Accession Number
Meteorus pulchricornis	OBP9	AQN78387.1
Meteorus pulchricornis	OBP5	AQN78383.1
Microplitis mediator	OBP6	ABO15559.1
Microplitis mediator	OBP12	ANT46041.1
Microplitis mediator	OBP20	ANT46049.1
Microplitis mediator	OBP2	ABM05969.1
Microplitis mediator	PBP1	ABM05973.2
Microplitis mediator	OBP18	ANT46047.1
Orussus abietinus	GOBP72	XP_012274260.1
Orussus abietinus	GOBP69a	XP_012278867.1
Orussus abietinus	GOBP83a	XP_012281355.1
Osmia cornuta	OBP2	AGI05201.1
Osmia cornuta	OBP4	AGI05203.1
Osmia cornuta	OBP5	AGI05204.1
Schistocerca gregaria	OBP5	ATO59032.1
Sclerodermus sp. MQW-2015	OBP1	ALG36134.1
Sclerodermus sp. MQW-2015	OBP8	ALG36141.1
Sogatella furcifera	OBP1	AHB59655.1
Trichogramma dendrolimi	OBP10	ANG08500.1
Trichogramma dendrolimi	OBP5	ANG08495.1
Trichogramma japonicum	OBP7	ASA40281.1
Trichogramma japonicum	OBP9	ASA40283.1
Apis cerana cerana	CSP4	AFQ07769.1
Apis cerana cerana	CSP5	AFQ07770.1
Apis cerana cerana	CSP6	AFQ07771.1
Apis mellifera	CSP3	ABH88171.1
Apis mellifera	CSP4	ABH88172.1
Apis mellifera	CSP5	ABH88173.1
Apis mellifera	CSP6	ABH88174.1
Bombyx mori	CSP16	ABH88209.1
Bombyx mori	CSP15	ABH88208.1
Bombyx mori	CSP14	ABH88207.1
Bombyx mori	CSP13	ABH88206.1
Bombyx mori	CSP12	ABH88205.1
Bombyx mori	CSP11	ABH88204.1
Bombyx mori	CSP10	ABH88203.1
Bombyx mori	CSP9	ABH88202.1
Bombyx mori	CSP8	ABH88201.1
Bombyx mori	CSP7	ABH88200.1
Bombyx mori	CSP6	ABH88199.1
Bombyx mori	CSP5	ABH88198.1
Bombyx mori	CSP4	ABH88197.1
Bombyx mori	CSP3	ABH88196.1
Bombyx mori	CSP2	ABH88195.1
Bombyx mori	CSP1	ABH88194.1
Camponotus japonicus	CSP7	BAS29779.1
Cephus cinctus	CSP1	ARN17832.1
Cephus cinctus	CSP3	ARN17834.1
Cephus cinctus	CSP4	ARN17835.1
Cephus cinctus	CSP5	ARN17836.1
Cephus cinctus	CSP6	ARN17837.1
Drosophila melanogaster	CSP_B_38a	AAS64728.1
Drosophila melanogaster	CSP_B_38c	AAF53928.2
Drosophila melanogaster	CSP_B_38b	AAS64727.1
Drosophila melanogaster	CSP_B_42b	AAS64791.1
Drosophila melanogaster	CSP_B_42c	AAS64792.2
Drosophila melanogaster	CSP_B_53b	AAX52702.1
Drosophila melanogaster	CSP_A_98a	AAF56814.3
Drosophila melanogaster	CSP_B_42a	AAM70837.2
Drosophila melanogaster	CSP_B_93a	AAF55894.2
Drosophila melanogaster	CSP_B_93b	AAN13876.2
Drosophila melanogaster	CSP_B_74a	AAZ66059.1
Drosophila melanogaster	CSP_B_53a	AAX52701.1
Drosophila melanogaster	CSP_A_87a	AAF54934.1
Drosophila melanogaster	CSP_A_7a	AAF46258.2
Drosophila melanogaster	CSP_A_29a	AAO41170.1
Drosophila melanogaster	CSP_A_46a	AAZ52819.1
Drosophila melanogaster	CSP_A_56a	AAZ52804.1
Drosophila melanogaster	CSP_A_86a	AAZ52549.1
Drosophila melanogaster	CSP_A_84a	AAZ52518.1
Drosophila melanogaster	CSP_A_75a	AAF49268.2
Drosophila melanogaster	CSP2	CAG26929.1
Drosophila melanogaster	CSP1	CAG26928.1
Meteorus pulchricornis	CSP5	AQN78399.1
Meteorus pulchricornis	CSP6	AQN78400.1
Microplitis mediator	CSP1	ABO15560.1
Microplitis mediator	CSP2	ANT46050.1

Species	Name	Accession Number
Sclerodermus sp. MQW-2015	CSP3	ALG36156.1
Sclerodermus sp. MQW-2015	CSP5	ALG36158.1
Sclerodermus sp. MQW-2015	CSP6	ALG36159.1
Sclerodermus sp. MQW-2015	CSP7	ALG36160.1
Sclerodermus sp. MQW-2015	CSP8	ALG36161.1
Trichogramma dendrolimi	CSP1	ANG08515.1
Trichogramma dendrolimi	CSP2	ANG08516.1
Trichogramma dendrolimi	CSP6	ANG08520.1
Anoplophora glabripennis	ORco	XP_018568191.1
Apis cerana cerana	OR170	APW85808.1
Apis cerana cerana	OR2	PBC27756.1
Apis cerana cerana	OR1	PBC28594.1
Apis mellifera	OR2	AHJ37468.1
Apis mellifera	OR115	NP_001229918.1
Apis mellifera	OR170	NP_001229922.1
Apis mellifera	OR11	NP_001229891.1
Athalia rosae	ORco	XP_012253637.1
Atta colombica	OR1	KYM79613.1
Blattella germanica	OR76	PSN39644.1
Blattella germanica	OR67	PSN44116.1
Blattella germanica	OR48	PSN44388.1
Blattella germanica	OR49	PSN55634.1
Bombus impatiens	OR2	XP_003490544.1
Bombus impatiens	ORco	P_003494153.1_
Bombus impatiens	OR13a	XP_012241935.2
Bombus terrestris	OR82a	XP_003393433.2
Bombus terrestris	ORco	XP_003402775.1
Bombus terrestris	OR4	XP_012167493.1
Bombus terrestris	OR22c	XP_012167618.1
Bombus terrestris	OR2a	XP_012174904.1
Bombus terrestris	OR13a	XP_020722451.1
Bombus terrestris	OR47a	XP_020722486.1
Bombus terrestris	OR2	XP_020722597.1
Bombyx mori	OR8	NP_001157209.1
Bombyx mori	OR17	NP_001157210.1
Bombyx mori	OR19	NP_001091785.1
Bombyx mori	OR20	NP_001166605.1
Bombyx mori	OR21	NP_001104831.1
Bombyx mori	OR22	NP_001166613.1
Bombyx mori	OR45	NP_001104798.1
Bombyx mori	OR46	NP_001155299.1
Bombyx mori	OR47	NP_001104818.1
Bombyx mori	OR48	DAA34890.1
Bombyx mori	OR49b	XP_021203152.1
Bombyx mori	OR57	NP_001159625.1
Bombyx mori	OR58	NP_001166618.1
Bombyx mori	OR50	ACH73301.1
Bombyx mori	OR60	NP_001155301.1
Bombyx mori	OR10	NP_001104819.1
Bombyx mori	OR41	NP_001091787.1
Bombyx mori	OR16	NP_001104832.2
Bombyx mori	OR59	NP_001166611.1
Bombyx mori	OR40	NP_001166608.1
Bombyx mori	OR65	NP_001166622.1
Bombyx mori	OR14	NP_001166602.1
Bombyx mori	OR30	NP_001091786.1
Bombyx mori	OR33	NP_001103623.1
Bombyx mori	OR34	NP_001103624.1
Bombyx mori	OR18	NP_001166895.1
Bombyx mori	OR2	NP_001037060.1
Bombyx mori	OR63	NP_001166620.1
Camponotus japonicus	ORco	BAO48211.1
Cephus cinctus	OR2e	AGS43054.1
Cephus cinctus	OR3a	AGS43060.1
Cephus cinctus	OR1	ARN17880.1
Cephus cinctus	OR3	ARN17881.1
Cephus cinctus	OR18	ARN17891.1
Cephus cinctus	OR22	ARN17894.1
Cephus cinctus	OR23	ARN17895.1
Cephus cinctus	OR24	ARN17896.1
Cephus cinctus	OR34	ARN17905.1
Cephus cinctus	OR36	ARN17907.1
Cephus cinctus	OR37	ARN17908.1
Cephus cinctus	OR39	ARN17910.1
Cephus cinctus	OR41	ARN17912.1
Cephus cinctus	OR48	ARN17919.1
Cephus cinctus	OR50	ARN17921.1

Species	Name	Accession Number
Cephus cinctus	ORco	NP_001310774.1
Cephus cinctus	OR30a	XP_015605665.1
Cephus cinctus	OR4	XP_015607546.1
Cephus cinctus	OR45b	XP_015608438.1
Cephus cinctus	OR23a	XP_024942940.1
Drosophila melanogaster	OR22c	NP_523454.2
Drosophila melanogaster	OR10a	NP_511122.1
Drosophila melanogaster	OR45b	NP_523667.1
Drosophila melanogaster	OR24a	NP_523470.3
Drosophila melanogaster	OR56a	NP_523796.2
Drosophila melanogaster	OR74a	NP_524123.1
Drosophila melanogaster	OR35a	NP_723916.1
Drosophila melanogaster	OR88a	NP_524348.2
Drosophila melanogaster	OR69a	NP_996070.1
Drosophila melanogaster	OR43a	NP_523647.2
Drosophila melanogaster	OR49b	NP_523721.1
Drosophila melanogaster	OR30a	NP_523520.2
Drosophila melanogaster	OR67b	NP_524007.2
Drosophila melanogaster	OR63a	NP_523895.2
Drosophila melanogaster	OR83a	NP_524234.2
Drosophila melanogaster	OR1a	NP_525029.2
Drosophila melanogaster	OR45a	NP_523666.3
Drosophila melanogaster	OR82a	NP_730794.1
Drosophila melanogaster	OR9a	NP_511107.1
Drosophila melanogaster	OR47a	NP_523689.1
Drosophila melanogaster	OR83c	NP_524244.2
Drosophila melanogaster	OR67d	NP_648390.2
Drosophila melanogaster	OR65c	NP_729163.2
Drosophila melanogaster	OR65b	NP_729162.3
Drosophila melanogaster	OR65a	NP_729161.1
Drosophila melanogaster	OR47b	NP_523690.3
Drosophila melanogaster	OR83b	AAF52031.2
Drosophila melanogaster	OR13a	NP_523359.2
Drosophila obscura	OR13a	XP_022223310.1
Dufourea novaeangliae	OR1	KZC14707.1
Habropoda laboriosa	OR2	KOC70731.1
Helicoverpa armigera	ORco	XP_021195606.1
Locusta migratoria	OR3	ALD51378.1
Locusta migratoria	OR5	ALD51501.1
Macrocentrus cingulum	ORco	AGI62937.2
Manduca sexta	ORco	CUQ99422.1
Meteorus pulchricornis	OR1	AQN78403.1
Meteorus pulchricornis	OR2	AQN78404.1
Meteorus pulchricornis	OR4	AQN78406.1
Meteorus pulchricornis	OR5	AQN78407.1
Meteorus pulchricornis	OR32	AQN78434.1
Meteorus pulchricornis	OR33	AQN78435.1
Meteorus pulchricornis	OR35	AQN78437.1
Meteorus pulchricornis	OR36	AQN78438.1
Meteorus pulchricornis	OR58	AQN78460.1
Meteorus pulchricornis	OR74	AQN78476.1
Meteorus pulchricornis	OR83	AQN78485.1
Microplitis mediator	OR	ABM05966.1
Microplitis mediator	OR14	AKO89978.1
Microplitis mediator	OR21	AKO89985.1
Microplitis mediator	OR22	AKO89986.1
Microplitis mediator	OR27	AKO89991.1
Microplitis mediator	OR38	AKO90002.1
Microplitis mediator	OR39	AKO90003.1
Microplitis mediator	OR3	AGG17936.1
Microplitis mediator	OR7	AGG17940.1
Microplitis mediator	OR9	AGG17943.1
Microplitis mediator	OR40	AKO90004.1
Microplitis mediator	OR52	AKO90016.1
Nasonia vitripennis	OR81	NP_001164394.1
Nasonia vitripennis	OR293	NP_001164462.1
Nasonia vitripennis	OR1	NP_001164465.1
Nasonia vitripennis	OR76	NP_001164670.1
Nasonia vitripennis	OR77	NP_001164671.1
Nasonia vitripennis	OR45	NP_001177492.1
Nasonia vitripennis	OR78	NP_001177510.1
Nasonia vitripennis	OR192	NP_001177568.1
Nasonia vitripennis	OR245	NP_001177594.1
Nasonia vitripennis	OR294	NP_001177622.1
Nasonia vitripennis	OR296	NP_001177711.1
Orussus abietinus	ORco	XP_012273699.1
Orussus abietinus	OR47b	XP_012278569.2

Species	Name	Accession Number
Orussus abietinus	OR4	XP_012279133.1
Orussus abietinus	OR2a	XP_012284542.1
Orussus abietinus	OR13a	XP_012284546.1
Orussus abietinus	OR10a	XP_012284958.1
Plutella xylostella	ORco	NP_001296031.1
Trachymyrmex zeteki	OR1	KYQ46915.1
Aedes aegypti	SNMP2	ACK99698.1
Aedes aegypti	SNMP1	ACK99697.1
Agrotis ipsilon	SNMP2	AGF87120.1
Anoplophora chinensis	SNMP	AUF73093.1
Apis cerana cerana	SNMP	AGC91908.1
Apis mellifera	SNMP2	XP_026295811.1
Bombyx mori	SNMP1	NP_001037186.1
Bombyx mori	SNMP2	XP_012547405.1
Chilo suppressalis	SNMP2	AFS50074.1
Chilo suppressalis	SNMP1	AFS50073.1
Drosophila melanogaster	SNMP1	NP_650953.1
Drosophila melanogaster	SNMP2	ABW70129.1
Ectropis obliqua	SNMP1	AKN78948.1
Ectropis obliqua	SNMP2	ALS03935.1
Helicoverpa armigera	SNMP	AAO15604.1
Lasius niger	SNMP	KMQ89696.1
Manduca sexta	SNMP1	AAG49366.1
Manduca sexta	SNMP2	AAG49365.1
Meteorus pulchricornis	SNMP1	AQN78521.1
Ooceraea biroi	SNMP	EZA62031.1
Spodoptera litura	SNMP1	AGN48098.1
Spodoptera litura	SNMP2	AGN48099.1
Tribolium castaneum	SNMP1	EFA02899.2
Aedes aegypti	GR64	NP_001345543.1
Aedes aegypti	GR3	NP_001345057.1
Anomala corpulenta	GR4	AKC58581.1
Anopheles gambiae	GR24	ABK97614.1
Athalia rosae	GR24	XP_012260950.1
Athalia rosae	GR22	XP_012265848.2
Bombus impatiens	GR43a for sugar taste	XP_003486787.1
Bombus terrestris	GR43a for sugar taste	XP_012173302.1
Bombyx mori	GR22	XP_012551334.1
Bombyx mori	GR10	BAS18817.1
Bombyx mori	GR9	ACD85123.1
Bombyx mori	GR67	BAK52799.1
Bombyx mori	GR50	DAA06387.1
Bombyx mori	GR51	DAA06388.1
Bombyx mori	GR68	BAK52800.1
Bombyx mori	GR53	DAA06389.1
Bombyx mori	GR63	DAA06395.1
Bombyx mori	GR30	DAA06385.1
Bombyx mori	GR31	BAW33756.1
Bombyx mori	GR29	DAA06384.1
Bombyx mori	GR28	BAW33746.1
Bombyx mori	GR27	BAW33745.1
Bombyx mori	GR18	DAA06381.1
Bombyx mori	GR17	DAA06380.1
Bombyx mori	GR14	DAA06377.1
Bombyx mori	GR15	DAA06378.1
Bombyx mori	GR16	DAA06379.1
Bombyx mori	GR26	DAA06382.1
Bombyx mori	GR64	DAA06396.1
Bombyx mori	GR56	DAA06390.1
Bombyx mori	GR57	DAA06391.1
Bombyx mori	GR11	DAA06375.1
Bombyx mori	GR33	DAA06386.1
Bombyx mori	GR45	NP_001124346.1
Bombyx mori	GR58	DAA06392.1
Bombyx mori	GR62	DAA06394.1
Bombyx mori	GR61	DAA06393.1
Bombyx mori	GR60	ACD85127.1
Bombyx mori	GR8	NP_001124344.1
Bombyx mori	GR7	DAA06374.1
Bombyx mori	GR64a for sugar taste	XP_021208998.1
Cephus cinctus	GR24	XP_015584748.1
Cephus cinctus	GR64f for sugar taste	XP_015596523.1
Culex quinquefasciatus	GR24	XP_001848689.1
Drosophila obscura	GR43a for sugar taste	XP_022223635.1
Helicoverpa armigera	GR22	XP_021185659.1
Helicoverpa armigera	GR4	ASW18693.1
Microplitis mediator	GR6	AKO90019.1

Species	Name	Accession Number
Nasonia vitripennis	GR3	NP_001164386.1
Nilaparvata lugens	GR11	AUD08731.1
Orussus abietinus	GR5a for trehalose	XP_023290777.1
Orussus abietinus	GR43a for sugar taste	XP_012270798.1
Papilio xuthus	GR24	KPI99377.1
Pieris rapae	GR22	XP_022114189.1
Sclerodermus sp. MQW-2015	GR2	ALG36126.1
Sclerodermus sp. MQW-2015	GR3	ALG36127.1
Sclerodermus sp. MQW-2015	GR4	ALG36128.1
Trachymyrmex cornetzi	GR for trehalose	KYN18775.1
Lucilia cuprina	GR66a for bitter taste	XP_023307046.1
Ceratitis capitata	GR66a for bitter taste	XP_004525752.1
Ceratitis capitata	GR93a for bitter taste	XP_004530764.1
Acromyrmex echinatior	IR2	EGI60906.1
Aedes aegypti	IR8a	NP_001345179.1
Aedes aegypti	IR25a	NP_001345261.1
Apis cerana cerana	IR	PBC34947.1
Athalia rosae	IR2	XP_012263948.1
Blattella germanica	IR8a	PSN54615.1
Bombus impatiens	IR25a	XP_012241506.1
Bombus impatiens	IR2	XP_012247141.1
Bombus terrestris	IR25a	XP_012174802.1
Bombus terrestris	IR NMDA 2B	XP_020719424.1
Bombus terrestris	IR93a	XP_020722638.1
Bombyx mori	IR93a	XP_021203225.1
Bombyx mori	IR40a	XP_021202684.1
Bombyx mori	IR2	XP_004932640.2
Bombyx mori	IR1	XP_021208793.1
Cephus cinctus	IR2	ARN17848.1
Cephus cinctus	IR3	ARN17849.1
Cephus cinctus	IR6	ARN17852.1
Colaphellus bowringi	IR8a	ALR72538.1
Dendroctonus ponderosae	IR8a	AGI05169.1
Dendrolimus punctatus	IR8a	ARO70285.1
Drosophila melanogaster	IR8a	AAF46470.2
Drosophila melanogaster	IR25a	ADU79032.1
Drosophila melanogaster	IR93a	AAF55817.3
Drosophila melanogaster	IR76b	AAF49071.1
Drosophila melanogaster	IR21a	AAF51569.2
Drosophila melanogaster	IR68a	AAF50075.2
Drosophila melanogaster	IR11a	AAF48158.2
Drosophila melanogaster	IR7a	AAF46273.1
Drosophila melanogaster	IR7b	AAF46277.2
Drosophila melanogaster	IR7c	AAF46279.1
Drosophila melanogaster	IR7d	ACL82907.1
Drosophila melanogaster	IR7e	ACL82908.1
Drosophila melanogaster	IR7f	ACL82909.1
Drosophila melanogaster	IR7g	AAF46281.2
Drosophila melanogaster	IR87a	AAF54949.2
Drosophila melanogaster	IR67b	AAF50158.1
Drosophila melanogaster	IR60a	AAF47186.1
Drosophila melanogaster	IR92a	AAF55757.4
Drosophila melanogaster	IR76a	AAF49113.3
Drosophila melanogaster	IR100a	AAF57202.2
Drosophila melanogaster	IR85a	AAF54290.1
Drosophila melanogaster	IR62a	ABC66126.1
Drosophila melanogaster	IR51b	AAM68524.1
Drosophila melanogaster	IR56b	AAF57539.1
Drosophila melanogaster	IR52a	AAF58132.2
Drosophila melanogaster	IR52d	AAF58131.3
Drosophila melanogaster	IR54a	AAF57800.2
Drosophila melanogaster	IR56a	AAM68433.1
Drosophila melanogaster	IR94a	AAN13902.1
Drosophila melanogaster	IR47a	AAF58803.1
Drosophila melanogaster	IR84a	ADU79034.1
Drosophila melanogaster	IR75d	AAF49210.2
Drosophila melanogaster	IR75b	ACL83321.2
Drosophila melanogaster	IR75c	AAF49299.3
Drosophila melanogaster	IR75a	AAF49300.2
Drosophila melanogaster	IR64a	AAF50781.1
Dufourea novaeangliae	IR2	KZC14133.1
Habropoda laboriosa	IR2	KOC60411.1
Habropoda laboriosa	IR NMDA 3A	KOC61651.1
Locusta migratoria	IR25a	ALD51355.1
Locusta migratoria manilensis	IR8a	AKQ25182.1
Meteorus pulchricornis	IR64a	AQN78503.1
Microplitis mediator	IR8a	AKO90022.1

**Species**	**Name**	**Accession Number**
Microplitis mediator	IR64a	AKO90024.1
Musca domestica	IR25a	AFP89966.1
Ooceraea biroi	IR NMDA 3A	EZA62306.1
Orussus abietinus	IR5	XP_012273315.2
Orussus abietinus	IR2	XP_012273732.1
Orussus abietinus	IR93a	XP_012281340.1
Orussus abietinus	IR25a	XP_012287489.1
Pseudomyrmex gracilis	IR25a	XP_020287393.1
Schistocerca gregaria	IR8a	AHA80144.1
Trachymyrmex septentrionalis	IR2	KYN40142.1
Zootermopsis nevadensis	IR NMDA 2B	XP_021926766.1
