# Supplementary material for: Antennal transcriptome analysis of olfactory genes and characterizations of odorant binding proteins in two woodwasps, Sirex noctilio and Sirex nitobei (Hymenoptera: Siricidae)
Source: BMC Genomics. 2021 Mar 10;22:172. doi: 10.1186/s12864-021-07452-1 (PMC7945326; doi:10.1186/s12864-021-07452-1)
Supplement: Supplementary file 8 — Additional file 8: Selection of reference gene in S. noctilio and S. nitobei. Table S1. Normfinder result of the six reference genes in S. noctilio. Table S2. Normfinder result of the six reference genes in S. nitobei. Figure S1. Average expression stability values of remaining control genes in S. noctilio. Figure S2. Determination of the optimal number of control genes for normalization in S. noctilio. Figure S3. Average expression stability values of remaining control genes in S. nitobei. Figure S4. Determination of the optimal number of control genes for normalization in S. nitobei. [file 12864_2021_7452_MOESM8_ESM.pdf]

Table S1. Normfinder result of the six reference genes in *S. noctilio*

Gene name	Stability value
SDHA	0.793
TBP	0.397
Alpha-tubulin	0.233
Actin2	0.192
Actin1	1.529
Beta-tubulin	0.136
Best gene	Beta-tubulin

Table S2. Normfinder result of the six reference genes in *S. nitobei*

Gene name	Stability value
TBP	0.831
Actin1	0.335
Alpha-tubulin	0.321
Actin2	0.138
SDHA	1.242
Beta-tubulin	0.095
Best gene	Beta-tubulin

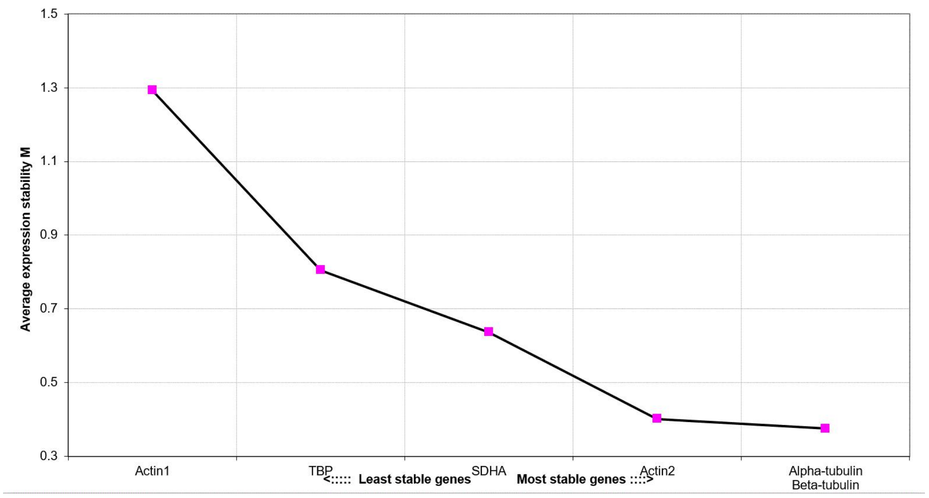

Figure S1. Average expression stability values of remaining control genes in *S. noctilio*

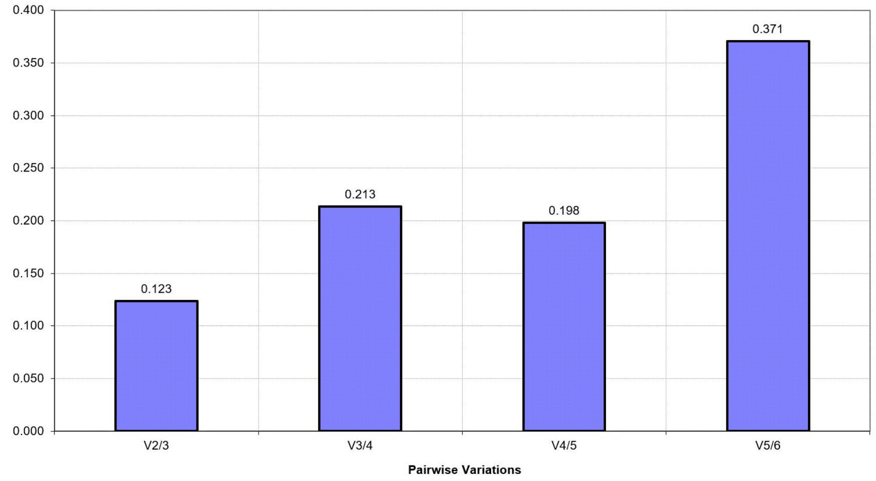

Figure S2. Determination of the optimal number of control genes for normalization in *S. noctilio*

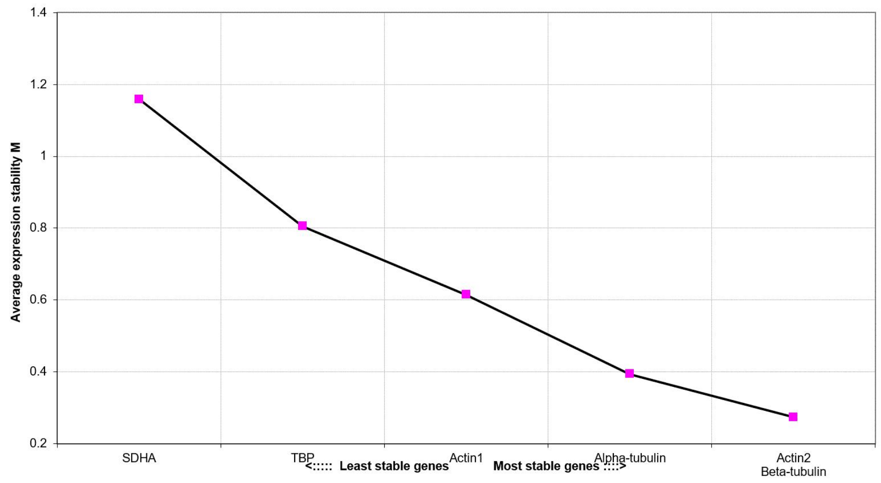

Figure S3. Average expression stability values of remaining control genes in *S. nitobei*

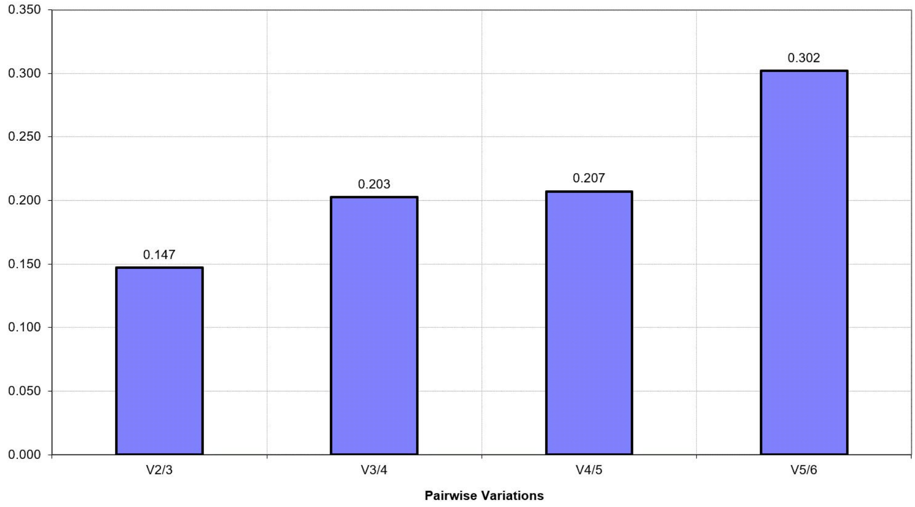

Figure S4. Determination of the optimal number of control genes for normalization in *S. nitobei*
